# Framework Development for Reducing Attrition in Digital Dietary Interventions: Systematic Review and Thematic Synthesis

**DOI:** 10.2196/58735

**Published:** 2024-08-27

**Authors:** Jian Wang, Jinli Mahe, Yujia Huo, Weiyuan Huang, Xinru Liu, Yang Zhao, Junjie Huang, Feng Shi, Zhihui Li, Dou Jiang, Yilong Li, Garon Perceval, Lindu Zhao, Lin Zhang

**Affiliations:** 1 The School of Public Health and Preventive Medicine Monash University Melbourne Australia; 2 Suzhou Industrial Park Monash Research Institute of Science and Technology Monash University Suzhou China; 3 The George Institute for Global Health University of New South Wales Sydney Australia; 4 The George Institute for Global Health Beijing China; 5 The Jockey Club School of Public Health and Primary Care Faculty of Medicine Chinese University of Hong Kong Hong Kong China (Hong Kong); 6 East China Aviation Personnel Medical Appraisal Center Shanghai China; 7 Tsinghua Vanke School of Public Health Tsinghua University Shenzhen China; 8 School of Economics and Management Southeast University Nanjing China

**Keywords:** thematic synthesis, attrition rate, dropout, behavior change theory, digital dietary intervention, digital health, mHealth, eHealth, mobile apps, email

## Abstract

**Background:**

Dietary behaviors significantly influence health outcomes across populations. Unhealthy diets are linked to serious diseases and substantial economic burdens, contributing to approximately 11 million deaths and significant disability-adjusted life years annually. Digital dietary interventions offer accessible solutions to improve dietary behaviors. However, attrition, defined as participant dropout before intervention completion, is a major challenge, with rates as high as 75%-99%. High attrition compromises intervention validity and reliability and exacerbates health disparities, highlighting the need to understand and address its causes.

**Objective:**

This study systematically reviews the literature on attrition in digital dietary interventions to identify the underlying causes, propose potential solutions, and integrate these findings with behavior theory concepts to develop a comprehensive theoretical framework. This framework aims to elucidate the behavioral mechanisms behind attrition and guide the design and implementation of more effective digital dietary interventions, ultimately reducing attrition rates and mitigating health inequalities.

**Methods:**

We conducted a systematic review, meta-analysis, and thematic synthesis. A comprehensive search across 7 electronic databases (PubMed, MEDLINE, Embase, CENTRAL, Web of Science, CINAHL Plus, and Academic Search Complete) was performed for studies published between 2013 and 2023. Eligibility criteria included original research exploring attrition in digital dietary interventions. Data extraction focused on study characteristics, sample demographics, attrition rates, reasons for attrition, and potential solutions. We followed ENTREQ (Enhancing the Transparency in Reporting the Synthesis of Qualitative Research) and PRISMA (Preferred Reporting Items for Systematic Reviews and Meta-Analyses) guidelines and used RStudio (Posit) for meta-analysis and NVivo for thematic synthesis.

**Results:**

Out of the 442 identified studies, 21 met the inclusion criteria. The meta-analysis showed mean attrition rates of 35% for control groups, 38% for intervention groups, and 40% for observational studies, with high heterogeneity (*I*²=94%-99%) indicating diverse influencing factors. Thematic synthesis identified 15 interconnected themes that align with behavior theory concepts. Based on these themes, the force-resource model was developed to explore the underlying causes of attrition and guide the design and implementation of future interventions from a behavior theory perspective.

**Conclusions:**

High attrition rates are a significant issue in digital dietary interventions. The developed framework conceptualizes attrition through the interaction between the driving force system and the supporting resource system, providing a nuanced understanding of participant attrition, summarized as insufficient motivation and inadequate or poorly matched resources. It underscores the critical necessity for digital dietary interventions to balance motivational components with available resources dynamically. Key recommendations include user-friendly design, behavior-factor activation, literacy training, force-resource matching, social support, personalized adaptation, and dynamic follow-up. Expanding these strategies to a population level can enhance digital health equity. Further empirical validation of the framework is necessary, alongside the development of behavior theory–guided guidelines for digital dietary interventions.

**Trial Registration:**

PROSPERO CRD42024512902; https://tinyurl.com/3rjt2df9

## Introduction

Dietary behavior significantly influences health across various populations [1]. Poor dietary habits are linked to serious diseases and substantial economic burdens [2]. Afshin et al [3] reported that dietary risks were responsible for approximately 11 million deaths and 255 million disability-adjusted life years in 2017. Additionally, unhealthy diets contribute significantly to noncommunicable diseases, which are projected to cost more than US $30 trillion globally in the next decade [4].

Implementing universally accessible dietary interventions is a common approach to improving dietary behaviors [5], and digital technology, known for its efficacy, reach, and affordability, presents promising solutions to the associated challenges [6]. However, attrition—defined as participant dropout before completing an intervention—is prevalent in digital health or eHealth [7-9]. In some formal evaluations of app-based health interventions, attrition rates have reached as high as 75%-99% [7,9]. Many factors contribute to this high attrition rate. For example, digital dietary interventions—dietary programs implemented via digital technology—involve factors such as insufficient motivation [10], lack of interest [11], time constraints [12], inadequate guidance [13], financial constraints [14], limited health care services [15], doubts about efficacy [13], health issues [16], technical problems [17], and overwhelming demands [18]. If attrition remains high, it significantly compromises the validity and reliability of such interventions [9]. Moreover, this influence is not limited to the individual level but also potentially exacerbates health disparities across different social groups—a manifestation of digital health inequity.

From the perspective of behavior theory, interventions aim to achieve behavior change, while attrition represents an interruption in this process. Behavior formation and development involve a multitude of factors, including individual factors (such as attitudes, self-efficacy, skills, and knowledge) and environmental factors (such as health care facilities, social networks, and policies) [19]. These factors are dynamic, arising from both fluctuations in the environment and the internal instability inherent within individuals [19]. When certain essential factors are lacking or insufficient in strength, it becomes impossible to maintain behavior change, leading to attrition [19,20]. Take dietary interventions for type 2 diabetes as an example: this is a long-term process aimed at promoting behavior change, requiring individual belief [21], self-efficacy [22], emotional support from family [23], nutrition advice from dietitians [24], accessible food environments [25,26], and supportive government policies [27]. Inadequate support from these factors can lead to discontinuation and participant attrition. When such attrition arises from common factors, like limited access to professional dietary guidance, it can result in population-level disruptions in behavior and health disparities, exemplifying health inequality [28,29]. Therefore, by viewing attrition as a multifactorial behavior disruption, and using behavior theories to identify the contributing factors and analyze their specific mechanisms, we can provide a novel perspective for understanding and addressing attrition and health disparities.

Nevertheless, research focusing on attrition, particularly investigations into its causes and potential solutions through the lens of behavior theories, remains sparse. This study aims to bridge this gap. Through systematic review and thematic synthesis [30], it comprehensively explores and summarizes the reasons for attrition and potential solutions. These findings are then integrated with concepts from multiple behavior theories to develop a comprehensive theoretical framework. This framework will not only elucidate the behavioral mechanisms behind attrition but also guide future work in designing and implementing more effective digital dietary interventions, thereby reducing attrition rates at the individual level and diminishing health inequalities at the population level.

## Methods

### Study Design

This study uses a systematic review, meta-analysis, and thematic synthesis to investigate participant attrition in digital dietary interventions. The study protocol is available on International Prospective Register of Systematic Reviews (PROSPERO; CRD42024512902). This review follows the ENTREQ (Enhancing the Transparency in Reporting the Synthesis of Qualitative Research) guidelines for reporting qualitative syntheses [31] and the PRISMA (Preferred Reporting Items for Systematic Reviews and Meta-Analyses) standards, including the PRISMA checklist in Multimedia Appendix 1.

### Search Strategy

A thorough search across 7 electronic databases, including PubMed, MEDLINE, Embase, CENTRAL, Web of Science, CINAHL Plus with Full Text, and Academic Search Complete, was conducted using a predefined set of search terms related to attrition in digital dietary interventions, including synonyms and British spellings, and performed as full-text searches. Examples of search terms used included disengagement rate, churn rate, turnover rate, dropout rate, noncompletion rate, attrition rate, retention rate, adherence rate, compliance rate, follow-up rate, and persistence rate. This aimed to identify relevant English-language studies published from 2013 to 2023. The search period was limited to the years 2013-2023 for 3 reasons. First, 2013 marked a pivotal year in the global mobile internet landscape with the widespread adoption of 4G LTE technology, mobile devices, and applications [32], which laid the foundation for the rapid growth of digital health technologies. Second, this timeframe ensured that the data and findings were current and reflective of the latest trends and methodologies in digital dietary interventions. Third, the volume and quality of research in this field have significantly increased in recent years, providing a robust body of literature for a comprehensive review. The search strategy (see Multimedia Appendix 2) was carefully developed and executed by our experienced research team, ensuring a systematic and thorough review of the literature.

### Eligibility Criteria

We focused on original research that either primarily or secondarily explored attrition rates in digital dietary interventions among human populations, encompassing both randomized controlled trials (RCTs) and observational studies. These interventions typically use technologies such as text messaging, social media, web-based platforms, smartphone apps, and personal digital assistants to improve dietary behaviors and support adherence to diet-related therapies, for example, managing chronic diseases and weight control [6,33-35]. Therefore, studies involving nondigital interventions were excluded. Additionally, nonoriginal studies, such as reviews, conference proceedings, commentaries, protocols, and collections, were excluded to concentrate on empirical data. Studies with minimal or unclear relevance to attrition rates were also omitted to ensure reliable data for thematic synthesis.

### Study Selection

Zotero 6 (Corporation for Digital Scholarship), a free, open-source research management tool, was used to assist in identifying duplicates and organizing papers. The initial screening of titles and abstracts was performed to eliminate nonoriginal research or studies not pertinent to digital dietary intervention attrition rates. Full-text assessments of potentially eligible studies were then conducted to determine their inclusion based on predefined criteria. This phase excluded studies with marginal or vague relevance to attrition rates. Two independent reviewers (FS and ZL), experienced in systematic reviews and digital health, conducted the selection. Any discrepancies were resolved through discussion or input from a third reviewer (DJ).

### Data Extraction

A standardized data abstraction form was developed by the research team to capture specific information from the included studies (see Multimedia Appendix 3). This form comprised 3 parts: the first part focused on study characteristics, including authors, year of publication, study date, targeted dietary behavior, duration, theories or behavioral techniques, study design, and intervention strategy. The second part covered sample characteristics, such as eligibility criteria, sample size, and demographics. The third part collected information relevant to attrition, including the number of participants, number of dropouts, attrition rates, reasons for attrition, and potential solutions. The form was pilot-tested on a sample of studies to ensure clarity and comprehensiveness.

Raw data on intervention strategies, reasons for attrition, and solutions to attrition were abstracted directly from the text of the included studies. This involved line-by-line extraction of relevant excerpts from the abstracts, results, and discussion sections of the included papers, which is the first step in thematic synthesis. In subsequent steps, these excerpts were organized and analyzed to identify descriptive and analytical themes (see “Analysis and Synthesis”).

Data extraction, carried out independently by 2 reviewers (XL and YZ) with backgrounds in public health and behavioral science, also sought consensus in the case of disagreements, facilitated by a third reviewer (JH), if required.

### Study Appraisal

Evaluating the quality of included studies is essential prior to thematic synthesis. This process determines each study's contribution to the synthesis process, referred to as its value to the synthesis [36]. In this study, qualitative content (data) on attrition causes and solutions was extracted from the included studies. These were consolidated into descriptive themes and subsequently synthesized into higher-level analytical themes through thematic synthesis. Therefore, assessing the strength of evidence for the descriptive themes is crucial before this process.

Although the included studies, including RCTs and observational studies, are quantitative, the evaluation focuses on the qualitative aspects of the research and, thus, should use qualitative appraisal tools. The method of Walsh and Downe [37] provides a comprehensive, flexible, and practical framework for appraising the quality of qualitative research, and it has been widely applied. Using this framework, we developed a question checklist and an evaluation form (see Multimedia Appendix 4) to appraise the quality of each study. The checklist facilitates the efficient and clear collection of essential evaluation information, while the evaluation form organizes the responses. These responses are used to assess the trustworthiness, transferability, and usefulness of each study based on 34 evaluation criteria, with points accumulated accordingly. The total score, with a perfect score of 34 points when all criteria are met, serves as the basis for rating the study’s value to the synthesis. Due to the small sample size of scores from 21 studies and their nonnormal distribution, we used the more efficient and straightforward 3-quartile method for rating. We calculated the first and second quartiles of the scores, which are 13.0 and 15.67, respectively. Based on these quartiles, we rated the studies as low (<13.0), moderate (13.0 ≤ score < 15.67), and high (≥15.67). One reviewer (YH) conducted the initial evaluation, and a second reviewer (WH) examined the ratings, with both being experienced in designing and evaluating behavior interventions. Discrepancies were resolved through discussion.

### Analysis and Synthesis

We conducted a meta-analysis to assess the attrition rates using RStudio (version 4.3.2; Posit) and a random effects model, treating attrition rates as the effect size. Studies from RCTs were categorized into control and intervention groups, while observational studies were classified as a separate group. Sensitivity analyses and publication bias assessments were performed separately for the intervention and observational groups to evaluate the robustness of results and detect potential reporting biases.

Thematic synthesis, involving the systematic extraction and synthesis of qualitative data from multiple studies, can offer deeper and comprehensive insights applicable across various contexts. We used this approach to synthesize descriptions of attrition reasons and solutions with NVivo (version 12; Lumivero). The process entailed: (1) line-by-line coding of data, (2) organizing codes into descriptive themes, and (3) refining themes into overarching analytical themes that elucidate attrition factors and interventions. Two experienced reviewers (JW and YL) in qualitative methods and evidence synthesis independently conducted all steps. After completing each step, they discussed interim results to reach a consensus before proceeding. This approach helped to identify and reduce discrepancies early, ensuring the reliability of the final results. Sensitivity analysis [38] tested the findings’ robustness by excluding low-quality study data and reassessing for consistent themes. This analysis confirmed the validity and robustness of our results.

Finally, concepts from multiple behavior theories were used to construct a conceptual framework that better explains the mechanisms of attrition. This approach introduced themes of personal agency from the integrated behavior model [20], habit or impulsive behavior from the reflective-impulsive model [39], cognition and reinforcement from social cognitive theory [40], and diverse resources from the conservation of resources theory [41]. Additionally, the perceived norms theme was expanded to include both subjective or injunctive and descriptive norms, aligning with the integrated behavior model.

## Results

### Search and Selection Results

**Figure 1:**
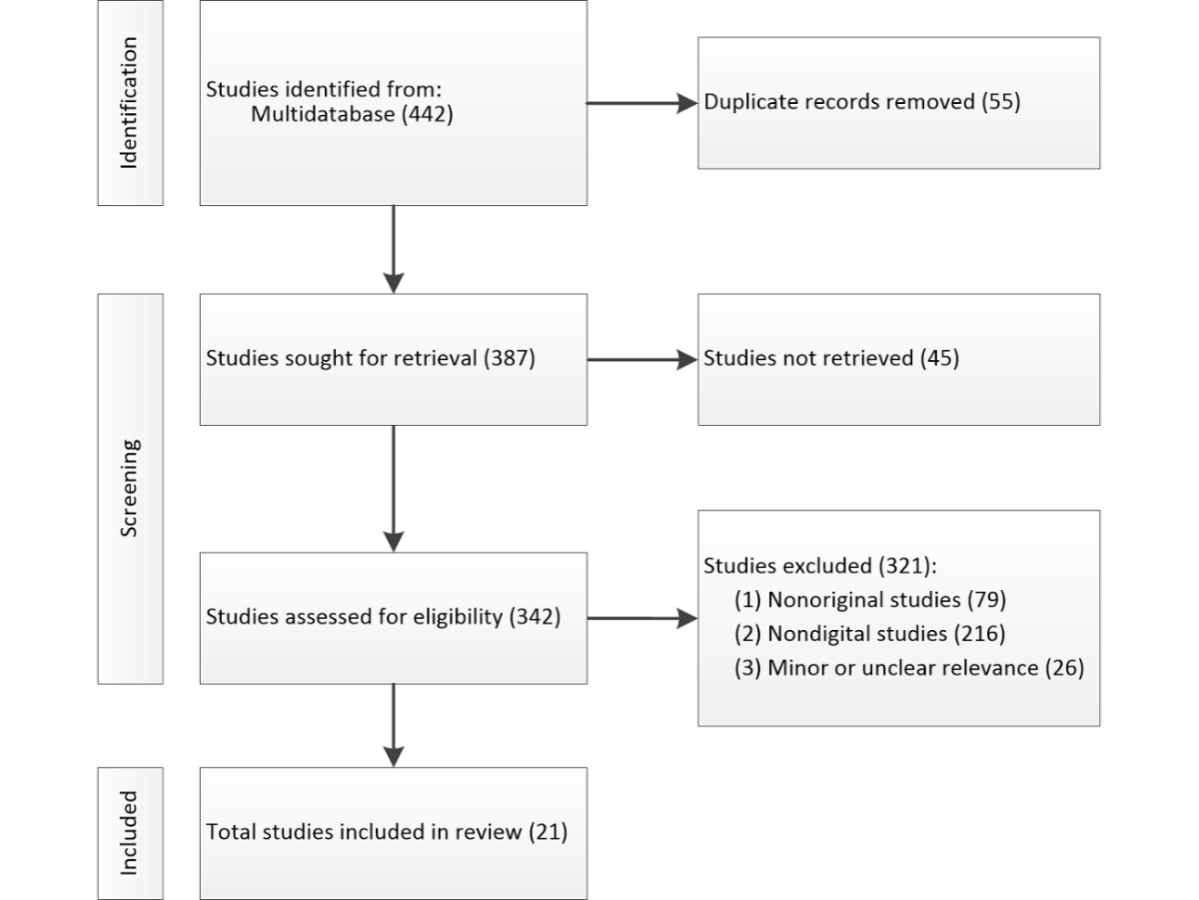
PRISMA (Preferred Reporting Items for Systematic Reviews and Meta-Analyses) flow diagram.

Database searches yielded 442 studies. After excluding 55 duplicates and 45 nonretrieved records (due to access restrictions, copyright limitations, or incomplete records), 342 underwent eligibility screening based on predetermined criteria, leading to the exclusion of 321 studies for not meeting inclusion requirements (details in Multimedia Appendix 5). Ultimately, 21 studies were included in the review (Table 1), with the selection process depicted in a PRISMA flow diagram (Figure 1).

**Table 1:** Summary of extracted study characteristics.

Author	Targeted dietary behavior	Attrition rate (%)	Design types
Brewer et al [11]	Increasing the intake of fruits and vegetables among the participants	—^a^	Randomized controlled trial
Browne et al [42]	Reducing the rate of eating among children with obesity	62.5	Randomized controlled trial
Cheung et al [43]	The intervention targeted dietary behaviors by including text messages that promoted Australian dietary guidelines after pregnancy. This included advice on controlling carbohydrate intake and the use of low carbohydrate vegetables and foods to satiate hunger.	52.5	Randomized controlled trial
Coa and Patrick [10]	Behaviors related to healthy eating	43.0	Observational study
Dawson et al [12]	Improving renal dietary behaviors related to potassium, phosphorus, sodium, and fluid intake, and general healthy eating and lifestyle behaviors.	10.3	Randomized controlled trial
Grutzmacher et al [15]	Nutrition and physical activity	14.3	Observational study
Hawkes et al [44]	Improving diet as one of its main objectives, alongside increasing physical activity and achieving weight loss, to reduce the risk of type 2 diabetes.	63.5	Observational study
Howarth et al [18]	Focusing on resilience, movement, eating, and sleep	—	Observational study
Jiang et al [45]	Targeting optimal nutritional intake using ordinary food and oral nutrition supplements (ONS), tailored to individual needs, preferences, and diet restrictions.	8.3	Randomized controlled trial
Kaul et al [13]	Tracking dietary intake and identifying dietary factors that might influence pain symptoms	38.9	Randomized controlled trial
Linardon et al [14]	Eating disorders, particularly behaviors related to binge eating.	66.0	Randomized controlled trial
Paxton et al [17]	Increasing the intake of fiber, fruits, vegetables, and reducing saturated and trans fats.	35.1	Observational study
Plaete et al [46]	Increasing the intake of fruit and vegetable to promote healthier dietary habits among adults	71.8	Randomized controlled trial
Rom et al [16]	The intervention targeted behaviors associated with binge-eating disorder, focusing on establishing regular eating patterns, self-monitoring of food intake, and addressing thoughts and feelings related to eating and body image.	26.3	Observational study
Schulz et al [8]	Reducing alcohol consumption among adult problem drinkers	52.7	Randomized controlled trial
Silina et al [47]	Increasing physical activity and dietary recommendations for dyslipidemia and weight loss	3.1	Randomized controlled trial
Springer et al [48]	Increasing fruit and vegetable intake	—	Randomized controlled trial
Van der Mispel et al [49]	Increasing fruit and vegetable intake	78.2	Observational study
Whitley et al [50]	Healthy eating and active living behaviors	4.6	Observational study
Young et al [51]	Mediterranean diet	70.3	Observational study
Yuhas et al [52]	Reducing the intake of SSBs^b^ by adolescents	11.5	Randomized controlled trial

^a^Not applicable.

^b^SSB: sugar-sweetened beverage.

### Study Characteristics and Appraisal

The characteristics of the included studies (details in Multimedia Appendix 6 [8,10-18,42-52]) span several countries: global (2 studies, subsequent numbers denote study counts), Latvia (n=1), Australia (n=4), the United States (n=9), Ireland (n=1), Germany (n=1), Belgium (n=2), and China (n=1). In total, 5 studies did not specify gender distribution, while the majority featured predominantly female participants. The interventions used varied digital technologies such as SMS, mobile apps, web-based programs, and email, targeting diverse dietary objectives including carbohydrate intake (n=1), fruit and vegetable intake (n=5), the Mediterranean diet (n=1), oral nutrition supplements intake (n=1), microelement and fluid intake (n=1), diet improvement for type 2 diabetes (n=1), binge eating (n=2), alcohol reduction (n=1), sugar-sweetened beverages intake (n=1), dietary factors related to pain symptoms (n=1), eating rate for obesity (n=1), and general healthy eating habits (n=5). Ethical considerations varied, with 2 studies not reporting on ethics, 1 bypassing review for involving voluntary workplace co-designers, and 18 obtaining clear ethical approvals. Behavior theories or techniques were used in 17 studies. Intervention durations ranged from 28 days to 4 months in 13 studies and 6-12 months in 7 studies, with 1 unspecified. Attrition rates surpassed 20% in 14 studies, peaking at 78.2%.

Among the studies, 12 were RCTs and 9 were observational. Attrition data collection methods varied, with 9 using existing digital systems, 7 detailing only calculation or timing methods, and 5 lacking clarification. Attrition causes and solutions were primarily derived from participant quotes, author interpretations, and trial evidence. Value to the synthesis faced challenges due to missing triangulation, theoretical saturation, representativeness exposition, alternative explanations, conceptual richness, and novel findings. Seven studies was rated high, 8 were rated moderate, and 6 were rated low, with the detailed appraisal process provided in Multimedia Appendix 7 [8,10-18,36,42-52].

### Meta-Analysis

**Figure 2:**
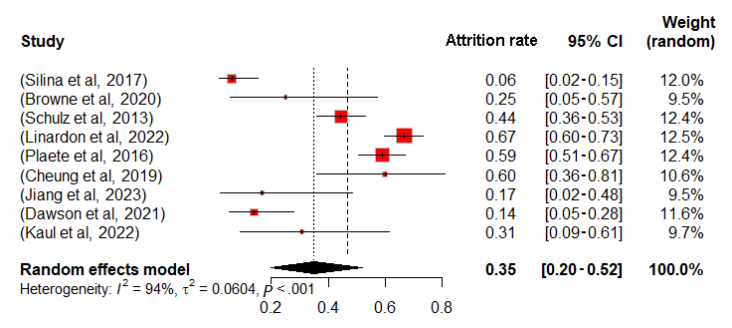
Forest plot of attrition rates for control group.

**Figure 3:**
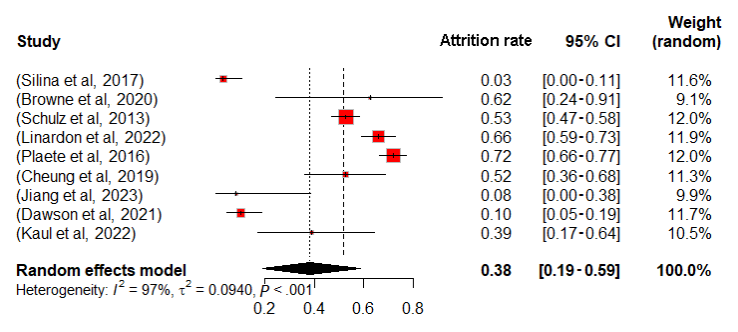
Forest plot of attrition rates for intervention group.

**Figure 4:**
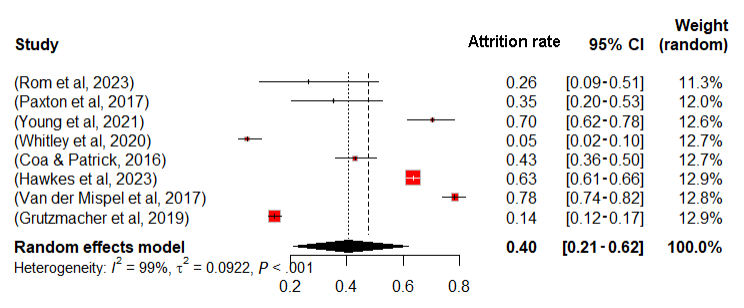
Forest plot of attrition rates for observation group.

Meta-analyses were conducted for control and intervention groups within 12 RCTs, and observational studies were analyzed as a separate group, using a random effects model in RStudio (detailed data in Multimedia Appendix 8 [8,10-17,42-52]). Missing data necessitated the exclusion of 4 studies, resulting in the analysis of 9 RCTs and 8 observational studies. Attrition rate served as the effect size, with results in Figures 2-4. The mean attrition rates were 35% (95% CI 20-52) for control, 38% (95% CI 19-59) for intervention, and 40% (95% CI 21-62) for observational groups. Heterogeneity was assessed using *I*² and *τ*², revealing high heterogeneity with *I*² values of 94%, 97%, and 99%, and *τ*² values of 0.0604, 0.0940, and 0.0922, respectively, all with *P*<.001. The *I*² statistic indicates the percentage of total variation across studies that is due to heterogeneity rather than chance. *τ*² represents the between-study variance, providing an estimate of the actual variation in effect sizes across the included studies. By using both *I*² and *τ*², we gain a comprehensive understanding of heterogeneity.

**Figure 5:**
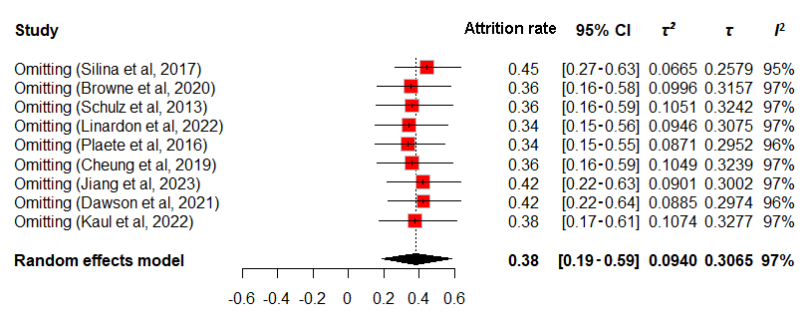
Sensitivity analysis of attrition rates in intervention group.

**Figure 6:**
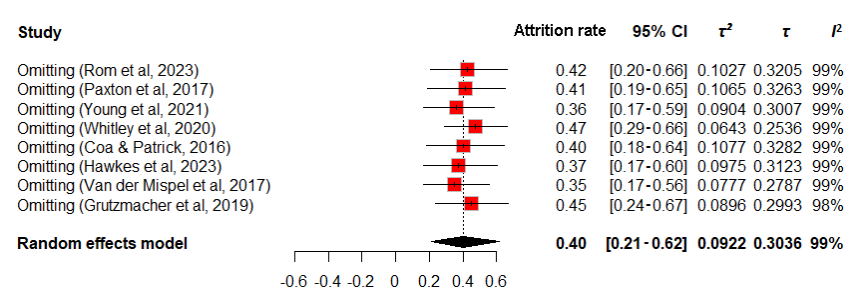
Sensitivity analysis of attrition rates in observational group.

Sensitivity analyses, shown in Figures 5-6, used a random-effects model, revealing attrition rates of 34%-45% for intervention groups and 35%-47% for observational groups. The slight variances in 95% CIs suggest that no individual study significantly alters the overall estimate. *τ*² and *τ* values indicated minimal dispersion and variation, with values ranging from 0.0665 to 0.1074 and 0.2579 to 0.3277 for intervention groups, and 0.0643 to 0.1077 and 0.2536 to 0.3282 for observational groups, respectively. Similarly, *I*² values, clustering around 95%-97% for the intervention group and remaining at 99% for the observational group, reflect substantial heterogeneity yet confirm the limited impact of individual studies on the overall results. These findings indicate that the meta-analysis results are stable and not significantly affected by the inclusion or exclusion of particular studies.

**Figure 7:**
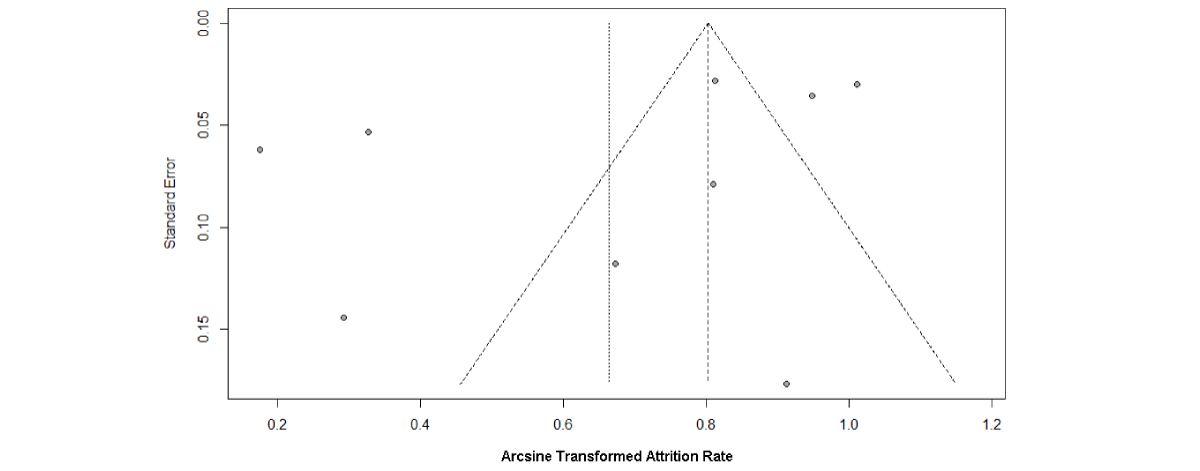
Funnel plot of attrition rate in intervention group.

**Figure 8:**
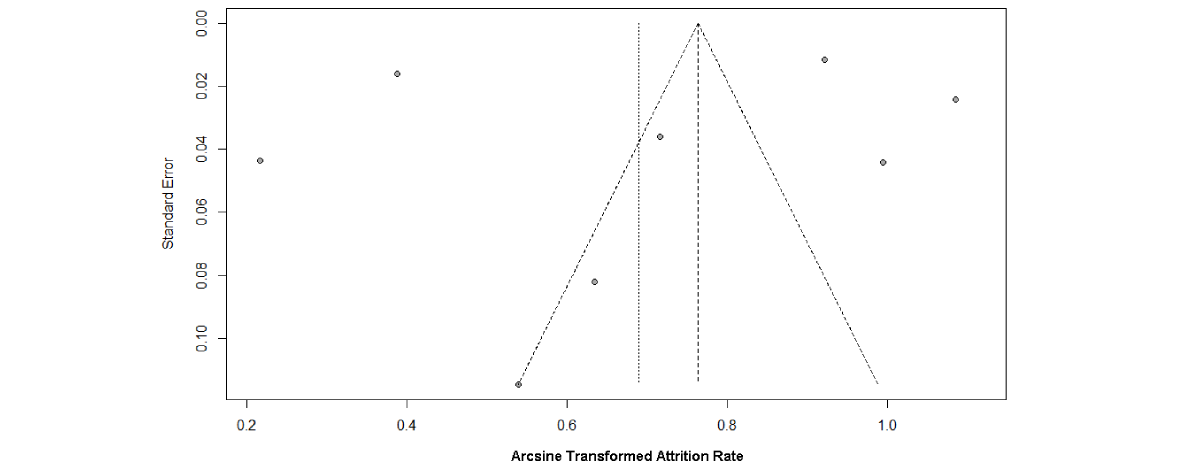
Funnel plot of attrition rates in observational group.

Publication bias was evaluated using Begg's and Egger's tests, alongside funnel plots (Figures 7-8). The intervention group's funnel plot suggested potential bias, though Begg's (*P*=.47) and Egger's (*P*=.20) tests were not statistically significant. The observational group's funnel plot showed more symmetry, implying less bias, supported by nonsignificant Begg's (*P*=.39) and Egger's (*P*=.70) test results. These findings indicate no significant publication bias, suggesting the meta-analysis relatively realistically reflect the actual situation.

### Thematic Synthesis

The thematic synthesis yielded 29 descriptive and 7 analytical themes, along with 19 subthemes, elucidating attrition reasons, as illustrated in Table 2. Furthermore, it identified 20 descriptive themes and 8 analytical themes, with 7 subthemes, addressing potential attrition solutions (details in Table 3). Evidence for each theme was extensively documented in Multimedia Appendix 9 [8,10,13-18,42-52], which also included descriptions not assigned to specific themes due to being nonspecific, unclear, or inefficient. Combining the themes of reasons and corresponding solutions from 2 tables, such as “motivation” with “boost and maintain motivation,” resulted in the formation of 13 merged themes. The finalized themes corresponded with the summary of Eysenbach [9] of factors influencing attrition rates (details in Multimedia Appendix 10).

**Figure 9:**
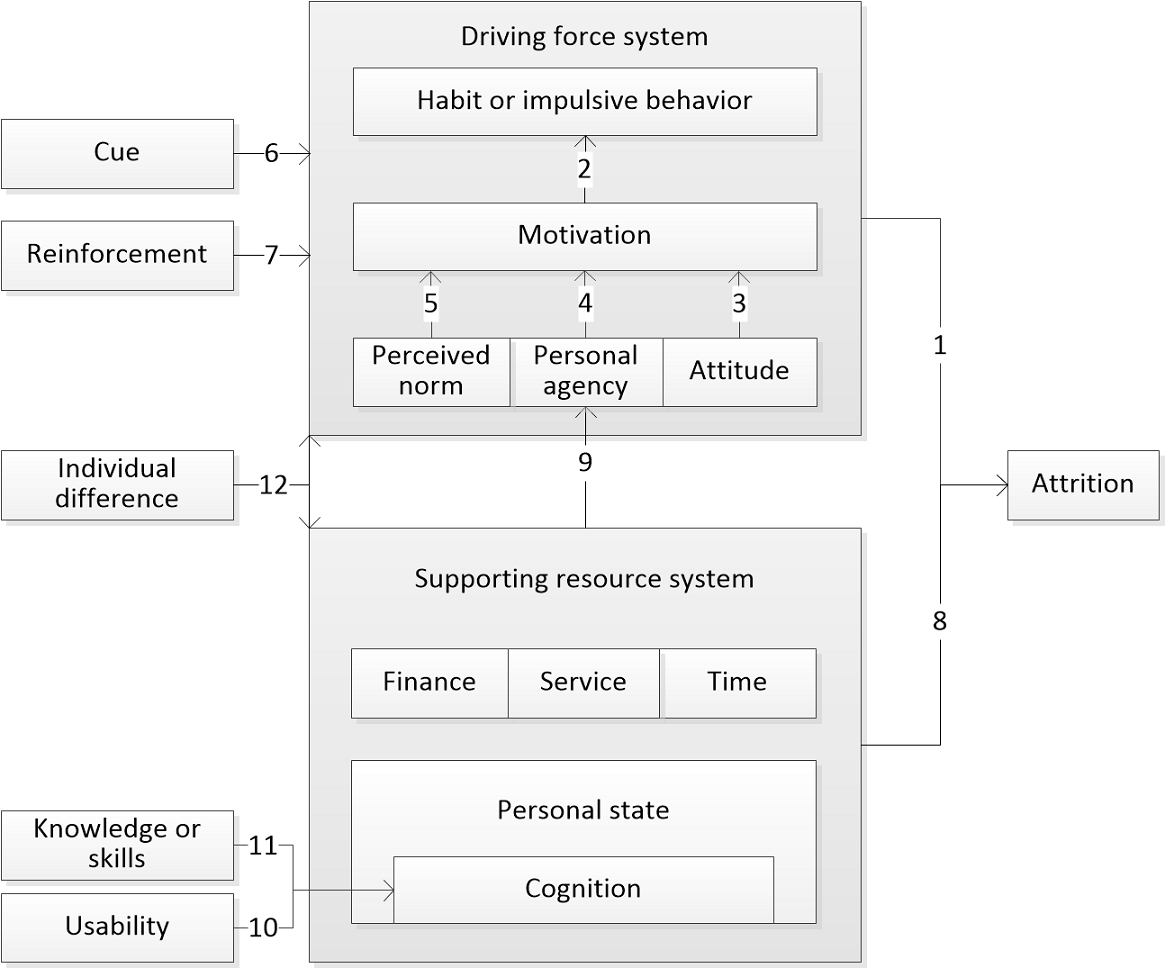
Force-resource model.

Subsequently, we drew on concepts from multiple behavior theories, resulting in 15 integrated themes. These themes were conceptually organized to elucidate their interconnections, as shown in Figure 9. This figure illustrates an explanatory framework where participant attrition is influenced by 2 main systems: the driving force system and the supporting resource system. For clarity, this integration is termed the force-resource model. The driving force system encompasses the inherent motivation or tendencies that determine the necessity of behavior execution. In contrast, the resource system provides the essential support required for the feasibility of implementing behaviors. This dichotomy leads to 2 primary causes of participant attrition: firstly, the failure of intervention strategies to generate sufficient motivation or to counteract risky habit or impulsive behaviors; secondly, the presence of inadequate or poorly matched resources. The discussion sections will provide a detailed exploration of these themes and their interplay within this conceptual framework.

**Table 2:** Themes of attrition reasons from included studies.

Analytical theme and analytical subtheme					Descriptive theme
**Motivation**					
	High motivation			Higher autonomous motivation Motivation waning	
**Attitude**					
	Negative experiential attitude			Lake of interest Strict timeline	
	Negative instrumental attitude			Limited usefulness Doubt regarding efficacy	
	Positive instrumental attitude			Goal-connection feeling Clearer expectation	
**Subjective or injunctive norm**					
	Lack of subjective or injunctive norm			No direct contact	
	With subjective or injunctive norm			With patient-provider relationship	
**Cue**					
	Distraction cue			Triggered by stop messages	
**Reinforcement**					
	Delayed reinforcement			Delayed feedback	
	Positive or immediate reinforcement			Positive feedback No reimbursement Immediate feedback	
**Resources**					
	**With cognitive load**			Overwhelmed tasks	
		Low usability	Technical or usability issues		
		Limited knowledge or skills	Limited guidance Limited technical literacy		
	Service resource			Lack of social support More health services Few health services	
	Financial resource			Financial barriers Higher income	
	Time resource			Time constraint	
	Personal state			Health or life issue	
**Individual differences**					
	Cultural factor			Cultural barriers	
	Education level			Low education level High education level	

**Table 3:** Themes of attrition solutions from included studies.

Analytical theme and analytical subtheme		Descriptive theme
Boost and maintain motivation		Enhance autonomous motivation Enhance self-affirmation
**Improve attitude**		
	Improve experiential attitude	Make interventions fun
	Improve instrumental attitude	Educate on intervention
Offer subjective or injunctive norm		Using health practitioners’ referrals
Provide immediate reinforcement		Set progress markers Provide immediate information
**Provide matching resources**		
	Decrease cognitive load	Make interventions easy Improve usability Provide guidance
	Provide financial resource	Address financial barriers
	Improve personal state	Improve emotional state
Provide social support		Using peer encouragement
Personalization strategy		Use targeted strategies
**Personalization strategy**		
	Based on feedback	Refine text-messages Solicit user-feedback
	Attend to individual difference	Screen participants Understand the impact of participants’ characteristics Address cultural barrier
Dynamic intervention		Provide tailored follow-up

## Discussion

### Principal Findings

We identified 15 interconnected theoretical themes and integrated behavior theory concepts to construct the force-resource model. As shown in Figure 9, the model comprises 2 subsystems that interact to influence behavior and contribute to attrition. The first subsystem, the driving force system, includes themes of motivation, perceived norms, personal agency, attitude, and habit or impulsive behavior, which collectively guide behavioral directions and trends. Participants are driven by this force system to engage in dietary interventions to improve health. Their behaviors are influenced by attitudes toward the diet-health connection and the efficacy of the intervention, as well as perceived norms and personal agency. This behavioral tendency underpins their initial participation and intention to persist.

The second subsystem is the supporting resources system. The core concept of this system is resource, defined as entities either intrinsically valued or instrumental in achieving valued ends. These resources include physical and psychological states, financial support, time availability, and accessible health services [41]. Adequate resources alongside the force system likely facilitate behavior change, whereas insufficient resources obstruct it. Interestingly, an excess of resources can also contribute to attrition, as it may diminish the perceived value of the intervention. This is attributable to the diverse and competitive nature of motivation; when resources are abundant, previously unattainable desires become attainable, leading participants to pursue more appealing activities, necessitating greater cognitive resources to overcome them. For instance, in food-rich environments, pursuing weight control goals as a self-regulation process demands more cognitive resources than pursuing eating enjoyment [53]. Therefore, ensuring participants have access to appropriate and ample supporting resources is critical to prevent attrition.

In addition to the 2 subsystems, the model includes other key components such as cues, which refer to specific environmental stimuli that trigger actions [54]; reinforcement, which increases the likelihood of a behavior by delivering a rewarding stimulus after the behavior [55]; participants’ knowledge and skills; the usability of digital interventions; and individual differences, primarily referring to stable personal attributes [56], including demographic backgrounds, personality traits, and cultural values.

For a more detailed elucidation of the components and mechanisms of the force-resource model, refer to Multimedia Appendix 11 [8,13,15,17,18,20,39,41-43,46,52-78].

### Implications for Digital Dietary Interventions

#### Overview

The force-resource model provides a resource-matching perspective for the design of digital dietary interventions, thus, forming multifaceted behavior intervention strategies tailored to the individual, based on the characteristics and functions of the components, and their interrelationships within the model.

#### User-Friendly Design

The design of digital interventions should ensure that processes are easy to understand and use, not only by avoiding excessive tasks, such as extensive questionnaires and record-keeping, but also by fully leveraging various technological tools to help simplify these tedious tasks and reduce cognitive load. For example, digital dietary interventions can use image processing and pattern recognition to streamline dietary recording [79], and use artificial intelligence to assess daily dietary quality automatically [80].

#### Behavior-Factor Activation

Based on behavior theory, behavior change can be achieved by activating corresponding components within the force system. Taking motivation as an example, improving diet is a gradual process that necessitates persistent adherence to achieve long-term health benefits, which presents significant challenges for behavior interventions based on health motivations. We advocate for the integration of health interventions with other activities such as gaming and social interaction that provide immediate feedback, thereby potentially enhancing sustained involvement and reducing attrition. Additionally, applying immediate reinforcement can increase the likelihood or probability of the behavior, while avoiding disruptive cues and highlighting beneficial ones can foster sustained engagement.

#### Literacy Training

The aim of providing targeted training programs is to enhance 2 types of literacy in individuals. The first is digital literacy, enabling them to use digital health resources more effectively with minimal cognitive load. The second is health literacy, which facilitates the activation of behavior factors such as attitudes and self-efficacy, thereby increasing receptiveness to health interventions. Additionally, there is a complementary relationship between literacy training and user-friendly design: the former helps people better operate digital tools with varying levels of user-friendliness, while interventions with good user-friendly design can reduce the demands on literacy training. Ideally, digital tools that effectively integrate cognitive psychology, behavioral science, and human-computer interaction are designed to be intuitive, engaging, and easy to use without requiring prior training.

#### Force-Resource Matching

Regarding the resource system, it is vital to ensure the provision or conservation of resources that are compatible with the force system. For instance, the availability of professional dietary counseling significantly influences the success rate of interventions for diabetic patients. Conversely, it is pragmatic to align motivation levels with available resources, recommending the setting of achievable dietary goals accordingly. For example, individuals with limited financial resources and access to medical advice should target a balanced diet as an intervention rather than pursuing antiaging diets or precision nutrition.

#### Social Support

Rooted in social networks [81], social support offers emotional, instrumental, and informational assistance [82]. Guidance and education fall under the category of informational support, which can alleviate cognitive processing demands while positively influencing attitudes, self-efficacy, and motivation. Emotional support has a wide-ranging impact, as it can enhance subjective well-being and cognitive functioning [83,84], as well as influencing attitudes [57,58], perceived norm [85], self-efficacy [86], and motivation [39,57,83,87,88]. Instrumental support facilitates access to financial and service-related resources, while fostering community and motivation, thus, reducing attrition risk [18,50].

#### Personalized Adaptation

There are significant variations in the characteristics and attributes of resource and force systems among individuals, arising not only from their personal state and circumstances but also from individual differences. Digital interventions should, therefore, tailor strategies to accommodate these variations, thereby enhancing resonance [89]. For instance, adapting dietary messages to reflect personal emotions and cultural eating habits can maintain engagement across diverse demographics [43]. Personalized adaptation is integral to multiple stages of digital interventions, including design, implementation, evaluation, and optimization, and artificial intelligence holds great potential in addressing this challenge.

#### Dynamic Follow-Up

The resource and force systems are dynamic, arising from fluctuations in both the environment and individuals’ internal states [57,90]. As mentioned earlier, environmental cues can easily trigger distractions and competing motivations, contributing to participant attrition. This implies that static interventions struggle to accommodate such variability. To address this issue, interventions should incorporate real-time adaptability, providing timely and tailored actions [91]. For instance, automated prompts encouraging re-engagement could redirect disengaging users back into the program if metrics indicate disengagement [45]. Additionally, for long-term monitoring of large populations, the rapid data processing capabilities of artificial intelligence can be fully used.

### Implications for Digital Health Equity

Participant attrition arises from mismatches between individual resource and force systems. When this phenomenon expands to population level, it essentially creates a form of digital health inequity due to disparities in access to digital health resources. Digital health equity strives for equitable access to and use of resources such as digital health technologies, training programs, digital health care systems, and community support structures, all designed to improve health outcomes universally [92,93]. In promoting digital health equity, reducing attrition rates is a key strategy [92-96]. This encourages us to broaden the goal of reducing attrition to encompass a larger population and to design solutions at more comprehensive levels based on the multilevel determinants in digital health [92,93]. We have proposed several strategic recommendations based on the force-resource model, as detailed in Multimedia Appendix 12 [92-96].

### Complementary Findings

Through a meta-analysis of attrition rates in digital dietary interventions over the past decade, we found that the average attrition rates ranged from 35% to 40%, representing a significant barrier to the efficacy and generalizability of such interventions, irrespective of the study design or the presence of an active intervention component. Notably, the intervention group exhibited a marginally higher attrition rate compared to the control group, with overlapping CIs, suggesting a lack of effectiveness of the investigated interventions in mitigating participant attrition in the included studies. This observation highlights the need for more potent and tailored strategies to promote sustained engagement.

The high degree of heterogeneities underscores the inherent complexity and diversity of factors influencing attrition rates in digital dietary interventions, including variations in study designs, intervention components, participant characteristics, and implementation contexts. This indicates significant room for improvement in standardization of digital dietary interventions. Taking participant characteristics as an example, some may join interventions out of curiosity without a genuine understanding or interest, making them unsuitable for the target group and likely to drop out quickly. These varied and mixed participant characteristics. The “run-in and withdrawal” strategy can mitigate this challenge by including an initial phase where all participants start the intervention [9]. This run-in phase helps identify those less likely to adhere. Participants not fully engaged or committed can then be excluded, leaving a more homogeneous and dedicated group for the remainder of the study. By ensuring a more homogeneous and committed participant group, and standardizing selection criteria, this strategy effectively reduces heterogeneity, thereby leading to more consistent and reliable assessments of intervention effects.

### Strengths

Most research on digital interventions prioritizes efficacy, often treating attrition rates as a mere data point rather than a subject of in-depth analysis. This review stands as the first to scrutinize attrition rates within digital systems through the lens of behavior theories, introducing a force-resource model to explore underlying causes and identify possible solutions. It also expands these findings to enhance digital health equity. The insights gained provide a foundational understanding and innovative strategies for developing more effective digital dietary interventions and promoting digital health equity.

### Limitations and Future Direction

First, despite performing a thorough search across 7 electronic databases, we acknowledge the limitation in capturing the entire body of literature related to attrition in digital dietary interventions due to the challenge of matching search terms precisely with indexed keywords. To mitigate this, we expanded the search terms and used a comprehensive full-text search strategy. However, there remains the potential for missing studies, as many do not specifically emphasize attrition, leading to its underrepresentation in indexed keywords. This limitation could affect the completeness of our review and underscores the need for more refined search methodologies in future research.

Second, the observed heterogeneities in meta-analysis outcomes highlight a critical issue stemming from significant study variances, emphasizing the need for enhanced standardization and harmonization of protocols and components in digital dietary interventions. Establishing behavior theory–guided guidelines and best practices for designing, implementing, and evaluating these interventions could lead to more consistent and replicable results, thereby improving their generalizability and impact.

Third, this study developed a theoretical framework to clarify attrition mechanisms and guide digital dietary interventions. However, due to the aforementioned limitations and the continuous introduction of new digital tools and strategies in this field, the framework's ability to encompass and explain currently undiscovered and future emerging scenarios remains uncertain. This necessitates further empirical validation and exploration in future research.

Last, reducing attrition rates is a key strategy for promoting digital health equity, encouraging us to expand the findings related to attrition reduction to the population level of digital health equity. However, some unique factors at the population level, such as cultural diversity, social structures, and communication patterns, with significant influence on digital health equity, have not been fully explored in this study. This highlights potential directions for future research.

### Conclusions

High attrition rates compromise the effectiveness and sustainability of digital dietary interventions. This review has pioneered the examination of participant attrition in such interventions through the lens of behavior theories, introducing the force-resource model. This framework conceptualizes attrition via the interaction between the driving force system and the supporting resource system, offering a nuanced understanding of participant attrition, summarized as insufficient motivation and inadequate or poorly matched resources. It highlights the critical necessity for digital dietary interventions to dynamically balance motivational components with available resources, ensuring interventions are both compelling and practically feasible for participants. Key recommendations include user-friendly design, behavior-factor activation, literacy training, force-resource matching, social support, personalized adaptation, and dynamic follow-up. These strategies are crucial for promoting sustained engagement and reducing attrition rates. Additionally, by expanding these strategies to a population level, this study contributes to the broader goal of promoting digital health equity.

The comprehensive and reliable validation of this framework requires further empirical investigation, especially with the continuous emergence of new digital tools and strategies in this field. Concurrently, developing behavior theory–guided guidelines for the design, implementation, and evaluation of digital dietary interventions is imperative to enhance their standardization and effectiveness. Furthermore, research should explore the impact of cultural diversity, social structures, and communication patterns on digital health interventions to ensure they are inclusive and effectively address the needs of diverse populations. This comprehensive approach will help overcome barriers to successful intervention outcomes and improve overall public health equity.

## Appendix

Multimedia Appendix 1 PRISMA (Preferred Reporting Items for Systematic Reviews and Meta-analyses) 2020 checklist.

Multimedia Appendix 2 Search strategies.

Multimedia Appendix 3 Standardized Data Abstraction Form.

Multimedia Appendix 4 Question Checklist and Evaluation Form for Study Quality Appraisal.

Multimedia Appendix 5 Papers Excluded From Analysis.

Multimedia Appendix 6 Study Characteristics.

Multimedia Appendix 7 Study Quality Appraisal.

Multimedia Appendix 8 Data for Meta-analysis.

Multimedia Appendix 9 Thematic Synthesis.

Multimedia Appendix 10 Analytical Themes and Eysenbach's Attrition Factors.

Multimedia Appendix 11 Force-Resource Model.

Multimedia Appendix 12 Implications for Digital Health Equity.

## Abbreviations

ENTREQ: Enhancing Transparency in Reporting the Synthesis of Qualitative Research
PRISMA: Preferred Reporting Items for Systematic Reviews and Meta-analyses
PROSPERO: International Prospective Register of Systematic Reviews
RCT: randomized controlled trial
