# Supplementary material for: Framework Development for Reducing Attrition in Digital Dietary Interventions: Systematic Review and Thematic Synthesis
Source: J Med Internet Res. 2024 Aug 27;26:e58735. doi: 10.2196/58735 (PMC11387916; doi:10.2196/58735)
Supplement: Multimedia Appendix 2 [file jmir_v26i1e58735_app2.doc]

**Multimedia Appendix 2: Search Strategies**

Seven databases were searched using consistent search terms and structure. The detailed search strategies for each database were provided below:

**PubMed and MEDLINE With PubMed Search Engine**

((digital) OR (eHealth) OR (mHealth) OR (mobile)) AND ((loss rate) OR (disengagement rate) OR (Churn Rate) OR (turnover rate) OR (dropout rate) OR (Non-completion Rate) OR (attrition rate) OR (retention rate) OR (adherence rate) OR (compliance rate) OR (follow-up rate) OR (persistence rate)) AND (health) AND (intervention) AND ((behavior change) OR (behaviour change)) AND ((diet) OR (dietary) OR (eating) OR (food) OR (eat)) Filters: Full text, Humans, English, MEDLINE, from 2013 - 2023

**Embase With Embase Search Engine**

(digital OR ehealth OR mhealth OR mobile) AND ((loss AND rate) OR (disengagement AND rate) OR (churn AND rate) OR (turnover AND rate) OR (dropout AND rate) OR ('non completion' AND rate) OR (attrition AND rate) OR (retention AND rate) OR (adherence AND rate) OR (compliance AND rate) OR ('follow up' AND rate) OR (persistence AND rate)) AND health AND intervention AND ((behavior AND change) OR (behaviour AND change)) AND (diet OR dietary OR eating OR food OR eat) AND ([article]/lim OR [article in press]/lim OR [conference abstract]/lim OR [conference paper]/lim OR [conference review]/lim OR [data papers]/lim OR [editorial]/lim OR [review]/lim OR [short survey]/lim) AND [humans]/lim AND [english]/lim AND [embase]/lim AND [2013-2023]/py

**CENTRAL With Cochrane Library**

Search scope: all text

Date published on the Cochrane Library: All dates

Content type: Cochrane Central Register of Controlled Trials (CENTRAL)

Date published on the Cochrane Library: 2013–2023

(digital OR eHealth OR mHealth OR mobile) AND ("loss rate" OR "disengagement rate" OR "Churn Rate" OR "turnover rate" OR "dropout rate" OR "Non-completion Rate" OR "attrition rate" OR "retention rate" OR "adherence rate" OR "compliance rate" OR "follow-up rate" OR "persistence rate") AND (health) AND (intervention) AND ("behavior change" OR "behaviour change") AND ((diet) OR (dietary) OR (eating) OR (food) OR (eat))

**CINAHL Plus With Full Text and Academic Search Complete With EBSCOhost**

((TX digital) OR (TX eHealth) OR (TX mHealth) OR (TX mobile)) AND ((TX loss rate) OR (TX disengagement rate) OR (TX Churn Rate) OR (TX turnover rate) OR (TX dropout rate) OR (TX Non-completion Rate) OR (TX attrition rate) OR (TX retention rate) OR (TX adherence rate) OR (TX compliance rate) OR (TX follow-up rate) OR (TX persistence rate)) AND (TX health) AND (TX intervention) AND ((TX behavior change) OR (TX behaviour change)) AND ((TX diet) OR (TX dietary) OR (TX eating) OR (TX food) OR (TX eat))

Limiters - Full Text; References Available; Publication Year: 20130101-20231231; Peer Reviewed; PDF Full Text; Language: English; Abstract Available; English Language; Research Article; Exclude Pre-CINAHL; Exclude MEDLINE Records; Evidence-Based Practice; Human; Publication Type: Journal Article; Publication Type: Academic Journal; Document Type: Article

Expanders - Also search within the full text of the articles; Apply equivalent subjects

Search Modes - Boolean/Phrase

**Web of Science With Web of Science Platform**

Languages: English

Document types: article, review article

Publication data: 2013–2023

((((((ALL=((digital) OR (eHealth) OR (mHealth) OR (mobile))) AND ALL=((loss rate) OR (disengagement rate) OR (Churn Rate) OR (turnover rate) OR (dropout rate) OR (Non-completion Rate) OR (attrition rate) OR (retention rate) OR (adherence rate) OR (compliance rate) OR (follow-up rate) OR (persistence rate))) AND ALL=(health)) AND ALL=(intervention)) AND ALL=((behavior change) OR (behaviour change))) AND ALL=((diet) OR (dietary) OR (eating) OR (food) OR (eat))) NOT ALL=(animal)
