# Supplementary material for: Framework Development for Reducing Attrition in Digital Dietary Interventions: Systematic Review and Thematic Synthesis
Source: J Med Internet Res. 2024 Aug 27;26:e58735. doi: 10.2196/58735 (PMC11387916; doi:10.2196/58735)
Supplement: Multimedia Appendix 3 [file jmir_v26i1e58735_app3.docx]

**Multimedia Appendix 3: Standardized Data Abstraction Form**

**Table 1.** Standardized data abstraction form.

| Data category | Specific data items | | | Study 1 | Study 2 | Note |
| --- | --- | --- | --- | --- | --- | --- |
| Study characteristics | Author | | |  |  |  |
|  | Year | | |  |  | Year of publication |
|  | Study date | | |  |  | Time of intervention implementation |
|  | Targeted dietary behavior | | |  |  | Targeted behavior (e.g., diets for weight loss, chronic disease management, or nutritional improvement) |
|  | Duration | | |  |  | Duration of intervention |
|  | Theories or behavioral techniques | | |  |  | Behavior theories or behavior change techniques involved in the intervention design and implementation |
|  | Study design | | |  |  | Randomized controlled trials or observational study |
|  | Randomized controlled trials | | Control group strategy |  |  | Strategies for control group |
|  |  |  | Intervention group strategy |  |  | Strategies for intervention group |
|  | Observational study | | Intervention strategy |  |  | Strategies for observational Study |
| Sample characteristics | Eligibility criteria | | |  |  | Eligibility criteria for participants |
|  | Country | | |  |  |  |
|  | Sample size | | |  |  |  |
|  | Age | | |  |  |  |
|  | Gender | | |  |  |  |
|  | Ethnicity | | |  |  |  |
|  | Socio-economic status | | |  |  |  |
|  | Other characteristics | | |  |  | Other notable features of the intervention study |
| Attrition-related information | Randomized controlled trial | Control group total (N) | |  |  | Total number of participants in control group |
|  |  | Control group loss (n) | |  |  | Number of dropouts in control group |
|  |  | Control group attrition rate (%) | |  |  | Attrition rate in control group, calculated as: Control group loss (n) / Control group total (N) × 100% |
|  |  | Intervention group total (N) | |  |  | Total number of participants in intervention group |
|  |  | Intervention group loss (n) | |  |  | Number of dropouts in intervention group |
|  |  | Intervention group attrition rate (%) | |  |  | Attrition rate in intervention group, calculated as: Intervention group loss (n) / Intervention group total (N) × 100% |
|  | Observational study | Observational study total (N) | |  |  | Total number of participants in observational study |
|  |  | Observational study loss (n) | |  |  | Number of dropouts in observational study |
|  |  | Observational study attrition rate (%) | |  |  | Attrition rate in observational study, calculated as: observational study loss (n) / observational study total (N) × 100% |
|  | Reason of attrition | | |  |  | Any viewpoints on attrition causes and solutions, derived from participant quotes, author interpretations, and trial evidence |
|  | Solutions of attrition | | |  |  |  |
