# Supplementary material for: Framework Development for Reducing Attrition in Digital Dietary Interventions: Systematic Review and Thematic Synthesis
Source: J Med Internet Res. 2024 Aug 27;26:e58735. doi: 10.2196/58735 (PMC11387916; doi:10.2196/58735)
Supplement: Multimedia Appendix 4 [file jmir_v26i1e58735_app4.doc]

**Multimedia Appendix 4: Question Checklist and Evaluation Form for Study Quality Appraisal**

**Questions Checklist**

1. What were the eligibility criteria for participants in this study?
2. Which country was the intervention population from?
3. What was the sample size of the intervention population?
4. What was the age distribution of the intervention population?
5. What was the gender distribution of the intervention population?
6. What was the ethnicity distribution of the intervention population?
7. What was the socioeconomic status of the intervention population, in terms of factors like social class and education level?
8. Were there any other relevant sample characteristics?
9. How was the intervention population recruited?
10. Regarding attrition rate data collection and analysis methods used in this study?
11. What attrition rate analysis methods and principles were used in this study?
12. What methods of triangulation were used for attrition rate analysis, such as participant validation of interpretations, multiple data collection or analysis methods, or involvement of multiple researchers in interpretation?
13. Regarding attrition rate analysis, was theoretical saturation declared?
14. How were the attrition rate findings linked to primary data? Did quotations clearly support interpretations? Was sufficient data presented? Were speakers identified to clarify quotes were not from only one or two participants?
15. Regarding reflexivity: Had the effect of the researcher on the study been considered? Had limitations been identified? Were ethical issues taken into account?
16. Regarding representativeness: How did the sample match the population described in the introduction or aims? How was the relationship of the sample to the broader population described?
17. Were other studies or theories cited to contextualize the attrition rate findings?
18. Was consideration given to diverse perspectives and contrary cases when explaining attrition rates?
19. Were the perspectives on attrition reasons and solutions conceptually rich?
20. Were there well-supported novel findings about attrition reasons and solutions?
21. Did the perspectives on attrition reasons and solutions focus on the population of interest?
22. Did the perspectives on attrition reasons and solutions connect to the review question?

Evaluation Form

| Subcategory | Criteria | Score | Question number |
| --- | --- | --- | --- |
| Transferability  (Based on sample characteristics) | Eligibility criteria | 1 | 1 |
|  | Country | 1 | 2 |
|  | Sample size | 1 point: ≤50  2 points: 51-100  3 points: 101-300  4 points: 301-500  5 points: 501-1000  6 points: >1000 | 3 |
|  | Age | 1 | 4 |
|  | Gender | 1 | 5 |
|  | Ethnicity | 1 | 6 |
|  | Socio-economic status | 1 | 7 |
|  | Other characteristics | 1 | 8 |
|  | **Subcategory total score** | 13 | / |
| Trustworthiness  (Focus on evidence of rigour) | Recruitment | 1 | 9 |
|  | Data collection | 1 | 10 |
|  | Analysis method and rationale | 1 | 11 |
|  | Triangulation methods | 3  1 point for meeting any of the 3 methods | 12 |
|  | Theoretical saturation | 1 | 13 |
|  | Linkage of findings to primary data | 3  1 point for meeting any of the 3 approaches | 14 |
|  | Reflexivity | 3  1 point for meeting any of the 3 criteria | 15 |
|  | Representativeness | 2  1 point for meeting any of the 2 criteria | 16 |
|  | Linkage to existing data/theory | 1 | 17 |
|  | Alternative explanations | 1 | 18 |
|  | **Subcategory total score** | 17 | / |
| Usefulness  (Focus on potential contribution to synthesis) | Conceptual richness | 1 | 19 |
|  | Novel findings | 1 | 20 |
|  | Focus on population of interest | 1 | 21 |
|  | Focus on attrition question | 1 | 22 |
|  | **Subcategory total score** | 4 | / |
| Value to the synthesis | **Total score** | 34 | / |
|  | **Rating** | **Low: < 13.0 Moderate: 13.0 ≤ score < 15.67 High: ≥ 15.67** | / |
