# Supplementary material for: Framework Development for Reducing Attrition in Digital Dietary Interventions: Systematic Review and Thematic Synthesis
Source: J Med Internet Res. 2024 Aug 27;26:e58735. doi: 10.2196/58735 (PMC11387916; doi:10.2196/58735)
Supplement: Multimedia Appendix 5 [file jmir_v26i1e58735_app5.doc]

**Multimedia Appendix 5: Papers Excluded From Analysis**

**Excluded Group 1: Reviews, Conference Proceedings, Commentaries, Protocols, Collections**

1. Aggarwal S, Patton G, Berk M, Patel V. Psychosocial interventions for self-harm in low-income and middle-income countries: Systematic review and theory of change. Social Psychiatry & Psychiatric Epidemiology; 2021 Oct;56(10):1729–1750. doi: 10.1007/s00127-020-02005-5
2. Åsberg K, Bendtsen M. Evaluating the effectiveness of a brief digital procrastination intervention targeting university students in Sweden: Study protocol for the focus randomised controlled trial; 2023 Jul;13(7):e072506. doi: 10.1136/bmjopen-2023-072506
3. Asberg K, Bendtsen M. Perioperative digital behaviour change interventions for reducing alcohol consumption, improving dietary intake, increasing physical activity and smoking cessation: A scoping review. Perioperative Med; 2021 Jul 6;10(1):18. doi: 10.1186/s13741-021-00189-1
4. Asiain J, Braun M, Roussos AJ. Virtual reality as a psychotherapeutic tool: current uses and limitations. British Journal of Guidance & Counselling; 2022 Feb;50(1):1–28. doi: 10.1080/03069885.2021.1885008
5. Barakat S, Maguire S, Smith KE, Mason TB, Crosby RD, Touyz S. Evaluating the role of digital intervention design in treatment outcomes and adherence to eTherapy programs for eating disorders: A systematic review and meta-analysis. International Journal of Eating Disorders; 2019 Oct;52(10):1077–1094. doi: 10.1002/eat.23131
6. Bikos LH, Dykhouse EC, Boutin SK, Gowen MJ, Rodney HE. Practice and research in career counseling and development-2012. Career Development Quarterly; 2013 Dec;61(4):290–329. doi: 10.1002/j.2161-0045.2013.00058.x
7. Billich N, Maugeri I, Calligaro L, Truby H, Davidson ZE. Weight management interventions that include dietary components for young people with chronic health care needs: A systematic review. NUTR DIET; 2022 Feb;79(1):94–109. doi: 10.1111/1747-0080.12698
8. Bomsta H, Sullivan CM. IPV survivors’ perceptions of how a flexible funding housing intervention impacted their children. Journal of Family Violence; 2018 Aug;33(6):371–380. doi: 10.1007/s10896-018-9972-5
9. Burton DGA, Wilmot C, Griffiths HR. Personalising nutrition for older adults: The InCluSilver project. NUTR BULL; 2018 Dec;43(4):442–455. doi: 10.1111/nbu.12356
10. Byaruhanga J, Atorkey P, McLaughlin M, Brown A, Byrnes E, Paul C, Wiggers J, Tzelepis F. Effectiveness of individual real-time video counseling on smoking, nutrition, alcohol, physical activity, and obesity health risks: Systematic review. J Med Internet Res; 2020 Sep 11;22(9):e18621. doi: 10.2196/18621
11. Campbell EJ, Lawrence AJ, Perry CJ. New steps for treating alcohol use disorder. Psychopharmacology; 2018 Jun;235(6):1759–1773. doi: 10.1007/s00213-018-4887-7
12. Cao X, Wong EML, Chow Choi K, Cheng L, Ying Chair S. Interventions for cardiovascular patients with type D personality: A systematic review. Worldviews Evid Based Nurs; 2016 Aug;13(4):314–323. doi: 10.1111/wvn.12153
13. Castro R, Ribeiro-Alves M, Oliveira C, Romero CP, Perazzo H, Simjanoski M, Kapciznki F, Balanza-Martinez V, De Boni RB. What are we measuring when we evaluate digital interventions for improving lifestyle? A scoping meta-review. Front Public Health; 2022 Jan 3;9:735624. doi: 10.3389/fpubh.2021.735624
14. Chiodelli R, Mello LTN de, Jesus SN de, Beneton ER, Russel T, Andretta I. Mindfulness-based interventions in undergraduate students: A systematic review. Journal of American College Health; 2022 Apr;70(3):791–800. doi: 10.1080/07448481.2020.1767109
15. Clough B, Yousif C, Miles S, Stillerova S, Ganapathy A, Casey L. Understanding client engagement in digital mental health interventions: An investigation of the eTherapy attitudes and process questionnaire. Journal of Clinical Psychology; 2022 Sep;78(9):1785–1805. doi: 10.1002/jclp.23342
16. Daud MH, Ramli AS, Abdul-Razak S, Isa MR, Yusoff FH, Baharudin N, Mohamed-Yassin MS, Badlishah-Sham SF, Nikmat AW, Jamil N, Mohd-Nawawi H. The EMPOWER-SUSTAIN e-health intervention to improve patient activation and self-management behaviours among individuals with metabolic syndrome in primary care: study protocol for a pilot randomised controlled trial. Trials; 2020 Dec;21(1):311. doi: 10.1186/s13063-020-04237-x
17. Denison-Day J, Appleton KM, Newell C, Muir S. Improving motivation to change amongst individuals with eating disorders: A systematic review. International Journal of Eating Disorders; 2018 Sep;51(9):1033–1050. doi: 10.1002/eat.22945
18. Dennis C-L, Marini F, Prioreschi A, Dol J, Birken C, Bell RC. The Canadian healthy life trajectories initiative (HeLTI) trial: a study protocol for monitoring fidelity of a preconception-lifestyle behaviour intervention. Trials; 2023 Apr 7;24(1):262. doi: 10.1186/s13063-023-07271-7
19. Dockray S, O’Neill S, Jump O. Measuring the psychobiological correlates of daily experience in adolescents. Journal of Research on Adolescence; 2019 Sep;29(3):595–612. doi: 10.1111/jora.12473
20. Dowling M, Hunter A, Biesty L, Meskell P, Conway A, O’Boyle G, Morrissey E, Houghton C. Driving and disabling factors of noncurative oral chemotherapy adherence: A qualitative evidence synthesis. Oncol Nurs Forum; 2019 Jan;46(1):16–28. doi: 10.1188/19.ONF.16-28
21. Giacobbi P, Hingle M, Johnson T, Cunningham JK, Armin J, Gordon JS. See me smoke-free: Protocol for a research study to develop and test the feasibility of an mHealth app for women to address smoking, diet, and physical activity. JMIR Res Protoc; 2016 Mar;5(1):e12. doi: 10.2196/resprot.5126
22. Gold N, Yau A, Rigby B, Dyke C, Remfry EA, Chadborn T. Effectiveness of digital interventions for reducing behavioral risks of cardiovascular disease in nonclinical adult populations: Systematic review of reviews. J Med Internet Res; 2021 May 14;23(5):e19688. doi: 10.2196/19688
23. Hamidzadeh A, Salehin S, Naseri Boori Abadi T, Chaman R, Mogharabian N, Keramat A. The effect of e-health interventions on meeting the needs of individuals with infertility: a narrative review. Middle East Fertility Society Journal; 2023 Apr; 28;28(1):1–15. doi: 10.1186/s43043-023-00137-7
24. Harrer M, Adam SH, Messner E, Baumeister H, Cuijpers P, Bruffaerts R, Auerbach RP, Kessler RC, Jacobi C, Taylor CB, Ebert DD. Prevention of eating disorders at universities: A systematic review and meta-analysis. International Journal of Eating Disorders; 2020 Jun;53(6):813–833. doi: 10.1002/eat.23224
25. Hebden L, Balestracci K, McGeechan K, Denney-Wilson E, Harris M, Bauman A, Allman-Farinelli M. “TXT2BFiT” a mobile phone-based healthy lifestyle program for preventing unhealthy weight gain in young adults: study protocol for a randomized controlled trial. Trials; 2013 Mar 18;14:75. doi: 10.1186/1745-6215-14-75
26. Henry J. The geopolitics of travel blogging. Geopolitics; 2021 May;26(3):817–837. doi: 10.1080/14650045.2019.1664473
27. Ifejika NL, Bhadane M, Cai CC, Noser EA, Grotta JC, Savitz S. Use of a smartphone-based mobile app for weight management in obese minority stroke survivors: Pilot randomized controlled trial with open blinded end point. JMIR Mhealth Uhealth; 2020 Apr 22;8(4):e17816. doi: 10.2196/17816
28. Jalongo MR. The effects of COVID-19 on early childhood education and care: Research and resources for children, families, teachers, and teacher educators. Early Childhood Education Journal; 2021 Sep;49(5):763–774. doi: 10.1007/s10643-021-01208-y
29. Jones LS, Russell A, Collis E, Brosnan M. To what extent can digitally-mediated team communication in children’s physical health and mental health services bring about improved outcomes? A systematic review. Child Psychiatry & Human Development; 2022 Oct;53(5):1018–1035. doi: 10.1007/s10578-021-01183-w
30. Kearney CA. Integrating systemic and analytic approaches to school attendance problems: Synergistic frameworks for research and policy directions. Child & Youth Care Forum; 2021 Aug;50(4):701–742. doi: 10.1007/s10566-020-09591-0
31. Khor B-H, Sumida K, Scholes-Robertson N, Chan M, Lambert K, Kramer H, Lui S-F, Wang AY-M. Nutrition education models for patients with chronic kidney disease. Seminars in Nephrology; 2023 Mar;43(2):151404. doi: 10.1016/j.semnephrol.2023.151404
32. Kipping R, Jago R, Metcalfe C, White J, Papadaki A, Campbell R, Hollingworth W, Ward D, Wells S, Brockman R, Nicholson A, Moore L. NAP SACC UK: Protocol for a feasibility cluster randomised controlled trial in nurseries and at home to increase physical activity and healthy eating in children aged 2–4 years; 2016;6(4):e010622. doi: 10.1136/bmjopen-2015-010622
33. Kotilahti E, West M, Isomaa R, Karhunen L, Rocks T, Ruusunen A. Treatment interventions for severe and enduring eating disorders: Systematic review. International Journal of Eating Disorders; 2020 Aug;53(8):1280–1302. doi: 10.1002/eat.23322
34. Krakouer J, Savaglio M, Taylor K, Skouteris H. Community-based models of alcohol and other drug support for First Nations peoples in Australia: A systematic review. Drug & Alcohol Review; 2022 Sep;41(6):1418–1427. doi: 10.1111/dar.13477
35. Li Y, Sun K, Wang B, Wang N, Luo R, Wang C, Liu Y, Duffy S, MacGregor GA, Wu J, He FJ, Zhang P. Comprehensive workplace intervention for cancer prevention in China (WECAN): Protocol for a stepped-wedge, cluster-randomised controlled trial. BMJ Open; 2023 Apr;13(4):e072405. doi: 10.1136/bmjopen-2023-072405
36. Linardon J, Messer M, Rodgers RF, Fuller-Tyszkiewicz M. A systematic scoping review of research on COVID-19 impacts on eating disorders: A critical appraisal of the evidence and recommendations for the field. International Journal of Eating Disorders; 2022 Jan;55(1):3–38. doi: 10.1002/eat.23640
37. Main A, Fuller W. Protein S-Palmitoylation: Advances and challenges in studying a therapeutically important lipid modification. FEBS Journal; 2022 Feb 15;289(4):861–882. doi: 10.1111/febs.15781
38. Mantel J. Refusing to treat noncompliant patients is bad medicine. Cardozo Law Review; 2017 Oct;39(1):127–197.
39. Marshall A, Bounds G, Patlovich K, Markham C, Farhat A, Cramer N, Oceguera A, Croom T, Carrillo J, Sharma S. Study design and protocol to assess fruit and vegetable waste at school lunches. Behav Sci Basel; 2019 Sep;9(9):101. doi: 10.3390/bs9090101
40. McMahon J, Thompson DR, Pascoe MC, Brazil K, Ski CF. eHealth interventions for reducing cardiovascular disease risk in men: A systematic review and meta-analysis. Preventive Medicine. 2021 Apr;145:106402. doi: 10.1016/j.ypmed.2020.106402
41. Medical dermatology. Australasian journal of Dermatology; 2022 May 2;63:44–98. doi: 10.1111/ajd.11_13832
42. Miler JA, Foster R, Hnizdilova K, Murdoch H, Parkes T. “It maybe doesn’t seem much, but to me it’s my kingdom”: Staff and client experiences of Housing First in Scotland. Drugs: Education, Prevention & Policy; 2022 Jun;29(3):231–244. doi: 10.1080/09687637.2021.1926429
43. Mitchell SA, Hoffman AJ, Clark JC, DeGennaro RM, Poirier P, Robinson CB, Weisbrod BL. Putting evidence into practice: An update of evidence-based interventions for cancer-related fatigue during and following treatment. Clin J Oncol Nurs; 2014 Dec 2;18:38–58. doi: 10.1188/14.CJON.S3.38-58
44. Moral-Munoz PhD JA, Zhang PhD W, Cobo PhD MJ, Herrera-Viedma PhD E, Kaber PhD DB. Smartphone-based systems for physical rehabilitation applications: A systematic review. Assist Technol; 2021 Jul;33(4):223–236. doi: 10.1080/10400435.2019.1611676
45. Morrow A, Walker K, Calder-MacPhee N, Ozakinci G. The active ingredients of physical activity and / or dietary workplace-based interventions to achieve weight loss in overweight and obese healthcare staff: A systematic review. Journal of Behavioral Medicine; 2022 Jun;45(3):331–349. doi: 10.1007/s10865-021-00279-x
46. Nicol G, Jansen M, Haddad R, Ricchio A, Yingling MD, Schweiger JA, Keenoy K, Evanoff BA, Newcomer JW. Use of an interactive obesity treatment approach in individuals with severe mental illness: Feasibility, acceptability, and proposed engagement criteria. JMIR Form Res; 2022 Dec;6(12):e38496. doi: 10.2196/38496
47. Oakley B, Loth E, Murphy DG. Autism and mood disorders. International Review of Psychiatry; 2021 May;33(3):280–299. doi: 10.1080/09540261.2021.1872506
48. Oliveira ACN, Guariente SMM, Zazula R, Mesas AE, Oliveira CEC, Reiche EMV, Nunes SOV. Hybrid and remote psychosocial interventions focused on weight and sedentary behavior management among patients with severe mental illnesses: A systematic review. Psychiatric Quarterly; 2022 Sep;93(3):813–840. doi: 10.1007/s11126-022-09994-3
49. Palmer M, Sutherland J, Barnard S, Wynne A, Rezel E, Doel A, Grigsby-Duffy L, Edwards S, Russell S, Hotopf E, Perel P, Free C. The effectiveness of smoking cessation, physical activity/diet and alcohol reduction interventions delivered by mobile phones for the prevention of non-communicable diseases: A systematic review of randomised controlled trials. PLoS One; 2018 Jan 5;13(1):e0189801. doi: 10.1371/journal.pone.0189801
50. Pfefferbaum B, Weems C, Scott B, Nitiéma P, Noffsinger M, Pfefferbaum R, Varma V, Chakraburtty A. Research methods in child disaster studies: A review of studies generated by the September 11, 2001, Terrorist Attacks; the 2004 Indian Ocean Tsunami; and Hurricane Katrina. Child & Youth Care Forum; 2013 Aug;42(4):285–337. doi: 10.1007/s10566-013-9211-4
51. Poster Session II. Bipolar Disorders; 2020 Jun 2;22:93–124. doi: 10.1111/bdi.12939
52. Poster Session II. Bipolar Disorders; 2021 Jun 2;23:78–101. doi: 10.1111/bdi.13098
53. Poster. Bipolar Disorders; 2019 Mar 2;21:66–147. doi: 10.1111/bdi.12746
54. POSTERS—16th conference clinical trials alzheimer’s disease, October 24-27 2023, Boston, MA, USA. J Prev Alz Dis; 2022. doi: 10.14283/jpad.2022.130
55. Poulton R, Moffitt T, Silva P. The dunedin multidisciplinary health and development study: Overview of the first 40 years, with an eye to the future. Social Psychiatry & Psychiatric Epidemiology; 2015 May;50(5):679–693. doi: 10.1007/s00127-015-1048-8
56. Presseller EK, Lampe EW, Zhang F, Gable PA, Guetterman TC, Forman EM, Juarascio AS. Using wearable passive sensing to predict binge eating in response to negative affect among individuals with transdiagnostic binge eating: Protocol for an observational Study. JMIR Res Protoc; 2023;12:e47098. doi: 10.2196/47098
57. Quinn É, Hynes SM. Occupational therapy interventions for multiple sclerosis: A scoping review. Scand J Occup Ther; 2021 Jul;28(5):399–414. doi: 10.1080/11038128.2020.1786160
58. Ramshaw G, McKeown A, Lee R, Conlon A, Brown D, Kennedy PJ. Introduction of technology to support young people’s care and mental health—A rapid evidence review. Child & Youth Care Forum; 2023 Jun;52(3):509–531. doi: 10.1007/s10566-022-09700-1
59. Redfern J, Hyun K, Singleton A, Hafiz N, Raeside R, Spencer L, Carr B, Caterson I, Cullen J, Ferry C, Santo K, Hayes A, Leung RWM, Raadsma S, Swinbourne J, Cho JG, King M, Roberts M, Kok C, Jenkins C, Chow C. ITM support for patients with chronic respiratory and cardiovascular diseases: A protocol for a randomised controlled trial. BMJ Open; 2019 Jun;9(3):e023863. doi: 10.1136/bmjopen-2018-023863
60. Riches SP, Piernas C, Aveyard P, Sheppard JP, Rayner M, Jebb SA. The salt swap intervention to reduce salt intake in people with high blood pressure: Protocol for a feasibility randomised controlled trial. Trials; 2019 Oct 11;20(1). doi: 10.1186/s13063-019-3691-y
61. Russell MA, Gajos JM. Annual research review: Ecological momentary assessment studies in child psychology and psychiatry. Journal of Child Psychology & Psychiatry; 2020 Mar;61(3):376–394. doi: 10.1111/jcpp.13204
62. Savaglio M, O’Donnell R, Hatzikiriakidis K, Vicary D, Skouteris H. The impact of community mental health programs for Australian youth: A systematic review. Clinical Child & Family Psychology Review; 2022 Sep;25(3):573–590. doi: 10.1007/s10567-022-00384-6
63. Schoenthaler A, Cruz J, Payano L, Rosado M, Labbe K, Johnson C, Gonzalez J, Patxot M, Patel S, Leven E, Mann D. Investigation of a mobile health texting tool for embedding patient-reported data into diabetes management (i-Matter): Development and usability study. JMIR Form Res; 2020 Aug;4(8):e18554. doi: 10.2196/18554
64. Schultz CL, Bocarro JN, Hipp JA, Bennett GJ, Floyd MF. Prescribing time in nature for human health and well-being: Study protocol for tailored park prescriptions. Front Digit Health; 2022 Jul 19;4:932533. doi: 10.3389/fdgth.2022.932533
65. Scott SE, Duarte C, Encantado J, Evans EH, Harjumaa M, Heitmann BL, Horgan GW, Larsen SC, Marques MM, Mattila E, Matos M, Mikkelsen M-L, Palmeira AL, Pearson B, Ramsey L, Sainsbury K, Santos I, Sniehotta F, Stalker C, Teixeira P, Stubbs RJ. The NoHoW protocol: A multicentre 2×2 factorial randomised controlled trial investigating an evidence-based digital toolkit for weight loss maintenance in European adults. BMJ Open; 2019 Sep;9(9):e029425. doi: 10.1136/bmjopen-2019-029425
66. Sedeh FB, Arvid Simon Henning M, Mortensen OS, Jemec GBE, Ibler KS. Communicating with patients through pictograms and pictures—a scoping review. Journal of Dermatological Treatment; 2022 Sep;33(6):2730–2737. doi: 10.1080/09546634.2022.2068790
67. Ski CF, Mcmahon J, Mcmahon J, Brazil K, Brazil K, Thompson DR, Thompson DR. ManGuard an eHealth intervention to reduce cardiovascular risk in male taxi drivers: A pilot randomised controlled trial protocol. European Journal of Cardiovascular Nursing; 2022 Jul 2;21(Supplement_1):zvac060.040. doi: 10.1093/eurjcn/zvac060.040
68. Smith KE, Mason TB, Juarascio A, Schaefer LM, Crosby RD, Engel SG, Wonderlich SA. Moving beyond self-report data collection in the natural environment: A review of the past and future directions for ambulatory assessment in eating disorders. International Journal of Eating Disorders; 2019 Oct;52(10):1157–1175. doi: 10.1002/eat.23124
69. Sung JY, Kacmarek CN, Schleider JL. Economic evaluations of mental health programs for children and adolescents in the United States: A systematic review. Clinical Child & Family Psychology Review; 2021 Mar;24(1):1–19. doi: 10.1007/s10567-020-00333-1
70. Thunnissen MR, aan het Rot M, van den Hoofdakker BJ, Nauta MH. Youth psychopathology in daily life: Systematically reviewed characteristics and potentials of ecological momentary assessment applications. Child Psychiatry & Human Development; 2022 Dec;53(6):1129–1147. doi: 10.1007/s10578-021-01177-8
71. Turan Kavradim S, Özer Z, Boz İ. Effectiveness of telehealth interventions as a part of secondary prevention in coronary artery disease: A systematic review and meta-analysis. SCAND J CARING SCI; 2020 Sep;34(3):585–603. doi: 10.1111/scs.12785
72. Varela C, Saldana C. En_Linea. An online treatment to change lifestyle in overweight and obesity: Study protocol for a randomized controlled trial. BMC Public Health; 2019 Nov 21;19(1):1552. doi: 10.1186/s12889-019-7928-1
73. Vashishtha R, Pennay A, Dietze PM, Livingston M. Trends in adolescent alcohol and other risky health- and school-related behaviours and outcomes in Australia. Drug & Alcohol Review; 2021 Sep;40(6):1071–1082. doi: 10.1111/dar.13269
74. Vos T, Lim SS, Abbafati C, Abbas KM, Abbasi M, Abbasifard M, et al. Global burden of 369 diseases and injuries in 204 countries and territories, 1990–2019: A systematic analysis for the Global Burden of Disease Study 2019. The Lancet; 2020 Oct;396(10258):1204–1222. doi: 10.1016/S0140-6736(20)30925-9
75. Wiechert M, Holzapfel C. Nutrition concepts for the treatment of obesity in adults. Nutrients; 2022 Jan;14(1):169. doi: 10.3390/nu14010169
76. Willis E, Burney RO, Hales DF, Ilugbusi L, Tate DC, Nezami BH, Clarke E, Moore R, Mathews E, Thompson MS, Beckelheimer B, Ward D. "My wellbeing-their wellbeing "—An eHealth intervention for managing obesity in early care and education: Protocol for the Go NAPSACC Cares cluster randomized control trial. PLoS One; 2023 Jul 7;18(7):e0286912. doi: 10.1371/journal.pone.0286912
77. Wray TB, Merrill JE, Monti PM. Using Ecological Momentary Assessment (EMA) to assess situation-level predictors of alcohol use and alcohol-related consequences. Alcohol Research: Current Reviews National Institute on Alcohol Abuse & Alcoholism. 2014 Jan;36(1):19–27.
78. Wray-Lake L, Abrams LS. Pathways to civic engagement among urban youth of color. Monographs of the Society for Research in Child Development; 2020 Jun;85(2):7–154. doi: 10.1111/mono.12415
79. Yang H, Kang J-H, Kim O, Choi M, Oh M, Nam J, Sung E. Interventions for preventing childhood obesity with smartphones and wearable device: A protocol for a non-randomized controlled trial. IJERPH; 2017 Feb 13;14(2):184. doi: 10.3390/ijerph14020184

**Excluded Group 2: Without Covering of Attrition in Human Digital Dietary Interventions**

1. Xu Z, Geng J, Zhang S, Zhang K, Yang L, Li J, et al. A mobile-based intervention for dietary behavior and physical activity change in individuals at high risk for type 2 diabetes mellitus: Randomized controlled trial. JMIR Mhealth Uhealth. 2020;8(11):e19869. doi: 10.2196/19869.
2. Harrington RA, Scarborough P, Hodgkins C, Raats MM, Cowburn G, Dean M, Doherty A, Foster C, Juszczak E, Ni Mhurchu C, Winstone N, Shepherd R, Timotijevic L, Rayner M. A pilot randomized controlled trial of a digital intervention aimed at improving food purchasing behavior: The front-of-pack food labels impact on consumer choice study. JMIR Form Res; 2019 Apr 8;3(2):e9910. doi: 10.1002/central/CN-02549982 1. PMID:30958277
3. Aalbers T, Qin L, Baars MA, Lange A de, Kessels RP, Rikkert MGO. Changing behavioral lifestyle risk factors related to cognitive decline in later life using a self-motivated eHealth intervention in Dutch adults. Journal of Medical Internet Research; 2016 Jun;18(6). PMID:27317506
4. Brown RCC, Jegatheesan DK, Conley MM, Mayr HL, Kelly JT, Webb L, Barnett A, Staudacher HM, Burton NW, Isbel NM, Macdonald GA, Campbell KL, Coombes JS, Keating SE, Hickman IJ. Utilizing technology for diet and exercise change in complex chronic conditions across diverse environments (U-DECIDE): Protocol for a randomized controlled trial. JMIR Res Protoc; 2022 Jul 28;11(7):e37556. doi: 10.1002/central/CN-02238906. PMID:35900834
5. Adu MD, Malabu UH, Malau-Aduli AEO, Malau-Aduli BS. The development of My Care Hub mobile-phone app to support self-management in Australians with type 1 or type 2 diabetes. Sci Rep; 2020 Jan 8;10(1):7. doi: 10.1038/s41598-019-56411-0
6. Ambwani S, Roche MJ, Minnick AM, Pincus AL. Negative affect, interpersonal perception, and binge eating behavior: An experience sampling study. International Journal of Eating Disorders; 2015 Sep;48(6):715–726. doi: 10.1002/eat.22410
7. Poster Session 2. Eur J Prev Cardiol. 2019;26(1_suppl):S71-S127. doi: 10.1177/2047487319860053
8. Annesi JJ. Contrasting personal characteristics and psychosocial correlates of exercise and eating behavior changes in women successful vs. unsuccessful with weight loss and maintenance. Applied Psychology: Health & Well-Being; 2020 Nov;12(3):703–723. doi: 10.1111/aphw.12203
9. Annesi JJ. Predicting 2-year weight loss through temporally specific earlier losses, relevant behaviors, and their psychological correlates: Implications for behavioral treatment architectures. Scandinavian Journal of Psychology; 2020 Oct;61(5):625–633. doi: 10.1111/sjop.12619
10. APSAD 2022 Conference, 9-12 October 2022, Darwin, Australia. Drug Alcohol Rev. 2022;41(S3):S3-S154. doi: 10.1111/dar.13537
11. Arigo D, Jake-Schoffman DE, Wolin K, Beckjord E, Hekler EB, Pagoto SL. The history and future of digital health in the field of behavioral medicine. Journal of Behavioral Medicine; 2019 Feb;42(1):67–83. doi: 10.1007/s10865-018-9966-z
12. Arps ER, Friesen MD, Overall NC. Promoting youth mental health via text-messages: A New Zealand feasibility study. Applied Psychology: Health & Well-Being; 2018 Nov;10(3):457–480. doi: 10.1111/aphw.12143
13. Ashton LM, Morgan PJ, Hutchesson MJ, Rollo ME, Collins CE. Feasibility and preliminary efficacy of the ‘HEYMAN’ healthy lifestyle program for young men: A pilot randomised controlled trial. Nutr J; 2017 Dec;16(1):2. doi: 10.1186/s12937-017-0227-8
14. Azar KMJ, Lesser LI, Laing BY, Stephens J, Aurora MS, Burke LE, Palaniappan LP. Mobile applications for weight management theory-based content analysis. Am J Prev Med; 2013 Nov;45(5):583–589. doi: 10.1016/j.amepre.2013.07.005
15. Azelton KR, Crowley AP, Vence N, Underwood K, Morris G, Kelly J, Landry MJ. Digital health coaching for type 2 diabetes: Randomized controlled trial of healthy at home. Front Digit Health; 2021 Nov 25;3:764735. doi: 10.3389/fdgth.2021.764735
16. Bamford B, Barras C, Sly R, Stiles-Shields C, Touyz S, Grange D, Hay P, Crosby R, Lacey H. Eating disorder symptoms and quality of life: Where should clinicians place their focus in severe and enduring anorexia nervosa? International Journal of Eating Disorders; 2015 Jan;48(1):133–138. doi: 10.1002/eat.22327
17. Barnwell PV, Fedorenko EJ, Contrada RJ. Healthy or not? The impact of conflicting health-related information on attentional resources. Journal of Behavioral Medicine; 2022 Apr;45(2):306–317. doi: 10.1007/s10865-021-00256-4
18. Beecher CC, Van Pay CK. Small Talk: A community research collaboration to increase parental provision of language to children. Child & Youth Care Forum; 2021 Feb;50(1):13–38. doi: 10.1007/s10566-019-09507-7
19. Berezowska A, Fischer ARH, Trijp HCM van. The moderating effect of motivation on health-related decision-making. Psychology & Health; 2017 Jun;32(6):665–685. doi: 10.1080/08870446.2017.1293055
20. Berking M, Eichler E, Naumann E, Svaldi J. The efficacy of a transdiagnostic emotion regulation skills training in the treatment of binge-eating disorder—Results from a randomized controlled trial. British Journal of Clinical Psychology; 2022 Nov;61(4):998–1018. doi: 10.1111/bjc.12371
21. Bertera ElizabethM. Storytelling slide shows to improve diabetes and high blood pressure knowledge and self-efficacy: Three-year results among community dwelling older African americans. Educational Gerontology; 2014 Nov;40(11):785–800. doi: 10.1080/03601277.2014.894381
22. Boedt T, Dancet E, De Neubourg D, Vereeck S, van der gucht K, Calster B, Spiessens C, Lie Fong S, Matthys C. A blended preconception lifestyle programme for couples undergoing IVF: Lessons learned from a multicentre randomized controlled trial. Human Reproduction Open; 2023 Sep 29;2023. doi: 10.1093/hropen/hoad036
23. Borrelli B, Henshaw M, Endrighi R, Adams WG, Heeren T, Rosen RK, Bock B, Werntz S. An interactive parent-targeted text messaging intervention to improve oral health in children attending urban pediatric clinics: Feasibility randomized controlled trial. JMIR Mhealth Uhealth; 2019 Nov 11;7(11):e14247. doi: 10.2196/14247
24. Boucher EM, Ward HE, Mounts AC, Parks AC. Engagement in digital mental health interventions: Can monetary incentives help? Front Psychol; 2021 Nov 18;12:746324. doi: 10.3389/fpsyg.2021.746324
25. Brewer LC, Jenkins S, Hayes SN, Kumbamu A, Jones C, Burke LE, Cooper LA, Patten CA. Community-based, cluster-randomized pilot trial of a cardiovascular mobile health intervention: Preliminary findings of the FAITH! trial. Circulation; 2022 Jul 19;146(3):175–190. doi: 10.1161/CIRCULATIONAHA.122.059046
26. Brown AF, Welsh T, Panton LB, Moffatt RJ, Ormsbee MJ. Higher-protein intake improves body composition index in female collegiate dancers. Applied Physiology, Nutrition & Metabolism; 2020 May;45(5):547–554. doi: 10.1139/apnm-2019-0517
27. Buchan K, Morgan HM. Using the Onitor® Track for weight loss: A mixed methods study among overweight and obese women. Health Inform J Thousand; 2020 Sep;26(3):1841–1865. doi: 10.1177/1460458219890790
28. Buchholz SW, Ingram D, Wilbur J, Fogg L, Sandi G, Moss A, Ocampo EV. Bilingual Text4Walking Food service employee intervention pilot study. JMIR Mhealth Uhealth; 2016 Jun;4(2):361–372. doi: 10.2196/mhealth.5328
29. Butts SA, Kayukwa A, Langlie J, Rodriguez VJ, Alcaide ML, Chitalu N, Weiss SM, Jones DL. HIV knowledge and risk among Zambian adolescent and younger adolescent girls: Challenges and solutions. Sex Education; 2018 Jan;18(1):1–13. doi: 10.1080/14681811.2017.1370368
30. Cadmus-Bertram L, Nelson S, Hartman S, Patterson R, Parker B, Pierce J. Randomized trial of a phone- and web-based weight loss program for women at elevated breast cancer risk: the HELP study. Journal of Behavioral Medicine; 2016 Aug;39(4):551–559. doi: 10.1007/s10865-016-9735-9
31. Chwyl C, Wright N, Turner-McGrievy GM, Butryn ML, Forman EM. Remotely delivered behavioral weight loss intervention using an Ad libitum plant-based diet: Pilot acceptability, feasibility, and preliminary results. JMIR Form Res; 2022 Jun;6(6):e37414. doi: 10.2196/37414
32. Conroy DE, West AB, Brunke-Reese D, Thomaz E, Streeper NM. Just-in-time adaptive intervention to promote fluid consumption in patients with kidney stones. Health Psychol; 2020 Dec;39(12):1062–1069. doi: 10.1037/hea0001032
33. Cooper M, Duncan B, Golden S, Toth K. Systematic client feedback in therapy for children with psychological difficulties: Pilot cluster randomised controlled trial. Counselling Psychology Quarterly; 2021 Mar;34(1):21–36. doi: 10.1080/09515070.2019.1647142
34. Crawford DA, Heinrich KM, Drake NB, DeBlauw J, Carper MJ. Heart rate variability mediates motivation and fatigue throughout a high-intensity exercise program. Applied Physiology, Nutrition & Metabolism; 2020 Feb;45(2):193–202. doi: 10.1139/apnm-2019-0123
35. Crimarco A, Turner-McGrievy GM, Wirth MD. The effects of meal-timing on self-rated hunger and dietary inflammatory potential among a sample of college students. Journal of American College Health; 2019 Jun 5;67(4):328–337. doi: 10.1080/07448481.2018.1481074
36. Cross A. Managing anxiety and stress in hypertension: Development of heart rate variability biofeedback with psychological support. Journal of Hypertension; 2023 Jun;41(Suppl 3):e175. doi: 10.1097/01.hjh.0000940680.38181.91
37. Dai YG, Thomas RP, Brennan L, Helt MS, Barton ML, Dumont-Mathieu T, Fein DA. Development and acceptability of a new program for caregivers of children with autism spectrum disorder: Online parent training in early behavioral intervention. Journal of Autism & Developmental Disorders; 2021 Nov;51(11):4166–4185. doi: 10.1007/s10803-020-04863-z
38. Deruelle P, Lelorain S, Deghilage S, Couturier E, Guilbert E, Berveiller P, Senat MV, Vayssiere C, Sentilhes L, Perrotin F, Gallot D, Chauleur C, Sananes N, Roth E, Luton D, Caputo M, Lorio E, Chatelet C, Couster J, Timbely O, Doret-Dion M, Duhamel A, Pigeyre M. Rationale and design of ePPOP-ID: A multicenter randomized controlled trial using an electronic-personalized program for obesity in pregnancy to improve delivery. BMC Pregnancy Childbirth; 2020 Oct 7;20(1):602. doi: 10.1186/s12884-020-03288-x
39. Dhinagaran DA, Sathish T, Soong A, Theng Y-L, Best J, Car LT. Conversational agent for healthy lifestyle behavior change: Web-based feasibility study. JMIR Form Res; 2021 Dec;5(12):e27956. doi: 10.2196/27956
40. Dillon K, Rollo S, Prapavessis H. A combined health action process approach and mHealth intervention to reduce sedentary behaviour in university students—A randomized controlled trial. Psychology & Health; 2022 Jun;37(6):692–711. doi: 10.1080/08870446.2021.1900574
41. dos Reis AC, Vidal CL, de Souza Caetano KA, Dias HD. Use of recorded poetic audios to manage levels of anxiety and sleep disorders. Journal of Religion & Health; 2020 Jun;59(3):1626–1634. doi: 10.1007/s10943-019-00947-y
42. Ebrahimian S, Zink J, Yang C-H, Yu Q, Imm K, Nicolo M, Dunton GF, Belcher BR. Momentary intentions and perceived behavioral control are within-person predictors of sedentary leisure time: Preliminary findings from an ecological momentary assessment study in adolescents. Journal of Behavioral Medicine; 2022 Jun;45(3):391–403. doi: 10.1007/s10865-022-00309-2
43. Eisenhauer CM, Brito F, Kupzyk K, Yoder A, Almeida F, Beller RJ, Miller J, Hageman PA. Mobile health assisted self-monitoring is acceptable for supporting weight loss in rural men: A pragmatic randomized controlled feasibility trial. BMC Public Health; 2021 Aug 18;21(1):1568. doi: 10.1186/s12889-021-11618-7
44. European Obesity Summit (EOS) —Joint Congress of EASOand IFSO-EC, Gothenburg, Sweden, June 1–4, 2016: Abstracts. Obes Facts; 2016;9(1):1–376. doi: 10.1159/000446744
45. Faller H, Hass HG, Engehausen D, Reuss-Borst M, Wöckel A. Supportive care needs and quality of life in patients with breast and gynecological cancer attending inpatient rehabilitation. A prospective study. Acta Oncologica; 2019 Apr;58(4):417–424. doi: 10.1080/0284186X.2018.1543947
46. Fang CM, McMahon K, Miller ML, Rosenthal MZ. A pilot study investigating the efficacy of brief, phone-based, behavioral interventions for burnout in graduate students. Journal of Clinical Psychology; 2021 Dec;77(12):2725–2745. doi: 10.1002/jclp.23245
47. Félix S, Ramalho S, Ribeiro E, Pinheiro J, de Lourdes M, Gonçalves S, Conceição E. Experiences of parent–adolescent dyads regarding a Facebook-based intervention to improve overweight/obesity treatment in adolescents: A qualitative study. Applied Psychology: Health & Well-Being; 2022 Feb;14(1):122–139. doi: 10.1111/aphw.12294
48. Fischer X, Donath L, Zahner L, Faude O, Gerber M. Exploring psychosocial mediators of remote physical activity counselling: a secondary analysis of data from a 1-year randomized control trial (Movingcall). Journal of Behavioral Medicine; 2020 Apr;43(2):271–285. doi: 10.1007/s10865-019-00112-6
49. Fitzsimmons-Craft EE, Taylor CB, Graham AK, Sadeh-Sharvit S, Balantekin KN, Eichen DM, Monterubio GE, Goel NJ, Flatt RE, Karam AM, Firebaugh M-L, Jacobi C, Jo B, Trockel MT, Wilfley DE. Effectiveness of a digital cognitive behavior therapy–guided self-help intervention for eating disorders in college women. JAMA Netw Open; 2020 Aug 31;3(8):e2015633. PMID:32865576
50. Fjeldsoe BS, Miller YD, Prosser SJ, Marshall AL. How does MobileMums work? Mediators of a physical activity intervention. Psychology & Health; 2020 Aug;35(8):968–983. doi: 10.1080/08870446.2019.1687698
51. Forbes CC, Blanchard CM, Mummery WK, Courneya KS. A pilot study on the motivational effects of an internet-delivered physical activity behaviour change programme in Nova Scotian cancer survivors. Psychology & Health; 2017 Feb;32(2):234–252. doi: 10.1080/08870446.2016.1260725
52. Forman EM, Kerrigan SG, Butryn ML, Juarascio AS, Manasse SM, Ontañón S, Dallal DH, Crochiere RJ, Moskow D. Can the artificial intelligence technique of reinforcement learning use continuously-monitored digital data to optimize treatment for weight loss? Journal of Behavioral Medicine; 2019 Apr;42(2):276–290. doi: 10.1007/s10865-018-9964-1
53. Forman EM, Manasse SM, Dallal DH, Crochiere RebeccaJ, Loyka CM, Butryn ML, Juarascio AS, Houben K. Computerized neurocognitive training for improving dietary health and facilitating weight loss. Journal of Behavioral Medicine; 2019 Dec;42(6):1029–1040. doi: 10.1007/s10865-019-00024-5
54. Frie K, Hartmann-Boyce J, Pilbeam C, Jebb S, Aveyard P. Analysing self-regulatory behaviours in response to daily weighing: A think-aloud study with follow-up interviews. Psychology & Health; 2020 Jan;35(1):16–35. doi: 10.1080/08870446.2019.1626394
55. Galla BM, Choukas-Bradley S, Fiore HM, Esposito MV. Values-alignment messaging boosts adolescents’ motivation to control social media use. Child Development; 2021 Oct 9;92(5):1717–1734. doi: 10.1111/cdev.13553
56. Galy O, Yacef K, Caillaud C. Improving pacific adolescents’ physical activity toward international recommendations: Exploratory study of a digital education app coupled with activity trackers. JMIR Mhealth Uhealth. 2019 Dec 11;7(12):e14854. PMID:31825319
57. Geramita EM, Dabbs AJD, DiMartini AF, Pilewski JM, Switzer GE, Posluszny DM, Myaskovsky L, Dew MA. Impact of a mobile health intervention on long-term nonadherence after lung transplantation: follow-up after a randomized controlled trial. Transplantation; 2020 Mar;104(3):640–651. doi: 10.1097/TP.0000000000002872
58. Gipson CS, Chilton JM, Dickerson SS, Alfred D, Haas BK. Effects of a sleep hygiene text message intervention on sleep in college students. Journal of American College Health; 2019 Jan;67(1):32–41. doi: 10.1080/07448481.2018.1462816
59. Goldman J. Can MOOCs enhance sexuality education? Sex Education; 2016 Sep;16(5):487–502. doi: 10.1080/14681811.2015.1112261
60. Goldstein SP, Hoover A, Evans EW, Thomas JG. Combining ecological momentary assessment, wrist-based eating detection, and dietary assessment to characterize dietary lapse: A multi-method study protocol. Digit Health; 2021 Jan;7:2055207620988212. doi: 10.1177/2055207620988212
61. Gourlan M, Sarrazin P, Trouilloud D. Motivational interviewing as a way to promote physical activity in obese adolescents: A randomised-controlled trial using self-determination theory as an explanatory framework. Psychology & Health; 2013 Nov;28(11):1265–1286. doi: 10.1080/08870446.2013.800518
62. Greco G, Poli L, Clemente FM, Francesco F, Cataldi S. The effectiveness of new digital technologies in increasing physical activity levels and promoting active and healthy ageing: A narrative review. Health Soc Care Community; 2023 Feb 8;1–11. doi: 10.1155/2023/2803620
63. Griffin JB, Struempler B, Funderburk K, Parmer SM, Tran C, Wadsworth DD. My quest, an intervention using text messaging to improve dietary and physical activity behaviors and promote weight loss in low-income women. J Nutr Educ Behav; 2018 Jan;50(1):11–18. doi: 10.1016/j.jneb.2017.09.007
64. Hall KS, Morey MC, Bosworth HB, Beckham JC, Pebole MM, Sloane R, Pieper CF. Pilot randomized controlled trial of exercise training for older veterans with PTSD. Journal of Behavioral Medicine; 2020 Aug;43(4):648–659. doi: 10.1007/s10865-019-00073-w
65. Harris L, Hankey C, Jones N, Murray H, Pert C, Tobin J, Boyle S, Shearer R, Melville CA. Process evaluation of a cluster-randomised controlled trial of multi-component weight management programme in adults with intellectual disabilities and obesity. Journal of Intellectual Disability Research; 2019 Jan;63(1):49–63. doi: 10.1111/jir.12563
66. Hattar A, Pal S, Hagger MS. Predicting physical activity-related outcomes in overweight and obese adults: A health action process approach. Applied Psychology: Health & Well-Being; 2016 Mar;8(1):127–151. doi: 10.1111/aphw.12065
67. Hemphill RachelC, Stephens MP, Rook KarenS, Franks MelissaM, Salem JamesK. Older adults’ beliefs about the timeline of type 2 diabetes and adherence to dietary regimens. Psychology & Health; 2013 Feb;28(2):139–153. doi: 10.1080/08870446.2012.685740
68. Herodotou C, Heiser S, Rienties B. Implementing randomised control trials in open and distance learning: A feasibility study. Open Learning; 2017 Jun;32(2):147–162. doi: 10.1080/02680513.2017.1316188
69. Heslehurst N, Cullen E, Flynn AC, Briggs C, Smart L, Rankin J, McColl E, Sniehotta FF, McParlin C. Maternal obesity and patterns in postnatal diet, physical activity and weight among a highly deprived population in the uk: the glowing pilot trial. Nutrients; 2023 Aug 30;15(17):3805. doi: 10.3390/nu15173805
70. Hildebrandt T, Michaelides A, Mackinnon D, Greif R, DeBar L, Sysko R. Randomized controlled trial comparing smartphone assisted versus traditional guided self-help for adults with binge eating. Int J Eating Disord; 2017 Nov;50(11):1313–1322. doi: 10.1002/eat.22781
71. Hjorth P, Davidsen AS, Kilian R, Jensen SOW, Munk-Jørgensen P. Intervention to promote physical health in staff within mental health facilities and the impact on patients’ physical health. Nordic Journal of Psychiatry; 2016 Jan;70(1):62–71. doi: 10.3109/08039488.2015.1050452
72. Ho HCY, Mui MW, Wan A, Yew CW, Lam TH. Happy family kitchen movement: a cluster randomized controlled trial of a community-based family holistic health intervention in Hong Kong. Journal of Happiness Studies; 2020 Jan;21(1):15–36. doi: 10.1007/s10902-018-00071-w
73. Hu L, Illiano P, Pompeii ML, Popp CJ, Kharmats AY, Curran M, Perdomo K, Chen S, Bergman M, Segal E, Sevick MA. Challenges of conducting a remote behavioral weight loss study: Lessons learned and a practical guide. Contemp Clin Trials; 2021 Sep;108:106522. doi: 10.1016/j.cct.2021.106522
74. Jahan Y, Rahman MM, Faruque ASG, Chisti MJ, Kazawa K, Matsuyama R, Moriyama M. Awareness development and usage of mobile health technology among individuals with hypertension in a rural community of bangladesh: Randomized controlled trial. J Med Internet Res; 2020 Dec 7;22(12):e19137. doi: 10.2196/19137
75. Jamruk K. The weight game: fighting childhood obesity with childhood video technology. Journal of Legal Medicine; 2017 Jan;37(1/2):175–194. doi: 10.1080/01947648.2017.1303409
76. Jia SS, Allman-Farinelli M, Roy R, Phongsavan P, Hyun K, Gibson AA, Partridge SR. Using mobile ecological momentary assessment to understand consumption and context around online food delivery use: pilot feasibility and acceptability study. JMIR Mhealth Uhealth; 2023;11:e49135. doi: 10.2196/49135
77. Jiang H, Li M, Wen LM, Hu Q, Yang D, He G, Baur LA, Dibley MJ, Qian X. Effect of short message service on infant feeding practice findings from a community-based study in shanghai, China. JAMA Pediatr; 2014 May;168(5):471–478. doi: 10.1001/jamapediatrics.2014.58
78. Jones C, Gibbons M, Magsamen-Conrad K, Ulanday KT, Watterson J, Oakley-Girvan I, Houghton LC, Gokal K. Mother’s health and well-being matters: Is a mediated social cohesion public health intervention feasible? Am J Health Promot; 2022 Mar;36(3):410–420. doi: 10.1177/08901171211055317
79. Juarascio AS, Srivastava P, Presseller EK, Lin M, Patarinski GG, Manasse SM, Forman EM. Using continuous glucose monitoring to detect and intervene on dietary restriction in individuals with binge eating: The sensesupport withdrawal design study. JMIR Form Res; 2022;6:e38479. doi: 10.2196/38479
80. Kalantarzadeh E, Radahmadi M, Reisi P. The impact of different dark chocolate dietary patterns on synaptic potency and plasticity in the hippocampal CA1 area of the rats under chronic isolation stress. Nutritional Neuroscience; 2023 Aug;26(8):756–765. doi: 10.1080/1028415X.2022.2088946
81. Kauffman BY, Gallagher MW, Viana AG, Schmidt NB, Zvolensky MJ. Computer-delivered intervention for individuals with obesity and elevated anxiety sensitivity: feasibility, acceptability, and initial test. Cognitive Behaviour Therapy; 2022 Sep;51(5):353–370. doi: 10.1080/16506073.2021.2018487
82. Keadle SK, Meuter L, Phelan S, Phillips SM. Charity-based incentives motivate young adult cancer survivors to increase physical activity: A pilot randomized clinical trial. Journal of Behavioral Medicine; 2021 Oct;44(5):682–693. doi: 10.1007/s10865-021-00218-w
83. Kelchen R, Goldrick-Rab S. Accelerating college knowledge: A fiscal analysis of a targeted early commitment pell grant program. Journal of Higher Education; 2015 Apr 3;86(2):199–231. doi: 10.1353/jhe.2015.0007
84. Kempf K, Dubois C, Arnold M, Amelung V, Leppert N, Altin S, Vomhof M, Icks A, Martin S. Effectiveness of the telemedical lifestyle intervention program telipro for improvement of hba1c in type 2 diabetes: A randomized-controlled trial in a real-life setting. Nutrients; 2023 Sep 12;15(18):3954. doi: 10.3390/nu15183954
85. Keshen A, Helson T, Town J, Warren K. Self-efficacy as a predictor of treatment outcome in an outpatient eating disorder program. Eating Disorders; 2017 Oct;25(5):406–419. doi: 10.1080/10640266.2017.1324073
86. Kim J-W, Ryu B, Cho S, Heo E, Kim Y, Lee J, Jung SY, Yoo S. Impact of personal health records and wearables on health outcomes and patient response: three-arm randomized controlled trial. JMIR Mhealth Uhealth; 2019 Jan 4;7(1):e12070. doi: 10.2196/12070
87. Kim M, Yang J, Ahn W-Y, Choi HJ. Machine learning analysis to identify digital behavioral phenotypes for engagement and health outcome efficacy of an mhealth intervention for obesity: randomized controlled trial. J Med Internet Res; 2021 Jun 24;23(6):e27218. doi: 10.2196/27218
88. Kingsnorth AP, Whelan ME, Orme MW, Routen AC, Sherar LB, Esliger DW. Resistance to data loss from the Freestyle Libre: Impact on glucose variability indices and recommendations for data analysis. Applied Physiology, Nutrition & Metabolism; 2021 Feb;46(2):148–154. doi: 10.1139/apnm-2020-0386
89. Kolaas K, Axelsson E, Hedman-Lagerlöf E, Berman AH. Healthy lifestyle promotion via digital self-help for mental health patients in primary care: A pilot study including an embedded randomized recruitment trial. Prim Health Care Res Dev. 2023 Sep 20;24:e56. PMID:37728142
90. Kristoffersen M, Johnson C, Atkinson MJ. Feasibility and acceptability of video-based microinterventions for eating disorder prevention among adolescents in secondary schools. International Journal of Eating Disorders; 2022 Nov;55(11):1496–1505. doi: 10.1002/eat.23781
91. Kwag KH, Kim Y, Lee YK, Lee HR, Lee JY, Lee J, Kim J, Kim Y, Treasure J. Feasibility and acceptability of a digital tele-guided intervention targeting components of the addictive appetite model for bulimia nervosa and binge-eating disorder in Korea. International Journal of Eating Disorders; 2022 Jul;55(7):977–982. doi: 10.1002/eat.23754
92. Kwan BM, Bryan AD, Sheeran P. The dynamics of success and failure: how post-behaviour evaluations relate to subsequent exercise intentions and behaviour. Psychology & Health; 2018 Jul;33(7):888–905. doi: 10.1080/08870446.2018.1429612
93. Kwasnicka D, Dombrowski SU, White M, Sniehotta FF. N-of-1 study of weight loss maintenance assessing predictors of physical activity, adherence to weight loss plan and weight change. Psychology & Health; 2017 Jun;32(6):686–708. doi: 10.1080/08870446.2017.1293057
94. La Scala Teixeira CV, Caranti DA, Oyama LM, Padovani R da C, Cuesta MGS, Moraes A dos S, Cerrone LA, Affonso LHL, Gil S dos S, dos Santos RVT, Gomes RJ. Effects of functional training and 2 interdisciplinary interventions on maximal oxygen uptake and weight loss of women with obesity: A randomized clinical trial. Applied Physiology, Nutrition & Metabolism; 2020 Jul;45(7):777–783. doi: 10.1139/apnm-2019-0766
95. Labhart F, Tarsetti F, Bornet O, Santani D, Truong J, Landolt S, Gatica-Perez D, Kuntsche E. Capturing drinking and nightlife behaviours and their social and physical context with a smartphone application—investigation of users’ experience and reactivity. Addiction Research & Theory; 2020 Feb;28(1):62–75. doi: 10.1080/16066359.2019.1584292
96. Lappalainen R, Sairanen E, Jarvela E, Rantala S, Korpela R, Puttonen S, Kujala UM, Myllymaki T, Peuhkuri K, Mattila E, Kaipainen K, Ahtinen A, Karhunen L, Pihlajamaki J, Jarnefelt H, Laitinen J, Kutinlahti E, Saarelma O, Ermes M, Kolehmainen M. The effectiveness and applicability of different lifestyle interventions for enhancing wellbeing: the study design for a randomized controlled trial for persons with metabolic syndrome risk factors and psychological distress. BMC Public Health; 2014 Apr 4;14:310. doi: 10.1186/1471-2458-14-310
97. Larsson G, Lundell E, Svensén S, Nilsson S. Interrelationship of emotional stability, hassles, uplifts, coping and stress-related symptoms in Swedish female and male military veterans. Scandinavian Journal of Psychology; 2021 Apr;62(2):217–226. doi: 10.1111/sjop.12701
98. Lawson DW, Stolwyk RJ, Ponsford JL, Wong D. Evaluating telehealth delivery of a compensatory memory rehabilitation programme following stroke: A single-case experimental design. Neuropsychological Rehabilitation; 2022 Jul;32(6):897–921. doi: 10.1080/09602011.2020.1843500
99. Lee M-K, Lee DY, Ahn H-Y, Park C-Y. A novel user utility score for diabetes management using tailored mobile coaching: Secondary analysis of a randomized controlled trial. JMIR Mhealth Uhealth. 2021 Feb 24;9(2):e17573. PMID:33625363
100. Leonard JA, Lydon-Staley DM, Sharp SDS, Liu HZ, Park AT, Bassett DS, Duckworth AL, Mackey AP. Daily fluctuations in young children’s persistence. Child Development; 2022 Mar;93(2):e222–e236. doi: 10.1111/cdev.13717
101. Leone LA, Tripicchio GL, Haynes-Maslow L, McGuirt J, Smith JSG, Armstrong-Brown J, Gizlice Z, Ammerman A. Cluster randomized controlled trial of a mobile market intervention to increase fruit and vegetable intake among adults in lower-income communities in North Carolina. Int J Behav Nutr Phys Act; 2018 Jan 5;15:2. doi: 10.1186/s12966-017-0637-1
102. Leonidas C, Nazar BP, Munguía L, Santos MA. How do we target the factors that maintain anorexia nervosa? A behaviour change taxonomical analysis. International Review of Psychiatry; 2019 Jun;31(4):403–410. doi: 10.1080/09540261.2019.1624509
103. Levin ME, Krafft J, Davis CH, Twohig MP. Evaluating the effects of guided coaching calls on engagement and outcomes for online acceptance and commitment therapy. Cognitive Behaviour Therapy; 2021 Sep;50(5):395–408. doi: 10.1080/16506073.2020.1846609
104. Levin MichaelE, Pistorello J, Seeley JohnR, Hayes StevenC. Feasibility of a prototype web-based acceptance and commitment therapy prevention program for college students. Journal of American College Health; 2014 Jan;62(1):20–30. doi: 10.1080/07448481.2013.843533
105. Levy E, Warner LM, Fleig L, Kaufman MR, Deschepper R, Gidron Y. The effects of psychological inoculation on condom use tendencies and barriers; A randomized controlled trial. Psychology & Health; 2021 May;36(5):575–592. doi: 10.1080/08870446.2020.1775832
106. Lopez-Pentecost M, Perkin S, Freylersythe S, Rossi P, Rolle LD, St. George SM, Crane TE. Feasibility and acceptability of a text message intervention to promote adherence to nutrition and physical activity guidelines in a predominantly hispanic sample of cancer survivors and their informal caregivers: results from a pilot intervention trial. Nutrients. 2023 Nov 16;15(22):4799. doi: 10.3390/nu15224799
107. Mack I, Reiband N, Etges C, Eichhorn S, Schaeffeler N, Zurstiege G, Gawrilow C, Weimer K, Peeraully R, Teufel M, Blumenstock G, Giel KE, Junne F, Zipfel S. The kids obesity prevention program: cluster randomized controlled trial to evaluate a serious game for the prevention and treatment of childhood obesity. J Med Internet Res; 2020 Apr 24;22(4):e15725. doi: 10.2196/15725
108. Mackey E, Schweitzer A, Hurtado ME, Hathway J, DiPietro L, Lei KY, Klein CJ. The feasibility of an e-mail–delivered intervention to improve nutrition and physical activity behaviors in African American college students. Journal of American College Health; 2015 Mar 2;63(2):109–117. doi: 10.1080/07448481.2014.990971
109. Mahapatra S, Parker ME, Dave N, Zobrist SC, Shajie Arul D, King A, Betigeri A, Sachdeva R. Micronutrient-fortified rice improves haemoglobin, anaemia prevalence and cognitive performance among schoolchildren in Gujarat, India: a case-control study. International Journal of Food Sciences & Nutrition; 2021 Aug;72(5):690–703. doi: 10.1080/09637486.2020.1855126
110. Malins S, Biswas S, Patel S, Levene J, Moghaddam N, Morriss R. Preventing relapse with personalized smart-messaging after cognitive behavioural therapy: A proof-of-concept evaluation. British Journal of Clinical Psychology; 2020 Jun;59(2):241–259. doi: 10.1111/bjc.12244
111. Manuel JK, Lum PJ, Hengl NS, Sorensen JL. Smoking cessation interventions with female smokers living with HIV/AIDS: A randomized pilot study of motivational interviewing. AIDS Care; 2013 Jul;25(7):820–827. doi: 10.1080/09540121.2012.733331
112. Marler JD, Fujii CA, Utley DS, Tesfamariam LJ, Galanko JA, Patrick H. Initial assessment of a comprehensive digital smoking cessation program that incorporates a mobile app, breath sensor, and coaching: cohort study. JMIR Mhealth Uhealth; 2019 Feb 4;7(2):e12609. doi: 10.2196/12609
113. Mattila E, Hansen S, Bundgaard L, Ramsey L, Dunning A, Silva MN, Harjumaa M, Ermes M, Marques MM, Matos M, Larsen SC, Encantado J, Santos I, Horgan G, O’Driscoll R, Turicchi J, Duarte C, Palmeira AL, Stubbs RJ, Heitmann BL, Lahteenmaki L. Users’ experiences with the nohow web-based toolkit with weight and activity tracking in weight loss maintenance: long-term randomized controlled trial. J Med Internet Res; 2022 Jan 10;24(1):e29302. doi: 10.2196/29302
114. Mauch CE, Wycherley TP, Laws RA, Johnson BJ, Bell LK, Golley RK. Mobile apps to support healthy family food provision: systematic assessment of popular, commercially available apps. JMIR Mhealth Uhealth; 2018 Dec;6(12):e11867. doi: 10.2196/11867
115. McAleese D, Linardakis M, Papadaki A. Quality and presence of behaviour change techniques in mobile apps for the mediterranean diet: a content analysis of android google play and apple app store apps. Nutrients; 2022 Mar;14(6):1290. doi: 10.3390/nu14061290
116. McArthur BA, Racine N, McDonald S, Tough S, Madigan S. Child and family factors associated with child mental health and well-being during COVID-19. European Child & Adolescent Psychiatry; 2023 Feb;32(2):223–233. doi: 10.1007/s00787-021-01849-9
117. McGarry E, Vernon T, Baktha A. brief report: a pilot online pivotal response treatment training program for parents of toddlers with autism spectrum disorder. Journal of Autism & Developmental Disorders; 2020 Sep;50(9):3424–3431. doi: 10.1007/s10803-019-04100-2
118. McMahon J, Thompson DR, Brazil K, Ski CF. An eHealth intervention (ManGuard) to reduce cardiovascular disease risk in male taxi drivers: protocol for a feasibility randomised controlled trial. Pilot Feasibility Stud; 2022 Sep 14;8(1):209. doi: 10.1186/s40814-022-01163-4
119. Meier C, Lemmer E. Parents as consumers: a case study of parent satisfaction with the quality of schooling. Educational Review; 2019 Sep;71(5):617–630. doi: 10.1080/00131911.2018.1465395
120. Melchart D, Doerfler W, Eustachi A, Wellenhofer-Li Y, Weidenhammer W. The talent study: a multicentre randomized controlled trial assessing the impact of a “tailored lifestyle self-management intervention” (talent) on weight reduction. BMC Obes; 2015;2:38. PMID:26435840
121. Mendez IM, Pories ML, Cordova L, Malki A, Wiggins MF, Lee JGL. A pilot project to increase health literacy among youth from seasonal farmworker families in rural eastern North Carolina: a qualitative exploration of implementation and impact. Journal of the Medical Library Association; 2019 Apr;107(2):179–186. doi: 10.5195/jmla.2019.560
122. Middleton KathrynR, Perri MichaelG. A randomized trial investigating the effect of a brief lifestyle intervention on freshman-year weight gain. Journal of American College Health; 2014 Mar 2;62(2):101–109. doi: 10.1080/07448481.2013.849259
123. Miller HN, Voils C, Cronin KA, Jeanes E, Hawley J, Porter LS, Adler RR, Sharp W, Pabich S, Gavin KL, Lewis MA, Johnson HM, Yancy WS, Gray KE, Shaw RJ. A method to deliver automated and tailored intervention content: 24-month clinical trial. JMIR Form Res; 2022 Sep;6(9):e38262. doi: 10.2196/38262
124. Mimiaga MJ, Pantalone DW, Biello KB, Hughto JMW, Frank J, O’Cleirigh C, Reisner SL, Restar A, Mayer KH, Safren SA. An initial randomized controlled trial of behavioral activation for treatment of concurrent crystal methamphetamine dependence and sexual risk for HIV acquisition among men who have sex with men. AIDS Care; 2019 Sep;31(9):1083–1095. doi: 10.1080/09540121.2019.1595518
125. Miu AS, Vo HT, Palka JM, Glowacki CR, Robinson RJ. Teletherapy with serious mental illness populations during COVID-19: Telehealth conversion and engagement. Counselling Psychology Quarterly; 2021 Dec;34(3/4):704–721. doi: 10.1080/09515070.2020.1791800
126. Mozafarinia M, Rajabiyazdi F, Brouillette M-J, Fellows LK, Knäuper B, Mayo NE. Effectiveness of a personalized health profile on specificity of self-management goals among people living with HIV in Canada: findings from a blinded pragmatic randomized controlled trial. Quality of Life Research; 2023 Feb;32(2):413–424. doi: 10.1007/s11136-022-03245-5
127. Muldrew DHL, Fee A, Coates V. Impact of the COVID-19 pandemic on family carers in the community: A scoping review. Health & Social Care in the Community. 2022;30(4):1275–1285. doi: 10.1111/hsc.13677
128. Muradyan A, Macheiner T, Mardiyan M, Sekoyan E, Sargsyan K. The evaluation of biomarkers of physical activity on stress resistance and wellness. Applied Psychophysiology & Biofeedback; 2022 Jun;47(2):121–129. doi: 10.1007/s10484-022-09538-2
129. Najnin N, Leder K, Qadri F, Forbes A, Unicomb L, Winch PJ, Ram PK, Leontsini E, Nizame FA, Arman S, Begum F, Biswas SK, Clemens JD, Ali M, Cravioto A, Luby SP. Impact of adding hand-washing and water disinfection promotion to oral cholera vaccination on diarrhoea-associated hospitalization in Dhaka, Bangladesh: Evidence from a cluster randomized control trial. Int J Epidemiol; 2017 Dec;46(6):2056–2066. doi: 10.1093/ije/dyx187
130. Nichol B, Rodrigues AM, Wilson R, Haighton C. A systematic review of the effectiveness of brief health behaviour change interventions on service users accessing the third and social economy sector. Health Soc Care Community; 2023 Sep 20;1–24. doi: 10.1155/2023/2928228
131. Nuño T, Torres MR, Soto S, Sepulveda R, Aceves B, Rosales CB. Feasibility and outcomes of meta salud diabetes behavioral health intervention: a pilot study of a community health worker-administered educational intervention to prevent cardiovascular disease and its complications among hispanic patients with type-2 diabetes. IJERPH; 2023 Oct 24;20(21):6968. doi: 10.3390/ijerph20216968
132. O’Neal LJ, Perri MG, Befort C, Janicke DM, Shankar MN, Bauman V, Daniels MJ, Dhara K, Ross KM. Differential impact of telehealth extended-care programs for weight-loss maintenance in African American versus white adults. Journal of Behavioral Medicine; 2022 Aug;45(4):580–588. doi: 10.1007/s10865-022-00291-9
133. Oates J, Södersten M, Quinn S, Nygren U, Dacakis G, Kelly V, Smith G, Sand A. Gender-affirming voice training for trans women: effectiveness of training on patient-reported outcomes and listener perceptions of voice. Journal of Speech, Language & Hearing Research. American Speech-Language-Hearing Association; 2023 Nov;66(11):4206–4235. doi: 10.1044/2023_JSLHR-23-00258
134. Ori EM, Berry TR. Physical activity information seeking among emerging adults attending university. Journal of American College Health; 2022 Jan;70(1):223–231. doi: 10.1080/07448481.2020.1740230
135. Østergård OK, Fenger M, Hougaard E. Symptomatic distress and effectiveness of psychological treatments delivered at a nationwide student counseling service. Counselling Psychology Quarterly; 2019 Jun;32(2):150–168. doi: 10.1080/09515070.2017.1410696
136. Pacia C, Gunning C, McTiernan A, Holloway J. Developing the parent-coaching assessment, individualization, and response to stressors (pairs) tool for behavior analysts. Journal of Autism & Developmental Disorders; 2023 Sep;53(9):3319–3342. doi: 10.1007/s10803-022-05637-5
137. Pagoto S, Tulu B, Agu E, Waring ME, Oleski JL, Jake-Schoffman DE. Using the habit app for weight loss problem solving: development and feasibility study. JMIR Mhealth Uhealth; 2018 Jun;6(6):e145. doi: 10.2196/mhealth.9801
138. Patel ML, Cleare AE, Smith CM, Rosas LG, King AC. Detailed versus simplified dietary self-monitoring in a digital weight loss intervention among racial and ethnic minority adults: fully remote, randomized pilot study. JMIR Form Res; 2022 Dec;6(12):e42191. doi: 10.2196/42191
139. Patterson TL, Semple SJ, Abramovitz D, Harvey-Vera A, Pines HA, Verdugo S, Depp C, Moore DJ, Martinez G, Rangel MG, Strathdee SA. Impact of time perspectives on texting intervention to reduce HIV/STI transmission among female sex workers in Tijuana and Ciudad Juarez, Mexico. Journal of Behavioral Medicine; 2019 Feb;42(1):111–127. doi: 10.1007/s10865-018-9948-1
140. Piel AK, Crunchant A, Knot IE, Chalmers C, Fergus P, Mulero-Pázmány M, Wich SA. Noninvasive technologies for primate conservation in the 21st century. International Journal of Primatology; 2022 Feb;43(1):133–167. doi: 10.1007/s10764-021-00245-z
141. Popp CJ, Hu L, Kharmats AY, Curran M, Berube L, Wang C, Pompeii ML, Illiano P, St-Jules DE, Mottern M, Li H, Williams N, Schoenthaler A, Segal E, Godneva A, Thomas D, Bergman M, Schmidt AM, Sevick MA. Effect of a personalized diet to reduce postprandial glycemic response vs a low-fat diet on weight loss in adults with abnormal glucose metabolism and obesity: a randomized clinical trial. JAMA Netw Open; 2022 Sep 28;5(9):e2233760. doi: 10.1001/jamanetworkopen.2022.33760
142. Popp CJ, St-Jules DE, Hu L, Ganguzza L, Illiano P, Curran M, Li H, Schoenthaler A, Bergman M, Schmidt AM, Segal E, Godneva A, Sevick MA. The rationale and design of the personal diet study, a randomized clinical trial evaluating a personalized approach to weight loss in individuals with pre-diabetes and early-stage type 2 diabetes. Contemporary Clinical Trials; 2019 Apr;79:80–88. doi: 10.1016/j.cct.2019.03.001
143. Poppe L, Van der Mispel C, De Bourdeaudhuij I, Verloigne M, Shadid S, Crombez G. Users’ thoughts and opinions about a self-regulation-based eHealth intervention targeting physical activity and the intake of fruit and vegetables: A qualitative study. PLoS One; 2017 Dec 21;12(12):e0190020. doi: 10.1371/journal.pone.0190020
144. Ramadas A, Chan CKY, Oldenburg B, Hussien Z, Quek KF. A web-based dietary intervention for people with type 2 diabetes: development, implementation, and evaluation. International journal of behavioral medicine; 2015 Jun;22(3):365. PMID:25274015
145. Ratz T, Lippke S, Muellmann S, Peters M, Pischke CR, Meyer J, Bragina I, Voelcker-Rehage C. Effects of two web-based interventions and mediating mechanisms on stage of change regarding physical activity in older adults. Applied Psychology: Health & Well-Being; 2020 Mar;12(1):77–100. doi: 10.1111/aphw.12174
146. Ravicz M, Muhongayire B, Kamagaju S, Klabbers RE, Faustin Z, Kambugu A, Bassett I, O’Laughlin K. Using intervention mapping methodology to design an hiv linkage intervention in a refugee settlement in rural uganda. AIDS Care; 2022 Apr;34(4):446–458. doi: 10.1080/09540121.2021.1900532
147. Redsell SA, Rose J, Weng S, Ablewhite J, Swift JA, Siriwardena AN, Nathan D, Wharrad HJ, Atkinson P, Watson V, McMaster F, Lakshman R, Glazebrook C. Digital technology to facilitate Proactive Assessment of Obesity Risk during Infancy (ProAsk): A feasibility study; 2017 Sep;7(9):e017694. doi: 10.1136/bmjopen-2017-017694
148. Reeves D, Woodham AA, French D, Bower P, Holland F, Kontopantelis E, Cotterill S. The influence of demographic, health and psychosocial factors on patient uptake of the English NHS diabetes prevention programme. BMC Health Serv Res; 2023 Apr 11;23(1):352. doi: 10.1186/s12913-023-09195-z
149. Reichenberger J, Pannicke B, Arend A-K, Petrowski K, Blechert J. Does stress eat away at you or make you eat? EMA measures of stress predict day to day food craving and perceived food intake as a function of trait stress-eating. Psychology & Health; 2021 Feb;36(2):129–147. doi: 10.1080/08870446.2020.1781122
150. Roddy MK, Nelson LA, Greevy RA, Mayberry LS. Changes in family involvement occasioned by FAMS mobile health intervention mediate changes in glycemic control over 12 months. Journal of Behavioral Medicine; 2022 Feb;45(1):28–37. doi: 10.1007/s10865-021-00250-w
151. Rollo S, Prapavessis H. A combined health action process approach and mhealth intervention to increase non-sedentary behaviours in office-working adults—a randomised controlled trial. Applied Psychology: Health & Well-Being; 2020 Nov;12(3):660–686. doi: 10.1111/aphw.12201
152. Rollo S, Prapavessis H. A combined health action process approach and mHealth intervention to reduce workplace sitting time in office-working adults: A secondary analysis examining health-related quality of life and work performance outcomes. Psychology & Health; 2021 Oct;36(10):1200–1216. doi: 10.1080/08870446.2020.1838522
153. Rom S, Miskovic-Wheatley J, Barakat S, Aouad P, Fuller-Tyszkiewicz M, Maguire S. Evaluating the feasibility and potential efficacy of a brief eTherapy for binge-eating disorder: A pilot study. International Journal of Eating Disorders; 2022 Nov;55(11):1614–1620. doi: 10.1002/eat.23803
154. Rounsefell K, Gibson S, McLean S, Blair M, Molenaar A, Brennan L, Truby H, McCaffrey TA. Social media, body image and food choices in healthy young adults: A mixed methods systematic review. NUTR DIET; 2020 Feb;77(1):19–40. doi: 10.1111/1747-0080.12581
155. Ruf A, Koch ED, Ebner-Priemer U, Knopf M, Reif A, Matura S. studying microtemporal, within-person processes of diet, physical activity, and related factors using the appetite-mobile-app: Feasibility, usability, and validation study. J Med Internet Res; 2021 Jul 5;23(7):e25850. doi: 10.2196/25850
156. Ružić L, Mohar S, Radman I. Interactive dance: an exciting way to enhance children’s physical activity level. Kinesiology; 2014 Jan 2;46:66–73.
157. Sabir M, Henderson CR, Kang S-Y, Pillemer K. Attachment-focused integrative reminiscence with older African Americans: a randomized controlled intervention study. Aging & Mental Health; 2016 May;20(5):517–528. doi: 10.1080/13607863.2015.1023764
158. Saez L, Langlois J, Legrand K, Quinet M-H, Lecomte E, Omorou AY, Briancon S. Reach and acceptability of a mobile reminder strategy and facebook group intervention for weight management in less advantaged adolescents: Insights from the pralimap-ines trial. JMIR Mhealth Uhealth; 2018 May;6(5):e110. doi: 10.2196/mhealth.7657
159. Sakane N, Suganuma A, Domichi M, Sukino S, Abe K, Fujisaki A, Kanazawa A, Sugimoto M. The effect of a mhealth app (kenpo-app) for specific health guidance on weight changes in adults with obesity and hypertension: pilot randomized controlled trial. JMIR Mhealth Uhealth. 2023 Apr 12;11:e43236. PMID:37043287
160. Sanchez-Flack J, Buscemi J, O’Donnell A, Withington MHCH, Fitzgibbon M. Black american and latinx parent/caregiver participation in digital health obesity interventions for children: a systematic review. Front Digit Health; 2021 Jun 15;3:687648. doi: 10.3389/fdgth.2021.687648
161. Sánchez-Ortí JV, Balanzá-Martínez V, Correa-Ghisays P, Selva-Vera G, Vila-Francés J, Magdalena-Benedito R, San-Martin C, Victor VM, Escribano-Lopez I, Hernández-Mijares A, Vivas-Lalinde J, Crespo-Facorro B, Tabarés-Seisdedos R. Specific metabolic syndrome components predict cognition and social functioning in people with type 2 diabetes mellitus and severe mental disorders. Acta Psychiatrica Scandinavica; 2022 Sep;146(3):215–226. doi: 10.1111/acps.13433
162. Scharff A, Breiner CE, Ueno LF, Underwood SB, Merritt EC, Welch LM, Fonda C, Weil Malatras J, Lin B, Hormes JM, Pieterse AL, Gordis EB, Halpern LF, Pazienza R, Litchford GB. Shifting a training clinic to teletherapy during the COVID-19 pandemic: A trainee perspective. Counselling Psychology Quarterly; 2021 Dec;34(3/4):676–686. doi: 10.1080/09515070.2020.1786668
163. Schneiderman N, McIntosh RC, Antoni MH. Psychosocial risk and management of physical diseases. Journal of Behavioral Medicine; 2019 Feb;42(1):16–33. doi: 10.1007/s10865-018-00007-y
164. Schroeter C, Corder T, Brookes B, Reller V. An incentive-based health program using MyPlate: A pilot study analyzing college students’ dietary intake behavior. Journal of American College Health; 2021 Apr;69(3):252–259. doi: 10.1080/07448481.2019.1661845
165. Schwarzer R, Warner LM, Fleig L, Gholami M, Serra-Majem L, Ngo J, Cianferotti L, Kritikou M, Mossi P, Ntzani E, Brandi ML. Dietary planning, self-efficacy, and outcome expectancies play a role in an online intervention on fruit and vegetable consumption. Psychology & Health; 2018 May;33(5):652–668. doi: 10.1080/08870446.2017.1385785
166. Sebert Kuhlmann AK, Altman L, Galavotti C. The importance of community mobilization in interventions to improve sexual, reproductive, and maternal health outcomes: A review of the evidence. HEALTH CARE WOMEN INT; 2016 Oct;37(10):1028–1066. doi: 10.1080/07399332.2016.1141911
167. Sengupta A, Beckie T, Dutta K, Dey A, Chellappan S. A mobile health intervention system for women with coronary heart disease: Usability study. JMIR Form Res; 2020 Jun;4(6):e16420. doi: 10.2196/16420
168. Shaw PA, Yancy WS, Wesby L, Ulrich V, Troxel AB, Huffman D, Foster GD, Volpp K. The design and conduct of Keep It Off: An online randomized trial of financial incentives for weight-loss maintenance. Clin Trials; 2017 Feb;14(1):29–36. doi: 10.1177/1740774516669679
169. Shaw RJ, Bosworth HB, Silva SS, Lipkus IM, Davis LL, Sha RS, Johnson CM. Mobile health messages help sustain recent weight loss. Am J Med; 2013 Nov;126(11):1002–1009. doi: 10.1016/j.amjmed.2013.07.001
170. Shingleton RM, Pratt EM, Gorman B, Barlow DH, Palfai TP, Thompson-Brenner H. Motivational text message intervention for eating disorders: A single-case alternating treatment design using ecological momentary assessment. Behav Therapy; 2016 May;47(3):325–338. doi: 10.1016/j.beth.2016.01.005
171. Simmons LA, Phipps JE, Whipps M, Smith P, Carbajal KA, Overstreet C, McLaughlin J, De Lombaert K, Noonan D. From hybrid to fully remote clinical trial amidst the COVID-19 pandemic: Strategies to promote recruitment, retention, and engagement in a randomized mHealth trial. Digit Health; 2022 Sep;8:20552076221129065. doi: 10.1177/20552076221129065
172. Simone M, Emery RL, Hazzard VM, Eisenberg ME, Larson N, Neumark-Sztainer D. Disordered eating in a population-based sample of young adults during the COVID-19 outbreak. International Journal of Eating Disorders; 2021 Jul;54(7):1189–1201. doi: 10.1002/eat.23505
173. Smith JM, Whisenhunt BL, Buchanan EM, Hudson DL. Evaluating the effectiveness of ecological momentary assessment and intervention targeting body checking behaviors. Eating Disorders; 2019 Dec 11;27(6):521–537. doi: 10.1080/10640266.2018.1560850
174. Soto PL, Ramalingam L, George B, Moustaid-Moussa N. Long-term effects of adolescent exposure to olanzapine in C57BL/6 J mice and the impact of dietary fish oil supplementation. Psychopharmacology; 2022 Oct;239(10):3117–3131. doi: 10.1007/s00213-022-06193-7
175. Souliotis K, Giannouchos TV, Golna C, Liberopoulos E. Assessing forgetfulness and polypharmacy and their impact on health-related quality of life among patients with hypertension and dyslipidemia in Greece during the COVID-19 pandemic. Quality of Life Research; 2022 Jan;31(1):193–204. doi: 10.1007/s11136-021-02917-y
176. Spark LC, Fjeldsoe BS, Eakin EG, Reeves MM. Efficacy of a text message-delivered extended contact intervention on maintenance of weight loss, physical activity, and dietary behavior change. JMIR Mhealth Uhealth; 2015 Sep;3(3):e88. doi: 10.2196/mhealth.4114
177. Staffileno BA, Tangney CC, Fogg L, Darmoc R. Making behavior change interventions available to young African American women development and feasibility of an ehealth lifestyle program. J Cardiovasc Nurs; 2015 Dec;30(6):497–505. doi: 10.1097/JCN.0000000000000197
178. Steinberg DM, Levine EL, Lane I, Askew S, Foley PB, Puleo E, Bennett GG. Adherence to self-monitoring via interactive voice response technology in an ehealth intervention targeting weight gain prevention among black women: Randomized controlled trial. J Med Internet Res; 2014 Apr;16(4):105–116. doi: 10.2196/jmir.2996
179. Steinglass JE, Attia E, Glasofer DR, Wang Y, Ruggiero J, Walsh BT, Thomas JG. Optimizing relapse prevention and changing habits (REACH+) in anorexia nervosa. International Journal of Eating Disorders; 2022 Jun;55(6):851–857. doi: 10.1002/eat.23724
180. Steward WT, Sumitani J, Moran ME, Ratlhagana M-J, Morris JL, Isidoro L, Gilvydis JM, Tumbo J, Grignon J, Barnhart S, Lippman SA. Engaging HIV-positive clients in care: Acceptability and mechanisms of action of a peer navigation program in South Africa. AIDS Care; 2018 Mar;30(3):330–337. doi: 10.1080/09540121.2017.1363362
181. Stieger S, Lewetz D, Swami V. Emotional well-being under conditions of lockdown: an experience sampling study in austria during the covid-19 pandemic. Journal of Happiness Studies; 2021 Aug;22(6):2703–2720. doi: 10.1007/s10902-020-00337-2
182. Su Y, Wu K-C, Chien S-Y, Naik A, Zaslavsky O. A mobile intervention designed specifically for older adults with frailty to support healthy eating: pilot randomized controlled trial. JMIR Form Res; 2023 Nov 15;7:e50870. PMID:37966877
183. Taylor SS, Olsen MK, McVay MA, Grubber J, Gierisch JM, Yancy WS, Voils CI. The role of group cohesion in a group-based behavioral weight loss intervention. Journal of Behavioral Medicine; 2019 Feb;42(1):162–168. doi: 10.1007/s10865-018-9953-4
184. Tham XC, Whitton C, Mueller-Riemenschneider F, Petrunoff NA. Young adults’ use of mobile food delivery apps and the potential impacts on diet during the covid-19 pandemic: mixed methods study. JMIR Form Res; 2023;7. doi: 10.2196/38959
185. Thilsing T, Sonderlund AL, Sondergaard J, Svensson NH, Christensen JR, Thomsen JL, Hvidt NC, Larsen LB. Changes in health-risk behavior, body mass index, mental well-being, and risk status following participation in a stepwise web-based and face-to-face intervention for prevention of lifestyle-related diseases: nonrandomized follow-up cohort study. JMIR Public Health Surveill. 2020 Jul 9;6(3):e16083. PMID:32673269
186. Thompson RG, Elliott JC, Hu M-C, Aivadyan C, Aharonovich E, Hasin DS. Short-term effects of a brief intervention to reduce alcohol use and sexual risk among homeless young adults: Results from a randomized controlled trial. Addiction Research & Theory; 2017 Feb;25(1):24–31. doi: 10.1080/16066359.2016.1193165
187. Torbjørnsen A, Jenum AK, Småstuen MC, Årsand E, Holmen H, Wahl AK, Ribu L. A low-intensity mobile health intervention with and without health counseling for persons with type 2 diabetes, part 1: baseline and short-term results from a randomized controlled trial in the Norwegian part of renewing health. JMIR Mhealth Uhealth. 2014 Dec 11;2(4):e52. PMID:25499592
188. Torres DB, Lopes A, Rodrigues AJ, Lopes MG, Ventura-Silva AP, Sousa N, Gontijo JAR, Boer PA. Gestational protein restriction alters early amygdala neurochemistry in male offspring. Nutritional Neuroscience; 2023 Nov;26(11):1103–1119. doi: 10.1080/1028415X.2022.2131064
189. Tu AW, Watts AW, Chanoine J-P, Panagiotopoulos C, Geller J, Brant R, Barr SI, Mâsse L. Does parental and adolescent participation in an e-health lifestyle modification intervention improves weight outcomes? BMC Public Health; 2017;17. PMID:28438202
190. Tussing-Humphreys L, Thomson J, Onufrak S. A church-based pilot study designed to improve dietary quality for rural, lower mississippi delta, african american adults. Journal of Religion & Health; 2015 Apr;54(2):455–469. doi: 10.1007/s10943-014-9823-5
191. Valle CG, Queen TL, Martin BA, Ribisl KM, Mayer DK, Tate DF. Optimizing tailored communications for health risk assessment: A randomized factorial experiment of the effects of expectancy priming, autonomy support, and exemplification. J Med Internet Res; 2018 Mar;20(3):e63. doi: 10.2196/jmir.7613
192. Valshtein TJ, Oettingen G, Gollwitzer PM. Using mental contrasting with implementation intentions to reduce bedtime procrastination: Two randomised trials. Psychology & Health; 2020 Mar;35(3):275–301. doi: 10.1080/08870446.2019.1652753
193. Van Den Brekel-Dijkstra K, Rengers AH, Niessen MA, De Wit NJ, Kraaijenhagen RA. Personalized prevention approach with use of a web-based cardiovascular risk assessment with tailored lifestyle follow-up in primary care practice—a pilot study. Eur J Prev Cardiolog. 2016 Mar;23(5):544–551. doi: 10.1177/2047487315591441
194. van der Meer EWC, Boot CRL, van der Gulden JWJ, Knol DL, Jungbauer FHW, Coenraads PJ, Anema JR. Hands4U: the effects of a multifaceted implementation strategy on hand eczema prevalence in a healthcare setting. Results of a randomized controlled trial. Contact Dermatitis (01051873); 2015 May;72(5):312–324. doi: 10.1111/cod.12313
195. Van Der Windt M, Van Der Kleij RM, Snoek KM, Willemsen SP, Dykgraaf RHM, Laven JSE, Schoenmakers S, Steegers-Theunissen RPM. Impact of a blended periconception lifestyle care approach on lifestyle behaviors: before-and-after study. J Med Internet Res; 2020 Sep 30;22(9):e19378. doi: 10.2196/19378
196. van Dijk MR, Oostingh EC, Koster MPH, Willemsen SP, Laven JSE, Steegers-Theunissen RPM. The use of the mHealth program Smarter Pregnancy in preconception care: rationale, study design and data collection of a randomized controlled trial. BMC Pregnancy Childbirth; 2017 Jan 26;17:46. doi: 10.1186/s12884-017-1228-5
197. Vandermorris S, Au A, Gardner S, Troyer AK. Initiation and maintenance of behaviour change to support memory and brain health in older adults: A randomized controlled trial. Neuropsychological Rehabilitation; 2022 May;32(4):611–628. doi: 10.1080/09602011.2020.1841656
198. Walsh JC, Richmond J, Mc Sharry J, Groarke A, Glynn L, Kelly MG, Harney O, Groarke JM. Examining the impact of an mhealth behavior change intervention with a brief in-person component for cancer survivors with overweight or obesity: Randomized controlled trial. JMIR Mhealth Uhealth; 2021 Jul 5;9(7):e24915. doi: 10.2196/24915
199. Wang J, Cai C, Padhye N, Orlander P, Zare M. A behavioral lifestyle intervention enhanced with multiple-behavior self-monitoring using mobile and connected tools for underserved individuals with type 2 diabetes and comorbid overweight or obesity: pilot comparative effectiveness trial. JMIR Mhealth Uhealth; 2018 Apr;6(4):e92. doi: 10.2196/mhealth.4478
200. Wang XT, Wang P, Lu J, Zhou J, Li G, Garelik S. Episodic future thinking and anticipatory emotions: Effects on delay discounting and preventive behaviors during COVID-19. Applied Psychology: Health & Well-Being; 2022 Aug;14(3):842–861. doi: 10.1111/aphw.12350
201. Waterman EA, Edwards KM, Rodriguez LM, Ullman SE, Dardis CM. Predictors of uptake and retention in an intervention to improve social reactions to disclosures of sexual assault and partner abuse. Journal of American College Health; 2022 Jan;70(1):199–208. doi: 10.1080/07448481.2020.1739054
202. Weiner LS, Nagel S, Irene Su H, Hurst S, Levy SS, Arredondo EM, Hekler E, Hartman SJ. A remotely delivered, peer-led intervention to improve physical activity and quality of life in younger breast cancer survivors. Journal of Behavioral Medicine; 2023 Aug;46(4):578–593. doi: 10.1007/s10865-022-00381-8
203. Whatnall MC, Patterson AJ, Chiu S, Oldmeadow C, Hutchesson MJ. Feasibility and preliminary efficacy of the eating advice to students (eats) brief web-based nutrition intervention for young adult university students: a pilot randomized controlled trial. Nutrients Basel; 2019 Apr;11(4):905. doi: 10.3390/nu11040905
204. Whittaker AC, Chauntry AJ. Blunted cardiovascular reactivity to acute psychological stress predicts low behavioral but not self-reported perseverance: A replication study. Psychophysiology; 2021 Jan;58(1):1–12. doi: 10.1111/psyp.13707
205. Willems RA, Lechner L, Verboon P, Mesters I, Kanera IM, Bolman CAW. Working mechanisms of a web-based self-management intervention for cancer survivors: a randomised controlled trial. Psychology & Health; 2017 May;32(5):605–625. doi: 10.1080/08870446.2017.1293054
206. Winters B, Wopereis S, Bijlsma S, Krone T, den Broek TV, Caspers MPM, Anderson B, Nieman K, Anthony J. A novel personalized nutrition program improves health and lifestyle behaviors: subgroup analysis results from habit’s personalized approaches to health (path) study (p15-019-19). Curr Dev Nutr; 2019 Jun 13;3(Suppl 1):nzz037.P15-019-19. PMID:null
207. Wozniak J, DiSalvo M, Farrell A, Vaudreuil C, Uchida M, Ceranoglu TA, Joshi G, Cook E, Faraone SV, Biederman J. Findings from a pilot open-label trial of N-acetylcysteine for the treatment of pediatric mania and hypomania. BMC Psychiatry; 2022 May 3;22:314. PMID:35505312
208. Wright C, Barnett A, Campbell KL, Kelly JT, Hamilton K. Behaviour change theories and techniques used to inform nutrition interventions for adults undergoing bariatric surgery: A systematic review. NUTR DIET; 2022 Feb;79(1):110–128. doi: 10.1111/1747-0080.12728
209. Wu K-C, Su Y, Chu F, Chen AT, Zaslavsky O. Behavioral change factors and retention in web-based interventions for informal caregivers of people living with dementia: scoping review. J Med Internet Res; 2022 Jul 7;24(7):e38595. doi: 10.2196/38595
210. Wurst R, Kinkel S, Lin J, Goehner W, Fuchs R. Promoting physical activity through a psychological group intervention in cardiac rehabilitation: A randomized controlled trial. Journal of Behavioral Medicine; 2019 Dec;42(6):1104–1116. doi: 10.1007/s10865-019-00047-y
211. Xie X, Wang X, Li A, Yan Y, Lu T, Wu Y, et al. A study of the effectiveness of mobile health application in a self-management intervention for kidney transplant patients. Iran J Kidney Dis; 2023;17(5):263-270. PMID: 37838936
212. Yin Z, Lesser J, Paiva KA, Zapata J, Moreno-Vasquez A, Grigsby TJ, Ryan-Pettes SR, Parra-Medina D, Estrada V, Li S, Wang J. Using mobile health tools to engage rural underserved individuals in a diabetes education program in south texas: feasibility study. JMIR Mhealth Uhealth; 2020 Mar 24;8(3):e16683. doi: 10.2196/16683
213. Young CL, Mohebbi M, Staudacher HM, Kay-Lambkin F, Berk M, Jacka FN, O’Neil A. Optimizing engagement in an online dietary intervention for depression (my food & mood version 3.0): Cohort study. JMIR Ment Health; 2021 Mar 31;8(3):e24871. doi: 10.2196/24871
214. Yuhas M, Porter KJ, Brock D-JP, Loyd A, McCormick BA, Zoellner JM. Development and pilot testing of text messages to help reduce sugar-sweetened beverage intake among rural caregivers and adolescents: Mixed methods study. JMIR Mhealth Uhealth; 2019 Jul 30;7(7):e14785. PMID:31364600
215. Zahrt OH, Evans K, Murnane E, Santoro E, Baiocchi M, Landay J, Delp S, Crum A. Effects of wearable fitness trackers and activity adequacy mindsets on affect, behavior, and health: longitudinal randomized controlled trial. J Med Internet Res; 2023 Jan 25;25:e40529. doi: 10.2196/40529
216. Zamanillo-Campos R, Fiol-deRoque MA, Serrano-Ripoll MJ, Mira-Martinez S, Ricci-Cabello I. Development and evaluation of DiabeText, a personalized mHealth intervention to support medication adherence and lifestyle change behaviour in patients with type 2 diabetes in Spain: A mixed-methods phase II pragmatic randomized controlled clinical trial. Int J Med Inform; 2023 Aug;176:105103. doi: 10.1016/j.ijmedinf.2023.105103

**Excluded Group 3: Minor or Unclear Relevance to Study**

1. Blair CK, Madan-Swain A, Locher JL, Desmond RA, de Los Santos J, Affuso O, Glover T, Smith K, Carley J, Lipsitz M, Sharma A, Krontiras H, Cantor A, Demark-Wahnefried W. Harvest for health gardening intervention feasibility study in cancer survivors. Acta Oncologica; 2013 Aug;52(6):1110–1118. doi: 10.3109/0284186X.2013.770165
2. Bouhaidar CM, Deshazo JP, Puri P, Gray P, Robins JLW, Salyer J. Text messaging as adjunct to community-based weight management program. CIN-Comput Inform Nurs; 2013 Oct;31(10):469–476. doi: 10.1097/01.NCN.0000432121.02323.cb
3. Chesnut RP, Czymoniewicz-Klippel M, DiNallo JM, Perkins DF. Grow online: Feasibility and proof of concept study. J Child Serv; 2019 Dec 3;15(1):25–42. doi: 10.1108/JCS-10-2018-0026
4. Chew CSE, Davis C, Lim JKE, Lim CMM, Tan YZH, Oh JY, Rajasegaran K, Chia YHM, Finkelstein EA. Use of a mobile lifestyle intervention app as an early intervention for adolescents with obesity: Single-cohort study. J Med Internet Res; 2021 Sep 28;23(9):e20520. doi: 10.2196/20520
5. Dabbas M, Lepage G, Boedoz E, Charrat A, Consfroy M, Manh Y. Use of new technologies for the follow-up in adolescent obesity; Mobile health intervention (MHI) a randomized controlled trial. Abstracts. BMJ Publishing Group Ltd and Royal College of Paediatrics and Child Health; 2019. p. A366.2-A366. doi: 10.1136/archdischild-2019-epa.867
6. Decorte E, Wilson Barnes S, Hart K, Stefanidis K, Gymnopoulos L, Rouskas K, Argiriou A, Leoni R, Russell D, Lalama E, Pfeiffer AFH, Pagkalos I, Dias SB, Batista A, Cornelissen V. A pilot trial guiding development and investigating feasibility and usability as well as preliminary effectiveness of the PROTEIN application: Personalized nutrition for healthy living. European Journal of Preventive Cardiology; 2023 May 24;30(Supplement_1):zwad125.220. doi: 10.1093/eurjpc/zwad125.220
7. Downs DS, Savage JS, Rivera DE, Pauley AM, Leonard KS, Hohman EE, Guo P, McNitt KM, Stetter C, Kunselman A. Adaptive, behavioral intervention impact on weight gain, physical activity, energy intake, and motivational determinants: results of a feasibility trial in pregnant women with overweight/obesity. J Behav Med; 2021 Oct;44(5):605–621. doi: 10.1007/s10865-021-00227-9
8. Du H, Venkatakrishnan A, Youngblood GM, Ram A, Pirolli P. A group-based mobile application to increase adherence in exercise and nutrition programs: A factorial design feasibility study. JMIR Mhealth Uhealth; 2016 Mar;4(1):140–155. doi: 10.2196/mhealth.4900
9. Elbert SP, Dijkstra A, Oenema A. A mobile phone app intervention targeting fruit and vegetable consumption: The efficacy of textual and auditory tailored health information tested in a randomized controlled trial. J Med Internet Res; 2016 Jun 10;18(6):e147. PMID:27287823
10. Erdil D, Koku Aksu AE, Falay Gür T, Gürel MS. Hand eczema treatment: Change behaviour with text messaging, a randomized trial. Contact Dermatitis (01051873); 2020 Mar;82(3):153–160. doi: 10.1111/cod.13448
11. Goni L, de la O V, Barrio-López MT, Ramos P, Tercedor L, Ibañez-Criado JL, Castellanos E, Ibañez Criado A, Macias Ruiz R, García-Bolao I, Almendral J, Martínez-González MÁ, Ruiz-Canela M. A Remote Nutritional intervention to change the dietary habits of patients undergoing ablation of atrial fibrillation: Randomized controlled trial. J Med Internet Res; 2020 Dec 7;22(12):e21436. PMID:33284131
12. Hu L, Islam N, Trinh-Shevrin C, Wu B, Feldman N, Tamura K, Jiang N, Lim S, Wang C, Bubu OM, Schoenthaler A, Ogedegbe G, Sevick MA. A social media-based diabetes intervention for low-income mandarin-speaking chinese immigrants in the united states: feasibility study. JMIR Form Res; 2022 May;6(5):e37737. doi: 10.2196/37737
13. Jefferson K, Ward M, Pang W-H, Arcand J. A feasibility study of a randomized controlled trial protocol to assess the impact of an eHealth intervention on the provision of dietary advice in primary care. Pilot Feasibility Stud; 2022 Sep 14;8(1):208. doi: 10.1186/s40814-022-01168-z
14. Kutzinski M, Krause N, Riemann-Lorenz K, Meyer B, Heesen C. Acceptability of a digital health application to empower persons with multiple sclerosis with moderate to severe disability: Single-arm prospective pilot study. BMC Neurol; 2023 Oct 23;23(1):382. doi: 10.1186/s12883-023-03434-w
15. Lin M, Mahmooth Z, Dedhia N, Frutchey R, Mercado CE, Epstein DH, Preston KL, Gibbons MC, Bowie JV, Labrique AB, Cheskin LJ. Tailored, interactive text messages for enhancing weight loss among african american adults: the TRIMM randomized controlled trial. Am J Med; 2015 Aug;128(8):896–904. doi: 10.1016/j.amjmed.2015.03.013
16. Lin P, Wang Y, Levine E, Askew S, Lin S, Chang C, Sun J, Foley P, Wang H, Li X, Bennett GG. A text messaging-assisted randomized lifestyle weight loss clinical trial among overweight adults in Beijing. Obesity; 2014 May;22(5). doi: 10.1002/oby.20686
17. Mason AE, Jhaveri K, Cohn M, Brewer JA. Testing a mobile mindful eating intervention targeting craving-related eating: Feasibility and proof of concept. Journal of Behavioral Medicine; 2018 Apr;41(2):160–173. doi: 10.1007/s10865-017-9884-5
18. Norton MC, Clark CJ, Tschanz JT, Hartin P, Fauth EB, Gast JA, Dorsch TE, Wengreen H, Nugent C, Robinson WD, Lefevre M, McClean S, Cleland I, Schaefer SY, Aguilar S. The design and progress of a multidomain lifestyle intervention to improve brain health in middle-aged persons to reduce later alzheimer’s disease risk: The gray matters randomized trial. Alzheimers Dement (N Y); 2015 Jun;1(1):53–62. doi: 10.1016/j.trci.2015.05.001
19. Opie RS, O’Neil A, Jacka FN, Pizzinga J, Itsiopoulos C. A modified Mediterranean dietary intervention for adults with major depression: Dietary protocol and feasibility data from the SMILES trial. Nutritional Neuroscience; 2018 Sep;21(7):487–501. doi: 10.1080/1028415X.2017.1312841
20. Pemu P, Josiah Willock R, Alema-Mensah E, Rollins L, Brown M, Saint Clair B, Olorundare E, McCaslin A, Henry Akintobi T, Quarshie A, Ofili E. Achieving health equity with e-healthystrides©: patient perspectives of a consumer health information technology application. Ethn Dis; 2019 Jun 13;29(Supp2):393–404. doi: 10.18865/ed.29.S2.393
21. Pope ZC, Gao Z. Feasibility of smartphone application- and social media-based intervention on college students’ health outcomes: A pilot randomized trial. Journal of American College Health; 2022 Jan;70(1):89–98. doi: 10.1080/07448481.2020.1726925
22. Vergeld V, Wienert J, Reinwand DA, Tan SL, Lippke S. An 8-week study on social-cognitive variables for physical activity and fruit and vegetable intake: Are there stage transitions? Applied Psychology: Health & Well-Being; 2021 Feb;13(1):109–128. doi: 10.1111/aphw.12218
23. Vidmar AP, Salvy SJ, Pretlow R, Mittelman SD, Wee CP, Fink C, Fox DS, Raymond JK. An addiction-based mobile health weight loss intervention: Protocol of a randomized controlled trial. Contemp Clin Trials; 2019 Mar;78:11–19. doi: 10.1016/j.cct.2019.01.008
24. Voils CI, Adler R, Strawbridge E, Grubber J, Allen KD, Olsen MK, McVay MA, Raghavan S, Raffa SD, Funk LM. Early-phase study of a telephone-based intervention to reduce weight regain among bariatric surgery patients. Health Psychol; 2020 May;39(5):391–402. PMID:31999175
25. Wang E, Keller H, Mourtzakis M, Rodrigues IB, Steinke A, Ashe MC, Thabane L, Brien S, Funnell L, Cheung AM, Milligan J, Papaioannou A, Weston ZJ, Straus S, Giangregorio L. MoveStrong at home: A feasibility study of a model for remote delivery of functional strength and balance training combined with nutrition education for older pre-frail and frail adults. Applied Physiology, Nutrition & Metabolism; 2022 Dec;47(12):1172–1186. doi: 10.1139/apnm-2022-0195
26. Wright C, Kelly JT, Byrnes J, Campbell KL, Healy R, Musial J, Hamilton K. A non-randomised feasibility study of a mHealth follow-up program in bariatric surgery. Pilot Feasibility Stud; 2023 Oct 17;9(1):176. doi: 10.1186/s40814-023-01401-3
