# Supplementary material for: Framework Development for Reducing Attrition in Digital Dietary Interventions: Systematic Review and Thematic Synthesis
Source: J Med Internet Res. 2024 Aug 27;26:e58735. doi: 10.2196/58735 (PMC11387916; doi:10.2196/58735)
Supplement: Multimedia Appendix 6 [file jmir_v26i1e58735_app6.doc]

**Multimedia Appendix 6: Study Characteristics**

***Table 1.*** *Study characteristics (part 1).*

| Author, year | Study date | Targeted dietary behavior | Duration |
| --- | --- | --- | --- |
| Brewer et al [11], 2016 | Period: May 2015 – August 2015 Publication: 2016 | Increasing the intake of fruits and vegetables among the participants | 4 months |
| Browne et al [42], 2020 | Period: 2018–2019 Publication: February 2021 | Reducing the rate of eating among children with obesity | 4 weeks |
| Cheung et al [43], 2019 | Publication: March 11, 2019. | The intervention targeted dietary behaviors by including text messages that promoted Australian dietary guidelines after pregnancy. This included advice on controlling carbohydrate intake and the use of low carbohydrate vegetables and foods to satiate hunger. | 6 months |
| Coa & Patrick [10], 2016 | Period: June 2014 – December 2014 Publication: 2016 | Behaviors related to healthy eating | 6 weeks |
| Dawson et al [12], 2021 | Period: 2017–2018 Publication: 2021 | Improving renal dietary behaviors related to potassium, phosphorus, sodium, and fluid intake, and general healthy eating and lifestyle behaviors. | 6 months |
| Grutzmacher et al [15], 2019 | Period: September 2012 – June 2013 Publication: 2019 | Nutrition and physical activity | 282 days |
| Hawkes et al [44], 2023 | Period: December 2020 – June 2021 Publication: 2023 | Improving diet as one of its main objectives, alongside increasing physical activity and achieving weight loss, to reduce the risk of type 2 diabetes. | 9 months |
| Howarth et al [18], 2019 | Period: 2015–2017 Acceptance: May 4, 2019 | Focusing on resilience, movement, eating, and sleep. | / |
| Jiang et al [45], 2023 | Period: December 1, 2010 – October 30, 2012 Publication: March 17, 2014 | Targeting optimal nutritional intake using ordinary food and oral nutrition supplements (ONS), tailored to individual needs, preferences, and diet restrictions. | 12 weeks |
| Kaul et al [13], 2022 | Period: September 2018 – December 2019 Publication: July 25, 2022 | Tracking dietary intake and identifying dietary factors that might influence pain symptoms | 12 weeks |
| Linardon et al [14], 2022 | Publication: 2020 | Eating disorders, particularly behaviors related to binge eating. | 8 weeks |
| Paxton et al [17], 2017 | Period: June 2014 – October 2015 Publication: 2017 | Increasing the intake of fiber, fruits, vegetables, and reducing saturated and trans fats | 3 months |
| Plaete et al [46], 2016 | Period: November 2014 – June 2015 Publication: 2016 | Increasing the intake of fruit and vegetable to promote healthier dietary habits among adults | 1 month |
| Rom et al [16], 2023 | Period: 2021–2022 Publication: October 4, 2023 | The intervention targeted behaviors associated with binge-eating disorder, focusing on establishing regular eating patterns, self-monitoring of food intake, and addressing thoughts and feelings related to eating and body image. | 10 weeks |
| Schulz et al [8], 2013 | Period: June 2010 – January 2011 Publication: 2013 | Reducing alcohol consumption among adult problem drinkers | 6 months |
| Silina et al [47], 2017 | Acceptance: June 6, 2017 | Increasing physical activity and dietary recommendations for dyslipidemia and weight loss | 1 year |
| Springer et al [48], 2018 | Publication: 2018 | Increasing fruit and vegetable intake | 28 days |
| Van der Mispel et al [49], 2017 | Period: November 2014 – September 2016 Publication: 2017 | Increasing fruit and vegetable intake | 1 month |
| Whitley et al [50], 2020 | Publication: June 2021 | Healthy eating and active living behaviors | 4 months |
| Young et al [51], 2021 | Period: 2018–2019 Publication: 2021 | Mediterranean diet | 8 weeks |
| Yuhas et al [52], 2023 | Acceptance: February 10, 2023 | Reducing the intake of sugar-sweetened beverages (SSBs) by adolescents | 6 months |

***Table 2.*** *Study characteristics (part 2) for randomized controlled trials (RCTs).*

| Author, year | Theories or behavioral techniques | Control group strategy | Intervention group strategy |
| --- | --- | --- | --- |
| Brewer et al [11], 2016 | / | Received educational tools including a phytochemical guide, recipe cards, and phytochemical health information cards. | Received a series of 5 nutrition education lessons focused on fruit and vegetable phytochemicals, as well as the same educational tools as the control group. |
| Browne et al [42], 2020 | Techniques: goals and planning, feedback and monitoring, social support, shaping knowledge, comparison of behaviour, repetition and substitution, and antecedents. | Received usual clinical care for pediatric obesity treatment over a 4-week period | Received usual clinical care plus additional training to reduce their rate of eating using the Mandolean app over a 4-week period |
| Cheung et al [43], 2019 | / | Received a standard paper-based booklet with information on healthy lifestyle after gestational diabetes. | Received a digital health support program involving customized mobile phone text messages and use of a Fitbit activity monitor linked to the text messaging system. |
| Dawson et al [12], 2021 | / | Received standard care provided by the dialysis unit. | Received standard care plus three text messages per week over a 6-month period containing advice, information, motivation and support related to dietary behaviors. |
| Jiang et al [45], 2023 | Theory: health action process approach (HAPA) theory | Received usual care which included a participant handbook about nutrition, a food atlas, and timepoints for outcome assessments. | Received usual care plus a 12-week individualized mobile health (mHealth) nutrition intervention delivered via a WeChat applet and biweekly phone nutrition consultations. |
| Kaul et al [13], 2022 | Theory: social cognitive theory Techniques: motivational interviewing, goal setting, and behavior reinforcement. | Received access to the mobile health app without any coaching sessions. | Received access to the mobile health app along with weekly health coaching sessions and personalized recommendations based on logged symptom, diet, and behavior data. |
| Linardon et al [14], 2022 | Techniques: transdiagnostic cognitive-behavioral therapy for eating disorders, integrating elements of dissonance-and acceptance-based approaches. | / | Received access to a transdiagnostic cognitive-behavioral therapy smartphone app called Break Binge Eating. |
| Plaete et al [46], 2016 | Theory: self-regulation theory Techniques: tailored feedback, goal setting, implementation intentions, problem-solving, and self-monitoring. | Received general information about health behavior recommendations via a website. | Received a web-based self-regulation intervention called MyPlan 1.0, which provided personalized feedback and promoted goal setting and planning for health behaviors. |
| Schulz et al [8], 2013 | Theories: I-change model, combining various psychological models and focusing on knowledge, awareness, attitude, social influence, self-efficacy, and action planning. | Received identical questionnaires at baseline, 3 months, and 6 months, but no intervention | Received a 3-session web-based tailored alcohol intervention at baseline, 3 months, and 6 months. The tailored feedback focused on knowledge, awareness, attitude, social influence, self-efficacy, and action planning. |
| Silina et al [47], 2017 | Theories: planned behavioral theory, and social cognitive theory. | Received advice on behavioral lifestyle changes including diet and physical activity recommendations for weight loss | Received advice on behavioral lifestyle changes as the control group plus SMS text messages with additional tips and reminders about weight loss once every two weeks over one year |
| Springer et al [48], 2018 | Theory: self-affirmation theory | No self-affirmation exercises | Received an initial self-affirmation exercise and ongoing booster self-affirmations delivered through a mobile app |
| Yuhas et al [52], 2023 | Theory: theory of planned behavior | / | Received a 6-month SMS program to reduce SSB intake |

**Table 3.** Study characteristics (part 3) for observational trials.

| Author, year | Theories or behavioral techniques | Intervention strategy |
| --- | --- | --- |
| Coa & Patrick [10], 2016 | Theory: self-determination theory (SDT) | This was achieved through the HYTxt program, which delivered text messages including standardized behavioral intervention and social support messages focusing on specific health behavior goals related to diet. |
| Grutzmacher et al [15], 2019 | / | The intervention was a text-based nutrition and physical activity promotion program delivered via SMS text messages. |
| Hawkes et al [44], 2023 | Techniques: monitoring behaviors, setting goals, and providing educational content and social support. | The NHS-DDPP was a digital behavior change intervention. Providers delivered their versions of the digital program, including features like goal setting, self-monitoring, educational content, and social support, through apps and other digital platforms. |
| Howarth et al [18], 2019 | Theories: person-based approach, incorporating theories like the trans-theoretical model, social cognitive theory, and self-efficacy theory.  Techniques: focusing on small steps, readiness to take action, and supporting self-efficacy. | The intervention strategy involved developing a digital health intervention using the Person-Based Approach, which included qualitative data collection, feedback integration, and iterative design adjustments based on user input. |
| Paxton et al [17], 2017 | Theories: social cognitive theory, goal-setting theory, social marketing, and the transtheoretical model.  Techniques: breaking up large goals into small achievable weekly goals, self-monitoring, rewards, cues to action, and repetition. | The intervention used a fully automated system providing tools for self-monitoring and goal setting, tailored content, and automated phone calls, all delivered through email. |
| Rom et al [16], 2023 | Techniques: cognitive-behavioral therapy, including techniques like regular eating, self-monitoring, exposure challenges, thought challenging, and emotional regulation frameworks. | The intervention was a supported Self-Help Binge-Eating eTherapy (SSH-BEeT) program, delivered online, focusing on cognitive-behavioral therapy-based principles. |
| Van der Mispel et al [49], 2017 | Theory: self-regulation theory Techniques: planning, coping planning, and self-monitoring. | A web-based intervention. Techniques are designed to help individuals plan and monitor their behaviors in relation to physical activity and healthy eating, particularly increasing fruit and vegetable intake. The intervention aimed to facilitate behavior change by helping participants set specific goals, plan for potential obstacles, and track their progress over time. |
| Whitley et al [50], 2020 | Theory: health belief model | The strategy involved sending mobile messages (text and email) in English or Spanish to congregants. These messages included health promotion content related to dietary and physical activity behaviors. |
| Young et al [51], 2021 | Theories: self-determination theory (SDT) and the transtheoretical model.  Techniques: information about health consequences, action planning, credible source, goal setting (behavior), feedback on behavior, self-monitoring of behavior. | The strategy involved a smartphone application delivering a Mediterranean diet intervention, including self-monitoring tools for food, mood, and lifestyle, as well as tools for goal setting and shopping. |
