# Supplementary material for: Framework Development for Reducing Attrition in Digital Dietary Interventions: Systematic Review and Thematic Synthesis
Source: J Med Internet Res. 2024 Aug 27;26:e58735. doi: 10.2196/58735 (PMC11387916; doi:10.2196/58735)
Supplement: Multimedia Appendix 7 [file jmir_v26i1e58735_app7.doc]

**Multimedia Appendix 7 Study Quality Appraisal**

Tables 1-6 summarize the information collected using the question checklist and evaluation form from Multimedia Appendix 4. Tables 7-8 provide a summary of the scores and an evaluation of the ratings.

***Table 1.*** *Sample characteristics: transferability considerations (part 1).*

| Author, year | Eligibility criteria | Country | Sample size | Age | Gender |
| --- | --- | --- | --- | --- | --- |
| Brewer et al [11], 2016 | Participants were community-dwelling, noninstitutionalized older adults aged 60 years or older attending local senior centers. Exclusions were for homebound or cognitively impaired individuals. | Kentucky, United States | 64 | 62–93 | 87.5% were female in the control group, 79% were female in the intervention group. |
| Browne et al [42], 2020 | Participants were children and adolescents aged 9–16 years with obesity (BMI ≥98th percentile), recruited from a weight management service. | Republic of Ireland | 20 | 9–16 | 9 males (45%) and 11 females (55%) |
| Cheung et al [43], 2019 | Eligibility criteria included gestational diabetes diagnosed by the 1998 Australasian Diabetes in Pregnancy Society Criteria, a fasting glucose level ≥5.5 mmol/L and/or a 2-h glucose level ≥8 mmol/L on a 75 g oral glucose tolerance test, age ≥18 years, owning a smart mobile phone with text messaging capability, having internet access, and being physically capable of performing moderate-intensity physical activity. | Australia | 60 | Mean age: approximately 34, with a standard deviation of 4. | Female |
| Coa & Patrick [10], 2016 | / | United States | 193 | 13–62 Mean age: 34.6 | Gender information was not collected, but the program was promoted on a platform presumed to be primarily used by women. |
| Dawson et al [12], 2021 | Participants had to have end-stage kidney disease, be on maintenance hemodialysis for at least 90 days, be aged 18 years or over, have sufficient English skills to read and understand text messages, and access to a mobile phone. | Australia | 130 | / | / |
| Grutzmacher et al [15], 2019 | Participants were parents of children attending low-income elementary schools with youth SNAP-Ed programs in Maryland and participating in the Text2BHealthy program. | United States, specifically from Maryland | 972 | / | / |
| Hawkes et al [44], 2023 | Participants were adults living in England, identified as high-risk for developing type 2 diabetes. They were either referred through their general practice with non-diabetic hyperglycemia or through an online self-referral questionnaire. Exclusions included being pregnant or under 18 years old. | England | 1826 | / | / |
| Howarth et al [18], 2019 | / | With Cigna colleagues from various international offices contributing to the development process. | / | 25–55 | 65% Female |
| Jiang et al [45], 2023 | The eligibility criteria included patients with histologically confirmed gastric adenocarcinoma who had received D2 radical gastrectomy, had access to broadband internet, were aged 18 years or older, and agreed to participate in the trial through informed consent. The exclusion criteria included severe post- operative morbidity, evidence of active or recurrent disease (cardiovascular, respiratory, kidney, liver, cerebrovascular diseases, etc.), existence of other malignancies within the last 5 years, and diagnosed vision, hearing, or speech impairment. | China | 24 | / | 66.7% male (16 participants) |
| Kaul et al [13], 2022 | Participants had to be 55 years or older, experiencing chronic non-cancer pain most days in the past three months, have an average pain level of 4 or greater on a 0-to-10 scale, have pain-related interference in daily activities, and access to a smartphone or tablet. | United States (New York City) | 31 | / | 54% female |
| Linardon et al [14], 2022 | Participants were eligible if they were 18 years or older, owned a smartphone, and self-reported at least one objective binge eating episode over the past 4 weeks. | Australian or international | 392 | Mean age: 28.95 | 93% female |
| Paxton et al [17], 2017 | Eligibility criteria included a previous diagnosis of breast cancer, being at least 18 years old at study enrollment, having completed treatment (except hormonal therapy) at least 6 months before study enrollment, and being receptive to participating in a Web-based intervention study. Participants who self-identified as African American, Hispanic, or of mixed ethnicity were eligible. | United States | 71 (37 for diet) | 26–72 Mean age: 52 | Female |
| Plaete et al [46], 2016 | Participants were adults (≥18 years) who had access to the Internet and could speak Dutch. They were recruited from general practices in Flanders. | Belgium, specifically the Flemish region | 426 | / | / |
| Rom et al [16], 2023 | Participants were required to meet the DSM-5 criteria for BED or Other Specified Feeding or Eating Disorder with BED behaviors (subthreshold BED), have a minimum of one or more weekly objective binge episodes in the preceding 2 months, be 16 years or older, have a BMI equal to or greater than 20, and access to internet and a digital device with a video camera. | Australia | 19 | 19.55–53.81  Mean age: 37.69 | Female |
| Schulz et al [8], 2013 | Participants needed to be members of an online panel, have computer/Internet literacy, sufficient command of German, be 18 years or older, and exhibit an unhealthy drinking pattern, which was defined by several specific criteria. | Germany | 448 | Mean age: approximately 42 | 56.5% male (253 participants) and 43.5% female (195 participants) |
| Silina et al [47], 2017 | Eligibility criteria included overweight and obese men and women aged 30–45 years without cardiovascular diseases (CVDs) or diabetes. | Latvia | 129 | 30–45 | / |
| Springer et al [48], 2018 | Participants were required to be over 18 years old, possess a laptop or desktop computer and a mobile phone running Android or iOS, and have fruit and vegetable consumption below recommended levels (5 combined servings per day). | San Francisco Bay Area, United States | 127 | / | 90 female, 36 male, and 1 nonbinary. |
| Van der Mispel et al [49], 2017 | Minimum age of 18 years, recruited via general practice settings. | Belgium | 422 | 18–81 Mean age: 43.92 | 55.7% female, 28.2% male |
| Whitley et al [50], 2020 | The study involved congregants from predominantly African American or Latino churches who were part of a church-based program. | United States, specifically from Los Angeles County, California | 131 | Most were over 50 | Primarily involved female participants |
| Young et al [51], 2021 | Eligible individuals were those aged over 18 years and reported current depressive symptoms (score of >5 on PHQ-8). They were excluded if they did not have access to the internet, a computer or smartphone, had low computer literacy, limited English literacy, already followed a high-quality diet, had no diet autonomy, or risk of eating disorder. | The majority of the participants were from Australia, with some participants from other countries including Canada, Denmark, Germany, Hungary, Israel, Malaysia, New Zealand, Switzerland, the UK, and the USA. | 128 | Median age: 42.5, with the first and third quartiles being 33 and 50 respectively. | 83% female (125 participants) |
| Yuhas et al [52], 2023 | Caregivers were required to have a functioning mobile phone/phone number and provide written or verbal consent for themselves and their child. | United States | 357 | 35–44 (52% participants) | 91% Female |

***Table 2.*** *Sample characteristics: transferability considerations (part 2).*

| Author, year | Ethnicity | Socio-economic status | Other characteristics |
| --- | --- | --- | --- |
| Brewer et al [11], 2016 | 81.3% were White in the control group, 84.2% were White in the intervention group. | 87.5% had completed high school or more | No tobacco use was reported by a majority of participants, and most self-reported their health as at least good. |
| Browne et al [42], 2020 | / | / | / |
| Cheung et al [43], 2019 | South Asian population | / | The study specifically targeted women with gestational diabetes, owning a smart mobile phone with text messaging capability, having internet access, and being physically capable of performing moderate-intensity physical activity. |
| Coa & Patrick [10], 2016 | 48.7% were white, 18.1% non-white, with 33.2% missing data. | Most users had at least some college education. | / |
| Dawson et al [12], 2021 | / | / | / |
| Grutzmacher et al [15], 2019 | / | Participants were parents of children attending low-income elementary schools. | / |
| Hawkes et al [44], 2023 | / | / | / |
| Howarth et al [18], 2019 | / | / | The intervention population consisted of Cigna colleagues from a variety of departments, including sales, business intelligence, and account management. These colleagues had real-world experience in the procurement and implementation of workplace interventions. |
| Jiang et al [45], 2023 | / | / | 33.3% employed, 16.7% unemployed, and 41.7% retired. Additionally, it mentions the tumor location and pathological stage among participants. |
| Kaul et al [13], 2022 | 54% White, 38% Black, 8% other. | Includes participants with varying educational backgrounds: some college or less, college graduates, and those with postgraduate degrees. | Older adults with chronic non-cancer pain (CNCP) living in an urban setting. |
| Linardon et al [14], 2022 | 76% White/Caucasian | 82% of the participants had tertiary education. | / |
| Paxton et al [17], 2017 | The majority of participants were African American (83%), followed by Hispanic (11%), and mixed ethnicity (6%). | The majority of participants were college graduates (65%). | Most participants were diagnosed with regional stage disease (54%), and many were obese (52%). Approximately 63% were already meeting current guidelines for physical activity at baseline. |
| Plaete et al [46], 2016 | / | / | / |
| Rom et al [16], 2023 | The majority were Australian (63.16%), with the rest identifying as Aboriginal or Torres Strait Islander (5.26%), New Zealand (10.53%), South American (5.26%), or multiple races (15.79%). | The majority (78.95%) had a bachelor’s degree or post-graduate study. Employment status varied, with 52.63% full-time employed, 36.84% part-time, and 10.53% unemployed or students. | Most participants (74%) reported secondary mental health concerns, predominantly anxiety (47%). The majority (68.42%) resided in metropolitan areas. |
| Schulz et al [8], 2013 | / | The socio-economic status of the participants included a range of education levels and incomes. Specifically, the educational distribution was 42% low, 24% medium, and 34% high education. Income brackets were also varied, with 13.6% earning below €1000, 23.7% earning between €1001–€2000, 30.1% earning between €2001–€4000, and 9.6% earning over €4000 monthly. | Other demographic details like marital status, employment situation, and presence of children were recorded. The study also accounted for health status factors such as diabetes, angina pectoris, cancer, high blood pressure, and depression symptoms. |
| Silina et al [47], 2017 | / | / | The participants were clinically healthy individuals with a body mass index (BMI) above 25 kg/m^2. |
| Springer et al [48], 2018 | / | / | / |
| Van der Mispel et al [49], 2017 | / | 41.7% with a low educational level, 42.2% with a high educational level. | 48.3% overweight, 48.6% normal weight, mean BMI 25.96 kg/m² |
| Whitley et al [50], 2020 | Predominantly African American or Latino. | Varied across church types. For African American church participants, 90% had at least some post-high school education, compared to 38% among Latino participants. | Fewer than 5% of African American church participants were born outside the United States, compared to over 70% at the Latino church. At the Latino church, 33% of participants reported having no health insurance compared to only 6% at the African American churches. |
| Young et al [51], 2021 | / | / | A third of the participants were taking antidepressants, and most rated their computer confidence as high with a skill level of competent or highly skilled. |
| Yuhas et al [52], 2023 | 95% White | 68% had at least some college education, 56% reported an annual household income of $50,000 or more. | / |

***Table 3.*** *Trustworthiness: evidence of rigour (part 1).*

| Author, year | Recruitment | Data collection | Analysis method and rationale |
| --- | --- | --- | --- |
| Brewer et al [11], 2016 | Participants were recruited through advertisements in the senior center newsletter, flyers, table tents at each center, and encouragement from senior center directors. | The attrition rate was determined based on the number of participants who completed the study compared to the number who initially enrolled. | / |
| Browne et al [42], 2020 | Participants were recruited from a weight management service at a tertiary health care center. They received information leaflets, and informed consent and assent were obtained. The inclusion criteria were children aged 9–16 years with obesity (BMI ≥98th percentile). | The study assessed attrition rates by comparing the number of participants who started the study with those who completed it. Data collection methods included questionnaires, anthropometry, and app usage data. | The study used descriptive frequencies, standard t-tests, and content analysis of qualitative feedback to analyze the data. The rationale was to evaluate the feasibility and acceptability of the intervention. |
| Cheung et al [43], 2019 | Women were recruited while attending a dedicated Diabetes in Pregnancy antenatal clinic. They were largely those who required insulin, and recruitment took place when they were 24–30 weeks pregnant. | Data collection on attrition rates involved recording the number of participants who completed all elements of the final evaluation. This included keeping a log of contacts that subjects made with the study team and reasons for contact. | The analysis for attrition rate was conducted using intention-to-treat principles. The researchers analyzed the completion of study elements and used statistical tools to compare characteristics of participants who completed the study versus those who did not. |
| Coa & Patrick [10], 2016 | Users enrolled in the HYTxt program through texting a keyword on their phone or via a web enrollment form. | The study collected data on attrition rate through participant self-reporting during program enrollment and throughout the intervention. Participants' engagement and dropout were monitored based on their interaction with the text messaging program. | The analysis method involved logistic regression models. These models examined the association between baseline motivation type (autonomous vs. controlled) and the likelihood of early dropout from the program. |
| Dawson et al [12], 2021 | Participants were randomly allocated, stratified by geographical location. | / | The study does not detail a specific analysis method or principle used exclusively for the attrition rate. The overall study analysis used logistic regression and linear models, with adjustments for local health district stratification |
| Grutzmacher et al [15], 2019 | Parents were recruited through school events and program promotional items sent home at the beginning of the school year. They enrolled by providing their phone number to program staff or enrolling themselves through a keyword texted to a short code. | Attrition data were recorded by the web platform used to send messages to participants. The program recorded when participants sent an SMS text message indicating they wanted to be removed from the program. Program enrollment and dropout dates were used to calculate program duration in days. | The study conducted survival analyses to examine differences in participant attrition. The primary predictor was the type of message sent, including stop messages. Kaplan-Meier curves were created for estimating differences in attrition for different message types, and Cox proportional hazards models were used for more detailed analysis. |
| Hawkes et al [44], 2023 | Participants were recruited either through referral from their general practice if they had non-diabetic hyperglycemia or via an online self-referral questionnaire that assessed risk factors such as age, gender, ethnicity, waist circumference, and bodyweight. | / | / |
| Howarth et al [18], 2019 | Employees from various departments were sent an email invitation to contribute to the development of the new intervention during work hours. Participation was voluntary, and no incentives were provided. | / | / |
| Jiang et al [45], 2023 | The recruitment process involved assessing all gastric cancer patients at the two study sites for eligibility from December 2022 to January 2023. A total of 24 post-discharged gastric cancer patients following gastrectomy were recruited over a four-week period. | Data were gathered upon hospital discharge (T0), and at 4 and 12 weeks post-discharge (T1 and T2). | / |
| Kaul et al [13], 2022 | Participants were recruited by phone and in person at a Weill Cornell Medicine/New York Presbyterian ambulatory care practice, which served over 5000 older adults. The recruitment methods included approaching individuals waiting for their appointments. | Mixed methods for data collection, including quantitative measures (surveys) and qualitative approaches (interviews). | / |
| Linardon et al [14], 2022 | Participants were recruited via an ED-related psychoeducational platform, which included a website and associated social media accounts. Eligibility was determined through an online screening measure. | Data was collected at baseline, 4 weeks, and 8 weeks post-randomization. | The analysis was intention-to- treat using linear mixed models for all outcome measures. Multiple imputations were used for handling missing data. |
| Paxton et al [17], 2017 | Recruitment utilized nonprobability sampling techniques including word of mouth, existing relationships with community-based organizations, and cases ascertained from tumor registries in the North Texas metropolitan area. | The study collected data on attrition rates through the tracking of participant dropout at the 3-month assessment, comparing completers and non-completers on lifestyle treatment-related variables and sociodemographic characteristics. | The study used descriptive statistics to analyze attrition, looking at differences between completers and non-completers in terms of lifestyle treatment- related variables and sociodemographic characteristics. |
| Plaete et al [46], 2016 | Adults (≥18 years) were recruited from 19 Flemish general practices. Participants were systematically allocated by a researcher either to the intervention group or to a waiting-list control group. In addition, GPs recruited adults for a separate intervention group. | Data were collected via evaluation questionnaires on health behavior. Self-reported fruit and vegetable intake were assessed at baseline, 1 week, and 1 month post- baseline. | / |
| Rom et al [16], 2023 | Participants were English-speaking women from the Australian community, recruited through online advertising (Facebook and HealthMatch clinical trial registry) between March and December 2021. | Data on attrition rates was collected through the eTherapy program's tracking system. It monitored participant engagement, completion of modules, and attendance at support sessions. | / |
| Schulz et al [8], 2013 | Participants were recruited through an online access panel, respondi AG. They received an email with a link to the intervention website (for the experimental group) or a web-based alcohol questionnaire (for the control group). | Data on attrition rates was collected through online follow-up measurements. The dropout rates at different stages of the study were analyzed, including initial engagement and follow-up participation. | The analysis of attrition rates involved logistic regression to identify differences in dropout rates between study conditions. The study compared dropout rates between different feedback strategies and examined demographic factors affecting attrition. |
| Silina et al [47], 2017 | Participants were selected from six randomly chosen family physicians’ practices of a health care center in Riga. They were invited during their visits to their primary care physicians or called after revising a list of patients in the appropriate age group. Those who agreed to receive SMS messages were randomized into the study. | / | / |
| Springer et al [48], 2018 | Participants were primarily recruited online through Craigslist, Nextdoor, Reddit, and an internal email list of a US West Coast research center. The study targeted participants in the San Francisco Bay Area. | A Kaplan-Meier survival curve and a Cox proportional hazards model were employed to examine attrition from the app | A Cox proportional hazards model coefficients was used to understand the impact of self- affirmation on attrition. |
| Van der Mispel et al [49], 2017 | Participants were recruited via general practice settings. Flyers and tablets in waiting rooms directed them to the intervention website. | Data was collected via LimeSurvey. Attrition was measured as nonusage attrition, occurring when participants stopped using the intervention or did not complete website sessions. | The study used descriptive analysis to explore attrition rates for different self-regulation components. Logistic regression analyses were conducted to identify predictors of intervention completion. |
| Whitley et al [50], 2020 | Participants were enrolled during baseline data collection at each church to reduce the sign-up burden. Contact information was collected from consent forms after verbal consent to participate in the messaging component. | / | / |
| Young et al [51], 2021 | Participants were recruited online through social media channels and targeted email campaigns by The Food & Mood Centre. They were directed to a recruitment website where they completed a screening survey. | Data on attrition rates were collected through system logs and data entries in the smartphone application. | The study analyzed attrition rates by examining the proportion of participants who remained active users over the course of the 8-week intervention. |
| Yuhas et al [52], 2023 | Caregivers were recruited through informational letters from school principals, flyers, and phone calls. They provided written or verbal consent for both themselves and their child. | The dropout rate was measured by tracking whether participants completed the first and second SMS assessments or notified the team of their desire to discontinue. Data was collected using the Qualtrics Research Suite. | The analysis used SPSS Version 27.0 with non-parametric tests due to the non-normal distribution of data. Chi-square and Mann-Whitney U tests examined caregiver baseline characteristics related to retention. |

**Table 4.** Trustworthiness: evidence of rigour (part 2).

| Author, year | Triangulation methods | Theoretical saturation | Linkage of findings to primary data | Reflexivity |
| --- | --- | --- | --- | --- |
| Brewer et al [11], 2016 | / | N | The study includes data to support its findings regarding the attrition rate, but does not provide extensive quotations or a variety of data sources specifically for this aspect. | The study acknowledges its limitations and ethical considerations, but does not explicitly discuss the effect of the researcher on the study. |
| Browne et al [42], 2020 | / | N | / | N |
| Cheung et al [43], 2019 | / | N | The findings about attrition rate are linked to the primary data collected from the study participants. However, the document does not provide detailed quotations from participants or identify speakers to ensure that the quotes represent a range of views. | The study acknowledges limitations and potential biases but does not extensively discuss the effect of the researcher on the study. |
| Coa & Patrick [10], 2016 | / | N | The findings on attrition rates are linked to primary data through the comparison of the characteristics of participants who completed the study to those who did not. However, the document does not provide extensive quotations or detailed primary data to support these findings. | The study acknowledges some limitations and ethical considerations but does not extensively discuss the effect of the researcher on the study. |
| Dawson et al [12], 2021 | / | N | / | The study considers the effect of the researcher on the research process. Limitations of the study, such as its short duration, reliance on self-reported data, and issues with generalizability, are acknowledged. Ethical considerations are also taken into account. |
| Grutzmacher et al [15], 2019 | / | N | The study presents data on retention rates and the impact of message types on attrition, but does not extensively quote individual participants or identify speakers, focusing more on overall trends and statistics. | The limitations of the study are acknowledged, including constraints in controlling for individual demographic characteristics and the potential impact of message content variations across schools. Ethical considerations are not explicitly discussed. |
| Hawkes et al [44], 2023 | / | N | / | N |
| Howarth et al [18], 2019 | / | ^a^N | / | The study considered the effect of the researchers on the study, identified limitations, and acknowledged that the research was not subject to ethical review as it involved voluntary co-designers. |
| Jiang et al [45], 2023 | / | N | / | While the study was registered at the Chinese Clinical Trial Registry, indicating ethical considerations, there is no explicit discussion of reflexivity or identification of limitations. |
| Kaul et al [13], 2022 | / | N | / | (1)Identification of Limitations: The study acknowledges several limitations. These include a small and convenience-based sample size, which limits generalizability, challenges in participant retention, and difficulty in ascertaining whether positive effects were due to specific aspects of the intervention or general support from health coaches. (2)Ethical Considerations: Ethical issues have been addressed. |
| Linardon et al [14], 2022 | / | N | The findings regarding the attrition rate are primarily supported by statistical data and analyses presented in the study. Direct quotes from participants or detailed qualitative data specific to attrition are not provided. | The study discusses its limitations and considers the potential impact of the research design and methods on the findings. Ethical considerations are also addressed. |
| Paxton et al [17], 2017 | / | N | The study links findings on attrition rate to primary data through descriptive statistics. It presents data comparing completers and non-completers but does not provide extensive direct quotations from participants. | The study considers the limitations of its methodology and sample representativeness. Ethical approval was obtained, and potential biases or effects of researchers on the study are acknowledged. |
| Plaete et al [46], 2016 | / | N | / | N |
| Rom et al [16], 2023 | / | N | The study links its findings on attrition rates to primary data collected through the eTherapy program, with specific details on the rates of dropout and reasons for disengagement. However, it does not specify if quotations are from diverse participants. | The study acknowledges its limitations and considers the effect of the researcher on the study. Ethical considerations are addressed. |
| Schulz et al [8], 2013 | / | N | The findings on attrition rates are supported by data presented in the study, including detailed statistics and analysis of dropout rates. However, the use of direct quotes from participants to support these findings is not evident in the document. | The study acknowledges the strengths and limitations of its methodology, the theoretical basis of the intervention, and the potential effect of the researcher on the study. However, a detailed reflexivity analysis is not provided. |
| Silina et al [47], 2017 | / | N | The study links the findings on the attrition rate directly to the data collected, primarily focusing on the number of participants who completed the study. It does not provide extensive quotations or individual participant data in this context. | The study discusses its strengths and weaknesses, considers the effect of the researcher on the study, and identifies limitations. Ethical issues were addressed with the approval of the Ethics Committee of Riga Stradins University. |
| Springer et al [48], 2018 | / | N | The study does link its findings to primary data, especially in terms of adherence and attrition. For example, it found that participants who received both the initial and booster affirmations were significantly more likely to meet their goals, indicating a link between self-affirmation and adherence | The study acknowledges its limitations but does not extensively discuss the effect of the researcher or ethical considerations beyond the initial IRB approval. |
| Van der Mispel et al [49], 2017 | / | N. | The study links findings to primary data, focusing on the attrition rates for various intervention components. However, it does not provide detailed participant quotations or identify speakers, as it relies on aggregate data analysis rather than individual accounts. | The effect of the researcher on the study and ethical issues are considered, but specific limitations are not detailed in the discussed sections. |
| Whitley et al [50], 2020 | / | N | / | The study acknowledges limitations like low response rates to the process evaluation questions and potential bias in responses. Ethical approval was obtained from RAND’s Human Subjects Protection Committee. |
| Young et al [51], 2021 | / | N | The study presents attrition rates and engagement data, but does not include direct quotes from participants or detailed qualitative data linking these rates to specific participant experiences or feedback. | The study acknowledges its limitations, including a large proportion of participants being from Australia, which may limit global generalizability. The effect of the researcher on the study and ethical considerations are addressed. |
| Yuhas et al [52], 2023 | / | N | / | N |

^a^N: no.

***Table 5.*** *Potential usefulness to synthesis (part 1).*

| Author, year | Representativeness | Linkage to existing data/theory | Alternative explanations |
| --- | --- | --- | --- |
| Brewer et al [11], 2016 | / | N | N |
| Browne et al [42], 2020 | / | N | N |
| Cheung et al [43], 2019 | / | N | N |
| Coa & Patrick [10], 2016 | / | Linked to the primary data collected from the study participants. | N |
| Dawson et al [12], 2021 | / | N | N |
| Grutzmacher et al [15], 2019 | / | Compared its findings with other literature on SMS text message programs but does not extensively cite other theories or studies in the context of attrition rate. | N |
| Hawkes et al [44], 2023 | / | N | N |
| Howarth et al [18], 2019 | / | ^a^N | N |
| Jiang et al [45], 2023 | / | N | N |
| Kaul et al [13], 2022 | / | N | N |
| Linardon et al [14], 2022 | / | Discussed in the context of other mental health app trials and compares its rates with those reported in other studies. | N |
| Paxton et al [17], 2017 | Discussed in relation to its composition (primarily African American breast cancer survivors) and how it relates to the broader population of minority breast cancer survivors. | Compared to existing literature, noting that its attrition rate is comparable to previous Web-based intervention studies but higher than recent studies conducted among cancer survivors. | N |
| Plaete et al [46], 2016 | / | N | N |
| Rom et al [16], 2023 | / | Discussed in the context of existing literature on online interventions and their challenges, including comparisons with other studies. | N |
| Schulz et al [8], 2013 | Discussed in terms of demographic characteristics, and comparisons are made between the experimental and control groups. | Referenced existing research and theories to contextualize its findings on attrition rates, particularly in relation to the effectiveness of web-based interventions and dropout rates. | N |
| Silina et al [47], 2017 | / | ^b^Y | N |
| Springer et al [48], 2018 | / | Referenced previous research and theories, particularly the work of Steele, the original author of the self-affirmation theory, to frame its findings within the existing body of knowledge. | N |
| Van der Mispel et al [49], 2017 | / | Referenced existing literature on eHealth interventions and self-regulation theory to contextualize its findings on attrition rates. | N |
| Whitley et al [50], 2020 | The sample primarily included older, female churchgoers from African American and Latino communities, reflecting the populations described in the introduction. | Referenced the Health Belief Model and compares findings with other studies and theories in health behavior change. | Y |
| Young et al [51], 2021 | / | N | N |
| Yuhas et al [52], 2023 | / | N | N |

^a^N: no. ^b^Y: yes.

***Table 6.*** *Potential usefulness to synthesis (part 2).*

| Author, year | Conceptual richness | Novel findings | Focus on population of interest | Focus on attrition question | Value to the synthesis |
| --- | --- | --- | --- | --- | --- |
| Brewer et al [11], 2016 | N | N | N | N | Moderate |
| Browne et al [42], 2020 | N | N | N | N | Low |
| Cheung et al [43], 2019 | N | N | Y | N | Moderate |
| Coa & Patrick [10], 2016 | N | N | Y | Y | Moderate |
| Dawson et al [12], 2021 | N | N | Y | N | Low |
| Grutzmacher et al [15], 2019 | N | N | Y | Y | Moderate |
| Hawkes et al [44], 2023 | N | N | N | Y | Low |
| Howarth et al [18], 2019 | ^a^N | N | N | ^b^Y | Low |
| Jiang et al [45], 2023 | N | N | N | N | Low |
| Kaul et al [13], 2022 | N | N | Y | Y | Moderate |
| Linardon et al [14], 2022 | N | N | Y | Y | High |
| Paxton et al [17], 2017 | N | N | Y | N | High |
| Plaete et al [46], 2016 | N | N | Y | N | Low |
| Rom et al [16], 2023 | N | N | Y | Y | High |
| Schulz et al [8], 2013 | y | N | Y | Y | High |
| Silina et al [47], 2017 | N | N | N | N | Moderate |
| Springer et al [48], 2018 | N | N | Y | Y | Moderate |
| Van der Mispel et al [49], 2017 | N | N | Y | Y | High |
| Whitley et al [50], 2020 | N | N | N | N | High |
| Young et al [51], 2021 | y | N | Y | N | High |
| Yuhas et al [52], 2023 | N | N | N | N | Moderate |

^a^N: no.

^b^Y: yes.

**Table 7.** Summary of scores for study appraisal (part 1).

| Subcategory | Study | Brewer et al [11], 2016 | Browne et al [42], 2020 | Cheung et al [43], 2019 | Coa & Patrick [10], 2016 | Dawson et al [12], 2021 | Grutzmacher et al [15], 2019 | Hawkes et al [44], 2023 | Howarth et al [18], 2019 | Jiang et al [45], 2023 | Kaul et al [13], 2022 |
| --- | --- | --- | --- | --- | --- | --- | --- | --- | --- | --- | --- |
| Transferability | Eligibility criteria | 1 | 1 | 1 | 0 | 1 | 1 | 1 | 0 | 1 | 1 |
|  | Country | 1 | 1 | 1 | 1 | 1 | 1 | 1 | 1 | 1 | 1 |
|  | Sample size | 2 | 1 | 2 | 3 | 3 | 5 | 6 | 2 | 1 | 1 |
|  | Age | 1 | 1 | 1 | 1 | 0 | 0 | 0 | 1 | 0 | 1 |
|  | Gender | 1 | 1 | 1 | 0 | 0 | 0 | 0 | 1 | 1 | 1 |
|  | Ethnicity | 1 | 0 | 1 | 1 | 0 | 0 | 0 | 0 | 0 | 1 |
|  | Socio-economic status | 1 | 0 | 0 | 1 | 0 | 1 | 0 | 0 | 0 | 1 |
|  | Other characteristics | 1 | 0 | 1 | 0 | 0 | 0 | 0 | 1 | 1 | 1 |
|  | Subcategory total score | 9 | 5 | 8 | 7 | 5 | 8 | 8 | 6 | 5 | 8 |
| Trustworthiness | Recruitment | 1 | 1 | 1 | 1 | 1 | 1 | 1 | 1 | 1 | 1 |
|  | Data collection | 1 | 1 | 1 | 1 | 0 | 1 | 0 | 0 | 1 | 1 |
|  | Analysis method and rationale | 0 | 1 | 1 | 1 | 1 | 1 | 0 | 0 | 0 | 0 |
|  | Triangulation methods | 0 | 0 | 0 | 0 | 0 | 0 | 0 | 0 | 0 | 0 |
|  | Theoretical saturation | 0 | 0 | 0 | 0 | 0 | 0 | 0 | 0 | 0 | 0 |
|  | Linkage of findings to primary data | 1 | 0 | 1 | 1 | 0 | 0 | 0 | 0 | 0 | 0 |
|  | Reflexivity | 2 | 0 | 2 | 1 | 3 | 1 | 0 | 3 | 1 | 2 |
|  | Representativeness | 0 | 0 | 0 | 0 | 0 | 0 | 0 | 0 | 0 | 0 |
|  | Linkage to existing data/theory | 0 | 0 | 0 | 1 | 0 | 1 | 0 | 0 | 0 | 0 |
|  | Alternative explanations | 0 | 0 | 0 | 0 | 0 | 0 | 0 | 0 | 0 | 0 |
|  | Subcategory total score | 5 | 3 | 6 | 6 | 5 | 5 | 1 | 4 | 3 | 4 |
| Usefulness | Conceptual richness | 0 | 0 | 0 | 0 | 0 | 0 | 0 | 0 | 0 | 0 |
|  | Novel findings | 0 | 0 | 0 | 0 | 0 | 0 | 0 | 0 | 0 | 0 |
|  | Focus on population of Interest | 0 | 0 | 1 | 1 | 1 | 1 | 0 | 0 | 0 | 1 |
|  | Focus on attrition Question | 0 | 0 | 0 | 1 | 0 | 1 | 1 | 1 | 0 | 1 |
|  | Subcategory total score | 0 | 0 | 1 | 2 | 1 | 2 | 1 | 1 | 0 | 2 |
| Value to the synthesis | Total score | 14 | 8 | 15 | 15 | 11 | 15 | 10 | 11 | 8 | 14 |
|  | Rating | Moderate | Low | Moderate | Moderate | Low | Moderate | Low | Low | Low | Moderate |

**Table 8.** Summary of scores for study appraisal (part 2).

| Subcategory | Study | Linardon et al [14], 2022 | Paxton et al [17], 2017 | Plaete et al [46], 2016 | Rom et al [16], 2023 | Schulz et al [8], 2013 | Silina et al [47], 2017 | Springer et al [48], 2018 | Van der Mispel et al [49], 2017 | Whitley et al [50], 2020 | Young et al [51], 2021 | Yuhas et al [52], 2023 |
| --- | --- | --- | --- | --- | --- | --- | --- | --- | --- | --- | --- | --- |
| Transferability | Eligibility criteria | 1 | 1 | 1 | 1 | 1 | 1 | 1 | 1 | 1 | 1 | 1 |
|  | Country | 1 | 1 | 1 | 1 | 1 | 1 | 1 | 1 | 1 | 1 | 1 |
|  | Sample size | 4 | 2 | 4 | 1 | 4 | 3 | 2 | 5 | 3 | 3 | 4 |
|  | Age | 1 | 1 | 1 | 1 | 1 | 1 | 0 | 1 | 1 | 1 | 1 |
|  | Gender | 1 | 1 | 0 | 1 | 1 | 0 | 1 | 1 | 1 | 1 | 1 |
|  | Ethnicity | 1 | 1 | 0 | 1 | 0 | 0 | 0 | 0 | 1 | 0 | 1 |
|  | Socio-economic status | 1 | 1 | 0 | 1 | 1 | 0 | 0 | 1 | 1 | 0 | 1 |
|  | Other characteristics | 0 | 1 | 0 | 1 | 1 | 1 | 0 | 1 | 1 | 1 | 0 |
|  | Subcategory total score | 10 | 9 | 7 | 8 | 10 | 7 | 5 | 11 | 10 | 8 | 10 |
| Trustworthiness | Recruitment | 1 | 1 | 1 | 1 | 1 | 1 | 1 | 1 | 1 | 1 | 1 |
|  | Data collection | 1 | 1 | 1 | 1 | 1 | 0 | 1 | 1 | 0 | 1 | 1 |
|  | Analysis method and rationale | 1 | 1 | 0 | 0 | 1 | 0 | 1 | 1 | 0 | 1 | 1 |
|  | Triangulation methods | 0 | 0 | 0 | 0 | 0 | 0 | 0 | 0 | 0 | 0 | 0 |
|  | Theoretical saturation | 0 | 0 | 0 | 0 | 0 | 0 | 0 | 0 | 0 | 0 | 0 |
|  | Linkage of findings to primary data | 1 | 1 | 0 | 1 | 1 | 1 | 1 | 1 | 0 | 1 | 0 |
|  | Reflexivity | 3 | 3 | 0 | 3 | 2 | 3 | 1 | 2 | 2 | 3 | 0 |
|  | Representativeness | 0 | 2 | 0 | 0 | 1 | 0 | 0 | 0 | 1 | 0 | 0 |
|  | Linkage to existing data/theory | 1 | 1 | 0 | 1 | 1 | 1 | 1 | 1 | 1 | 0 | 0 |
|  | Alternative explanations | 0 | 0 | 0 | 0 | 0 | 0 | 0 | 0 | 1 | 0 | 0 |
|  | Subcategory total score | 8 | 10 | 2 | 7 | 8 | 6 | 6 | 7 | 6 | 7 | 3 |
| Usefulness | Conceptual richness | 0 | 0 | 0 | 0 | 1 | 0 | 0 | 0 | 0 | 1 | 0 |
|  | Novel findings | 0 | 0 | 0 | 0 | 0 | 0 | 0 | 0 | 0 | 0 | 0 |
|  | Focus on population of Interest | 1 | 1 | 1 | 1 | 1 | 0 | 1 | 1 | 0 | 1 | 0 |
|  | Focus on attrition Question | 1 | 0 | 0 | 1 | 1 | 0 | 1 | 1 | 0 | 0 | 0 |
|  | Subcategory total score | 2 | 1 | 1 | 2 | 3 | 0 | 2 | 2 | 0 | 2 | 0 |
| Value to the synthesis | Total score | 20 | 20 | 10 | 17 | 21 | 13 | 13 | 20 | 16 | 17 | 13 |
|  | Rating | High | High | Low | High | High | Moderate | Moderate | High | High | High | Moderate |
