# Supplementary material for: Framework Development for Reducing Attrition in Digital Dietary Interventions: Systematic Review and Thematic Synthesis
Source: J Med Internet Res. 2024 Aug 27;26:e58735. doi: 10.2196/58735 (PMC11387916; doi:10.2196/58735)
Supplement: Multimedia Appendix 8 [file jmir_v26i1e58735_app8.doc]

**Multimedia Appendix 8: Data for Meta-analysis**

**Table 1. Data from randomized controlled trial.**

| Study | Control group | | | Intervention group | | |
| --- | --- | --- | --- | --- | --- | --- |
|  | Total (N) | Lost (N) | Attrition rate (%) | Total (N) | Lost (N) | Attrition rate (%) |
| Brewer et al [11], 2016 | / | / | / | / | / | / |
| Browne et al [42], 2020 | 12 | 3 | 25.0% | 8 | 5 | 62.5% |
| Cheung et al [43], 2019 | 20 | 12 | 60.0% | 40 | 21 | 52.5% |
| Dawson et al [12], 2021 | 43 | 6 | 14.0% | 87 | 9 | 10.3% |
| Jiang et al [45], 2023 | 12 | 2 | 16.7% | 12 | 1 | 8.3% |
| Kaul et al [13], 2022 | 13 | 4 | 30.8% | 18 | 7 | 38.9% |
| Linardon et al [14], 2022 | 195 | 130 | 66.7% | 197 | 130 | 66.0% |
| Plaete et al [46], 2016 | 149 | 88 | 59.1% | 277 | 199 | 71.8% |
| Schulz et al [8], 2013 | 135 | 60 | 44.4% | 313 | 165 | 52.7% |
| Silina et al [47], 2017 | 64 | 4 | 6.3% | 65 | 2 | 3.1% |
| Springer et al [48], 2018 | / | / | / | 127 | / | / |
| Yuhas et al [52], 2023 | / | / | / | 357 | 41 | 11.5% |

**Table 2. Data from observational study.**

| Study | Total (N) | Lost (N) | Attrition rate (%) |
| --- | --- | --- | --- |
| Coa & Patrick [10], 2016 | 193 | 83 | 43.0% |
| Grutzmacher et al [15], 2019 | 972 | 139 | 14.3% |
| Hawkes et al [44], 2023 | 1826 | 1159 | 63.5% |
| Paxton et al [17], 2017 | 37 | 13 | 35.1% |
| Rom et al [16], 2023 | 19 | 5 | 26.3% |
| Van der Mispel et al [49], 2017 | 422 | 330 | 78.2% |
| Whitley et al [50], 2020 | 131 | 6 | 4.6% |
| Young et al [51], 2021 | 128 | 90 | 70.3% |
