# Supplementary material for: Framework Development for Reducing Attrition in Digital Dietary Interventions: Systematic Review and Thematic Synthesis
Source: J Med Internet Res. 2024 Aug 27;26:e58735. doi: 10.2196/58735 (PMC11387916; doi:10.2196/58735)
Supplement: Multimedia Appendix 9 [file jmir_v26i1e58735_app9.doc]

**Multimedia Appendix 9: Thematic Synthesis**

***Table 1.*** *Thematic synthesis of the reason of attrition.*

| Theme/Sub-theme | | Descriptive theme | Effect | Perspectives from included studies | |
| --- | --- | --- | --- | --- | --- |
| Motivation | | | | | |
| High motivation | | Higher autonomous motivation | ^a^D | “A one unit increase in autonomous motivation was associated with lower odds…of early dropout, which persisted after adjusting for level of controlled motivation” [10, p. 3].  “Enrolment methods that require personal contact with the researchers may maximize retention because it attracts more motivated participants and allows expectations to be clarified between participant and researcher because it attracts more motivated participants and allows expectations to be clarified between participant and researcher” [17, p. 9].  “Users with higher autonomous motivation had significantly lower odds of dropping out within the first 2 weeks” [10, p. 1]. | |
|  |  | Motivation waning | ^b^I | “Additionally strict timelines on participation (e.g. expiry of program access after 4 weeks of non-participation) meant that users were much less likely to engage as motivation waned” [18, p. 4]. | |
| Attitude | | | | | |
| Negative experiential attitude | | Lack of interest | I | “In a community study, by 4 to 10 weeks, some children lost interest, and a few parents were tired of using the Mandolean, particularly setting up, dishing food onto a plate, and weighing food” [42, p. 11].  “…interventions being presented in an uninteresting manner followed by an excessive level of reading” [18, p. 4].  “The high attrition rate was attributed to participants having a lack of interest in completing the post-questionnaire” [14, p. 776]. | |
|  |  | Strict timeline | I | “…strict timelines on participation (e.g. expiry of program access after 4 weeks of non-participation) meant that users were much less likely to engage as motivation waned” [18, p. 4]. | |
| Negative instrumental attitude | | Limited usefulness | I | “Three intervention participants (3%) requested text messages to be paused, reasons given included…and having enough information” [15, p. 12].  “…users may have got what they wanted from the programme, e.g., they had developed habits to continue maintaining their lifestyle changes and no longer felt they needed the app” [44, p. 9]. | |
|  |  | Doubt regarding efficacy | I | “…skepticism that lifestyle modifications could mitigate pain” [13, p. 6]. | |
| Positive instrumental attitude | | Goal-connection feeling | D | “Moreover because this mobile messaging program was integrated into a larger church-based intervention which itself was developed using a community-based participatory process participants may have felt more connected to the intervention and its goals” [50, p. 10]. | |
|  |  | Clearer expectation | D | “Enrolment methods that require personal contact with the researchers may maximize retention because it…allows expectations to be clarified between participant and researcher” [17, p. 9]. | |
| Subjective/Injunctive norm | | | | | |
| Lack of subjective/injunctive norm | | No direct contact | I | “…participants could enrol and complete this trial entirely online, meaning that there was no need to come into contact with the researchers. This is important, as attrition in app trials is substantially higher when participants complete the trial online rather than when they are required to undergo telephone or in-person interviews with the researchers” [17, p. 9]. | |
| With subjective/injunctive norm | | With patient-provider relationship | D | “Contrary to our expectations, the drop-out rate was less than 10%. This could be related to the fact that patients were selected from one health centre and they had an already established patient–physician relationship” [47, p. 268]. | |
| Cue | | | | | |
| Distraction cue | | Triggered by stop messages | I | “Receiving a stop message increased the probability of attrition compared with receiving messages about nutrition, physical activity, or other topics” [15, p. 1].  “…the interaction between stop messages and the total number of stop messages received yields the largest effect on attrition compared with the interaction between other message types and the number of stop messages received” [15, p. 7]. | |
| Reinforcement | | | | | |
| Delayed reinforcement | | Delayed feedback | I | “In the second strategy, advice is given in a more traditional way at the end of the session (ie, after the last question has been completed). This method may lead to postponement of dropout—provided that the questionnaires are not too long—because respondents have to wait until the end of the questionnaire before receiving tailored feedback” [8, p. 2]. | |
| Positive/Immediate reinforcement | | Positive feedback | D | “In the first strategy [alternating], questions and advice are given…thereby motivated to continue. Such alternation might also enhance the attractiveness of the program” [8, p. 2].  “Other factors may include use of positive encouraging messages. Prior research found that participants preferred such content and our participants confirmed this in their feedback” [50, p. 10].  “These respondents may have felt rewarded by receiving the advice in-between answering the questions, and this strategy may have made the program more attractive” [8, p. 2]. | |
|  |  | No reimbursement | I | “…we did not offer participants reimbursement for remaining in the trial” [17, p. 9]. | |
|  |  | Immediate feedback | D | “In the first strategy [alternating], questions and advice are given alternately, so that the respondents are rewarded while they are still filling in the questionnaires and are thereby motivated to continue” [8, p. 2].  “Lower attrition rates during the first visit indicate that the version of the intervention with alternating questions and advice may be preferred” [8, p. 7].  “These respondents may have felt rewarded by receiving the advice in-between answering the questions, and this strategy may have made the program more attractive” [8, p. 2]. | |
| Resource | | | | | |
| With cognitive burden | Overwhelmed tasks | | I | “Attrition levels were higher for filling out questionnaires (e.g., to generate tailored feedback) than for the more interactive components (such as action planning, coping planning, etc.). The highest amount of attrition could be observed when people were shown the advice and asked to make their own action plan” [49, p. 9].  “Dropout analyses indicated that participants who chose more than one behavior were more likely to drop out” [46, p. 11].  “…interventions being presented in an uninteresting manner followed by an excessive level of reading, both of which made programs difficult for users to understand” [18, p. 4].  “Perceived task burden in combination with behavioral issues may have contributed to attrition” [42, p. 2].  “Self-regulation capacity of adults’ has been shown to be limited and it might be difficult for adults to make multiple behavior changes at the same time” [46, p. 11].  “The subsequent high attrition within the group suggests that psychosocial issues in combination with the intervention burden discussed earlier may have played a role in early attrition” [42, p. 12].  “This method may also increase the risk that the participant becomes overwhelmed by the amount of information he or she receives all at once” [8, p. 2]. |  |
| Low usability | Technical/Usability issues | | I | “Challenges reported by survivors included repeat calls from the automated phone system and ALIVE email messages not being fully interactive within certain email domains (ie, AOL, Thunderbird, Live, Outlook, and Lotus) nor on mobile phones or tablets” [17, p. 10].  “Low exposure to the experimental intervention was explained by poor acceptability of Mandolean as a home-based tool for treatment” [42, p. 2]. | |
| Limited knowledge/skills | Limited guidance | | I | “…desire for additional input from participants' primary care providers regarding the intervention targets” [13, p. 6]. | |
|  | Limited technical literacy | | I | “…technical literacy/time constraint issues” [13, p. 6]. | |
| Service resource | Lack of social support | | I | “…lack of social support…have all been cited as signiﬁcant barriers to participation in healthy living programs and behavior” [43]. | |
|  | More health services | | I | “The degree of rurality also had a significant effect on the probability of attrition, with metropolitan county participants more likely to drop out of the program than rural county participants” [15, p. 1]. | |
|  | Fewer health services | | D | “Participants in rural areas might be less likely to drop out of a text-based health promotion program as rural areas tend to have fewer health services and programs available” [15, p. 2].  “This finding may be explained by the relative scarcity of health services and programs in rural areas. Participants in rural areas may be less likely to drop out of a text-based health promotion program, as rural areas tend to have fewer health services and programs available” [15, p. 7]. | |
| Financial resource | Financial barriers | | I | “…ﬁnancial difﬁculties and…have all been cited as signiﬁcant barriers to participation in healthy living programs and behavior” [43, p. 12].  Financial barriers [13, p. 6]. | |
|  | Higher income | | D | “…there was a significant difference in dropout among respondents with different levels of income; the dropout rate was lower in respondents with the highest income compared to those with the lowest income…” [8, p. 6]. | |
| Time resource | Time constraint | | I | “…technical literacy/time constraint issues” [13, p. 6].  “The current study additionally noted that being primiparous was predicted of low engagement in the study” [43, p. 12].  “The demands of work and childcare have all been cited as signiﬁcant barriers to participation in healthy living programs and behavior” [43, p. 12].  “Three intervention participants (3%) requested text messages to be paused, reasons given included being too busy to read messages” [15, p. 12]. | |
| Personal state | Health/Life issue | | I | Control group: died (n=4), requested withdrawal (n=2); intervention group: died (n=3), transplanted (n=5), moved (n=1) [15, p. 12].  “…one participant was discontinued from the study due to the emergence of secondary symptoms which required more intensive support” [16, p. 9].  “The high attrition rate was attributed to participants…hospitalization, or death” [14, p. 776].  “…the other withdrew due to co-occurring mental health concerns after completing module one” [16, p. 8].  “The subsequent high attrition within the group suggests that psychosocial issues in combination with the intervention burden discussed earlier may have played a role in early attrition” [42, pp. 11–12].  “…two participants in the control group were lost to follow-up…because of intestinal obstruction (n = 1)” [45, p. 10]. | |
| Individual difference | | | | | |
| Cultural factor | | Cultural barriers | I | “…cultural expectations have been cited as signiﬁcant barriers to participation in healthy living programs and behavior” [43, p. 12].  “We found that women of South Asian background were less likely to complete the study, and greater dropout from a study of wearable activity monitors has also been observed amongst people of Indian ethnicity in Singapore” [43, p. 12]. | |
| Education level | Low education level | | I | “Our dropout analyses indicated that participants with low education were more likely to drop out” [46, p. 11].  “The fact that more lower educated adults participated in our study can perhaps explain the higher dropout rates” [46, p. 11]. | |
|  | High education level | | D | Caregivers with college education or more were retained at a higher rate [52]. | |
|  | | Non-specific or unclear |  | “A further two participants did not adhere per-protocol (i.e., did not attend three consecutive support sessions) and were considered disengaged after completion of module one and module two” [16, pp. 8–9].  “…one cited changed availability before commencing the program” [16, p. 8].  “…one participant of the intervention group was lost to follow-up because of refusing to complete the results measurement at T2” [45, p. 10].  “…percent drop-out rate was low. This may reflect the relatively passive nature of our intervention as participants could have stopped reviewing the messages without requesting to drop-out” [50, p. 10].  “Respondents who did not revisit the program after 6 months were those who evaluated the program more negatively at baseline, implying selective dropout” [8, p. 11].  “The high attrition rate was attributed to participants…absence from the center the day of the post-questionnaire assessment” [14, p. 776].  “…two participants in the control group were lost to follow-up because either they could not be reached or…” [45, p. 10].  “…users may have disengaged with the programme due to a lack of satisfaction with the intervention features” [44].  “Using call attempt data and Qualtrics notification regarding non-functioning numbers, it was determined that 11 (3%) were non-functioning numbers once the intervention started” [52, p. 5]. | |

^a^D: a decrease in attrition rate.

^b^I: an increase in attrition rate.

***Table 2.*** *Thematic synthesis of the potential solutions of attrition.*

| Theme | Sub-theme | Descriptive theme | Solutions from included studies |
| --- | --- | --- | --- |
| Boost and maintain motivation |  | Enhance autonomous motivation | “Applying SDT-based strategies to enhance autonomous motivation might reduce early dropout rates which can improve program exposure and effectiveness” [10, p. 1].  “…these design features might be motivating” [18, p. 5]. |
|  |  | Enhance self-affirmation | “One solution may be psychological interventions called self-affirmation exercises…Self-affirmation exercises are activities in which individuals focus on and affirm personally important values” [48, p. 2]. |
| Improve attitude | Improve experiential attitude | Make intervention fun | “…future interventions should…come across as a fun activity from the very start” [18, p. 4].  These design features might be “less boring” [18, p. 4]. |
|  | Improve instrumental attitude | Educate on intervention | “Future studies could examine whether providing more education about the evidence demonstrating relationships between lifestyle modification and pain mitigation could potentially enhance adherence and retention outcomes” [13, p. 8].  “Increasing the education participants receive (e.g., in the first session) about why tracking data are critical to the success of the program” [13, p. 9]. |
| Offer subjective/injunctive norm |  | Utilizing health practitioners' referrals | “…future research could examine various ways in which health coaches delivering the intervention could seek input from (or partner with) participants’ primary care providers. Such initiatives could serve to reassure participants about the safety and potential beneﬁt of the recommended lifestyle changes” [13].  “…partnering with health practitioners to refer and follow-up on the use of such an m-Health program may assist in improving non-usage attrition and dropout attrition in the real-world context. Our analysis of engagement across all versions of the My Food & Mood program found that users who were referred to our program by a health practitioner were more likely to use the program for the 8 week period of the study” [51, p. 275].  “Referral to use of the intervention by health practitioners” [51, p. 275]. |
| Eliminate distraction cue |  | Limit stop-messages | “Program planners needing to send instructions on how to drop out of a program should consider limiting the number of times this information is provided, as it is possible that a greater frequency of such information has negative implications for program retention” [15, p. 7]. |
| Provide immediate reinforcement |  | Set progress markers | “…the addition of a feature where milestones and successes could be acknowledged was recommended” [15]. |
|  |  | Provide immediate information | Suggest intervention with alternating questions and advice [8, p. 1].  “…using adaptive, just-in-time support, including live human and peer encouragement, during the intervention to promote continued engagement” [52, p. 7]. |
| Provide matching resources | Decrease cognitive burden | Make intervention easy | “…‘bite sized’ chunks of information instead of lengthy reading or time commitments were recommended” [18, p. 4].  “…presentation could be improved by keeping the look and feel of interventions simple with ‘less text more images’” [18, p. 4].  “Simpliﬁcation of data collection is important” [43, p. 12]. |
|  |  | Improve usability | “…future interventions should be easy to access at any time” [18, p. 4].  “Further technical and usability studies are needed to improve adherence in our patient group in the tertiary setting” [42, p. 2]. |
|  |  | Provide guidance | “Increasing the education participants receive (e.g., in the first session) about why tracking data are critical to the success of the program and ensuring that participants receive adequate training in how to enter these data may ameliorate resistance to tracking and improve overall tracking adherence” [13, p. 9].  “Solutions to address non-usage and early dropout attrition include…more guided instructions prior to commencing a trial with this m-Health program” [51, p. 275]. |
|  | Provide financial resource | Address financial barriers | “Helping to cover the costs of dietary supplements recommended by the Mymee health coaches warrants future research as do eﬀorts to provide more training to individuals with limited technical literacy” [13, pp. 8–9]. |
|  | Improve personal state | Improve emotional state | “An even greater focus on support…may be helpful as women may become frustrated by health care providers telling them what to do, without being aware of their full circumstances” [43, p. 12].  These design features might be emotionally engaging [18, p. 4]. |
| Provide social support |  | Using peer encouragement | “Using adaptive, just-in-time support, including live human and peer encouragement, during the intervention to promote continued engagement” [52, p. 7]. |
| Personalization strategy |  | Employ targeted strategies | “…targeted strategies can be employed” [52, p. 7]. |
|  | Based on feedback | Refine text-messages | “Further customisation, perhaps based on feedback during the course of their participation, may help reﬁne the text-messages delivered to each individual to suit their circumstances” [43]. |
|  |  | Solicit user-feedback | “These targeted strategies may include soliciting specific user-feedback in the design on the interventions” [52, p. 7]. |
|  | Attend to individual difference | Screen participants | “Solutions to address non-usage and early dropout attrition include a confirmation of participation step or opportunity to opt-out after watching an introductory video” [51, p. 275].  “To preempsively increase engagement, caregivers can be screened for phenotypes that match these characteristics…” [52, p. 7]. |
|  |  | Understand the impact of participants’ characteristics | “…a better understanding of the ways in which participants’ characteristics impact probability of dropping out after receiving a stop message could also improve retention. In particular, certain participants might be more responsive to stop messages, such as those who join many text-based programs and those with limited facility with SMS text messaging who might drop out accidentally or misunderstand the intention of the stop message. Furthermore, future research may identify the best ways of informing participants about how to remove themselves from the program and illuminate which groups of participants may be expected to already know how to remove themselves from any SMS text message program” [15, p. 7].  “If limited program availability in rural areas is both a motivation to use SMS text message programs with isolated populations and an explanation of high retention, related characteristics of limited access to resources, health disparities, and isolation such as socioeconomic status, race or ethnicity, immigration status, basic literacy, and exposure to other programs should be examined in future research” [15, p. 7]. |
|  |  | Address cultural barrier | “Whilst we tailored the dietary text messages for South Asian women, greater consideration needs to be given to cultural issues in general, to improve engagement” [43, p. 12]. |
| Dynamic intervention |  | Provide tailored follow-up | “Tailored automated follow-up and prompts to direct users back to the program based on their engagement metrics” [51, p. 275]. |
|  |  | Non-specific or inefficient | “Modify trial design for a shorter period. Make self-monitoring tool usage mandatory for a shorter period of time” [51, p. 275].  “Our pilot study has demonstrated that a healthy lifestyle intervention based around text messaging and activity monitors is suitable for some women with recent GDM, but major protocol modifications are required to improve completion rates for a larger trial to be feasible” [43, p. 12].  “…suggestions of those studies were followed to prevent dropout by providing personal feedback, facilitating goal setting and self-monitoring of behavior, the use of periodic email reminders and incentives, and the provision of counselor support. However, the dropout rate in the GPs’ intervention group was as high as in the researchers’ intervention group” [46, p. 11].  “These findings are also consistent with previous research emphasizing the importance of message characteristics, including content and the number of SMS text messages received, in achieving high retention rates in SMS text message–based health promotion programs” [15, p. 7]. |
