# Supplementary material for: Framework Development for Reducing Attrition in Digital Dietary Interventions: Systematic Review and Thematic Synthesis
Source: J Med Internet Res. 2024 Aug 27;26:e58735. doi: 10.2196/58735 (PMC11387916; doi:10.2196/58735)
Supplement: Multimedia Appendix 10 [file jmir_v26i1e58735_app10.doc]

**Multimedia Appendix 10: Analytical Themes and Eysenbach's Attrition Factors**

**Table 1. Analytical themes and Eysenbach's attrition factors [9] ^a^**

| Analytical theme | Analytical sub-theme | Proposed (hypothetical) factors influencing attrition [9] |
| --- | --- | --- |
| Motivation |  |  |
| Habit/Impulsive behavior |  |  |
| Attitude |  | Quantity and appropriateness of information given before the trial leading to unrealistic expectations. |
| Perceived norm |  |  |
|  | Descriptive norm |  |
|  | Subjective/Injunctive norm | Personal contact (on enrolment, and continuous contact) via face-to-face or phone increases perceived subjective/injunctive norm, while virtual contact does not.  "Push" factors lead to pressure from perceived subjective/injunctive norm, such as research assistants chasing participants. |
| Cue |  | "Push" factors such as automatic reminders from mobile applications.  Unnecessary external events |
| Reinforcement |  | Positive feedback, buy-in and encouragement from change agents and (for consumer health informatics applications) from health professionals / care providers. |
| Resources |  | The abundance of alternative resources leading to ease of dropout/stop using it from intervention. |
|  | Cognitive resource | Workload, for example, to fill in the follow-up questionnaires may create such a burden that participants drop out  Experience of the user |
|  | Usability | Usability and interface issues  Competing interventions with higher ease of use. |
|  | Knowledge/Skills |  |
|  | Service resource |  |
|  | Financial resource | If the intervention has been fully paid for upfront, they are less likely to abandon it, as opposed to interventions paid on a fee-per-usage basis, which are more likely to see dropout.  Competing interventions with lower costs. |
|  | Time resource | Time required  Competing interventions with less time consuming |
|  | Personal state |  |
| Individual differences |  |  |
| Provide social support |  | Networking effects/peer pressure, peer-to-peer communication, and community building (open interactions between participants).  Being able to obtain help from others |
| Personalization strategy |  |  |
| Dynamic intervention |  |  |

^a^Some factors may be linked to multiple themes. For instance, various forms of competing interventions can be associated with themes like usability, time resource, and financial resource.
