# Supplementary material for: Framework Development for Reducing Attrition in Digital Dietary Interventions: Systematic Review and Thematic Synthesis
Source: J Med Internet Res. 2024 Aug 27;26:e58735. doi: 10.2196/58735 (PMC11387916; doi:10.2196/58735)
Supplement: Multimedia Appendix 11 [file jmir_v26i1e58735_app11.doc]

### Multimedia Appendix 11: Force-Resource Model

#### Overview

The force-resource model comprises two subsystems that interact to influence behavior and contribute to attrition. The first subsystem, the driving force system, includes themes of motivation, perceived norms, personal agency, attitude, and habit/impulsive behavior, which collectively guide behavioral directions and trends. Participants are driven by this force system to engage in dietary interventions to improve health. Their behaviors are influenced by attitudes toward the diet-health connection and the efficacy of the intervention, as well as perceived norms and personal agency. This behavioral tendency underpins their initial participation and intention to persist.

The second subsystem is the supporting resources system. The core concept of this system is resource, defined as entities either intrinsically valued or instrumental in achieving valued ends. These resources include physical and psychological states, financial support, time availability, and accessible health services [41]. Adequate resources alongside the force system likely facilitate behavior change, whereas insufficient resources obstruct it. Interestingly, an excess of resources can also contribute to attrition, as it may diminish the perceived value of the intervention. This is attributable to the diverse and competitive nature of motivation; when resources are abundant, previously unattainable desires become attainable, leading participants to pursue more appealing activities, necessitating greater cognitive resources to overcome them. For instance, in food-rich environments, pursuing weight control goals as a self-regulation process demands more cognitive resources than pursuing eating enjoyment [53]. Therefore, ensuring participants have access to appropriate and ample supporting resources is critical to prevent attrition.

#### Driving Force System

##### Overview

The driving force system encompasses themes of motivation, perceived norm, personal agency, attitude, and habit/impulsive behavior, collectively guiding the direction or trends of behaviors (path 1).

##### Motivation

“Motivation can be defined as that which gives behavior its direction or goals, and determines the strength or energy behind that behavior” [59]. It evolves dynamically, manifesting as intention when linked with specific behaviors [60], leading to actions through a reflective process [39], affected by attitude, perceived norms, and personal agency (paths 3–5) [20]. Inadequate stimulation of intrinsic motivation by interventions often results in reduced user engagement and subsequent attrition (the first type of path 1). Human motivation is diverse, with individuals simultaneously harboring multiple motivational drives competing for their limited personal resources [61,62]. If an intervention lacks sufficient appeal, resources are diverted towards more prioritized motivations, such as the preference for enjoyable foods over dieting [53]. Effective interventions must, therefore, harness adequate intrinsic motivation to help individuals prioritize participation over competing motivations.

##### Habit/Impulsive Behavior

Habit/Impulsive Behavior operates under the impulsive process, characterized by low flexibility and rapid execution with minimal cognitive demand and no requirement for attentional resources [39]. Digital dietary interventions aim to facilitate behavior change, but unhealthy habit/impulsive behaviors can significantly hinder this goal, leading to attrition (the second type of path 1). Motivation can facilitate habitual/impulsive behaviors by lowering their execution threshold (path 2) [39]. Additionally, related cues can easily trigger such behaviors. Therefore, it is crucial for digital intervention designs to carefully avoid fostering factors that contribute to the occurrence of these behaviors.

##### Attitude

Attitude plays a critical role in shaping an individual’s motivation/intention to engage in a behavior (path 3) [20], subsequently influencing attrition rates. According to Glanz et al, “Attitude is determined by the individual’s beliefs about outcomes or attributes of performing the behavior (behavioral beliefs), weighted by evaluations of those outcomes or attributes” [20]. Attitudes are categorized into experiential, relating to emotional responses toward the behavior, and instrumental, concerning the perceived utility and outcomes of the behavior [20].

##### Personal Agency

Personal agency in behavioral psychology involves the self-acting autonomously on its own behalf [78], encompassing perceived behavioral control and self-efficacy [20]. “Perceived behavioral control refers to people’s perception of the ease or difficulty of performing the behavior of interest” [60], influenced by available resources and opportunities [60]. Perceived self-efficacy is the belief in one’s capability to achieve specific performance levels that impact their life [64]. Personal agency can positively or negatively impact motivation/intention (path 4) [20], thereby affecting attrition rates.

##### Perceived Norm

Perceived norm significantly shapes behavior through two types: subjective/injunctive norms and descriptive norms. Subjective/injunctive norms are based on normative beliefs about whether significant others approve or disapprove of the behavior, weighted by the motivation to comply with these referents [20]. Descriptive norms, on the other hand, involve perceptions of behaviors common within one’s social or personal networks [20]. Both types of norms contribute to social pressures [65-67], acting as motivational forces derived from the need for belongingness or esteem. Thus, perceived norms can either positively or negatively influence motivation and, consequently, attrition rates, highlighting their importance in the context of motivation (path 5) [20].

##### Cue

Cue refers to specific environmental stimuli triggering actions [54], including reflective-process behaviors and habit/impulsive behaviors (path 6) [68]. Cues can either facilitate continued participation or disrupt engagement. For instance, Grutzmacher et al. [15] found that receiving a stop message increased attrition probability compared to messages on nutrition or other topics. Intervention strategies must avoid disruptive cues and highlight beneficial ones to foster sustained participation.

##### Reinforcement

Reinforcement enhances the likelihood of a behavior by delivering a rewarding stimulus immediately after the behavior [55]. Immediate positive reinforcement supports the development of attitudes, perceived norms, personal agency (path 7) [57,69-71], and motivation [57], promoting participation in interventions. This is crucial in health interventions, where benefits are often delayed while costs are immediate [58]. Therefore, the timeliness of reinforcement is crucial for minimizing attrition rates; otherwise, its effectiveness will quickly diminish [72]. Schulz’s study demonstrates that alternating advice with questions can boost engagement by providing immediate relevant feedback during questionnaire completion, thereby motivating continued participation [8].

#### Supporting Resources System

##### Overview

Resources, defined as entities either intrinsically valued or instrumental in achieving valued ends, include physical and psychological states, financial support, time availability, and accessible health services [41]. These are crucial for maintaining participation in interventions (path 8). Inadequacies or mismatches in resources can heighten the risk of attrition, even among motivated participants. Furthermore, the resource system can influence attrition rates by impacting the force system, particularly affecting personal agency (path 9). Adequate external resources can alleviate perceived behavioral control challenges [20,60], while a positive health state and adequate cognitive resources can bolster self-efficacy [73]. Therefore, ensuring participants have access to appropriate and ample supporting resources is critical to prevent attrition.

##### Personal State, Finance, Time, Service

Several studies have identified that personal state, including emerging physical or mental illnesses and even death, contribute to participant attrition. Additionally, financial constraints caused attrition in interventions requiring monetary investment [43], while higher household income was associated with better retention [52]. Time constraints, particularly among individuals with demanding work and childcare responsibilities, emerged as significant barriers to participation in healthy living programs and behavior change [43]. The alignment of available health services with intervention content also plays a crucial role; in rural areas, where health services are scarce, a text-based health program was highly valued, leading to lower attrition rates. In contrast, urban participants, having access to a plethora of health service options, showed a higher tendency to disengage [17]. Therefore, ensuring participants’ optimal personal state, financial resources, time availability, and access to suitable health services is essential for sustained engagement. Lack of these resources or their misalignment with participants’ needs can significantly increase the risk of attrition.

##### Cognitive Resources

Cognition, recognized as a type of resource [74,75], encompasses functions such as attention, perception, imagery, language, working memory, semantic memory retrieval, episodic memory encoding and retrieval, priming, and procedural memory [76]. Interventions often demand specific cognitive tasks from participants, including answering questionnaires, interviews, reading materials, and maintaining attention, thereby necessitating cognitive resource allocation. Excessive tasks can lead to significant cognitive load. Individuals inherently strive to preserve, protect, and enhance their cognitive resources [41], viewing the potential or actual loss of these resources as a threat [41]. Withdrawal from interventions can thus be seen as a mechanism of self-protection.

Digital interventions typically require some degree of digital literacy [13]. However, poorly designed interventions that are hard to navigate and troubleshoot can deter participation [42,52], imposing undue cognitive load. Similarly, interventions requiring extensive reading can overwhelm participants, increasing attrition rates [18]. Plaete’s study further illustrates that participants engaged in multiple behaviors were more prone to attrition [46], attributed to the heightened demand for cognitive resources for self-regulation [63,77,78]. Based on our review, potential solutions to minimize cognitive resource burden encompass enhancing the usability of digital interventions (path 10) through improved design, simplifying tasks, limiting excessive activities within short periods, avoiding multiple simultaneous behaviors, and offering ample guidance and instructions to bolster knowledge and skills (path 11).

#### Individual Differences

Individual differences, primarily referring to stable personal attributes [56], including demographic backgrounds, personality traits, and cultural values, indirectly yet broadly affect both systems within the model (path 12). For instance, people with varying levels of education exhibit differences in motivation, attitudes, personal agency, and various resources, particularly cognitive resources, ultimately impacting attrition.
