# Supplementary material for: Framework Development for Reducing Attrition in Digital Dietary Interventions: Systematic Review and Thematic Synthesis
Source: J Med Internet Res. 2024 Aug 27;26:e58735. doi: 10.2196/58735 (PMC11387916; doi:10.2196/58735)
Supplement: Multimedia Appendix 12 [file jmir_v26i1e58735_app12.doc]

### Multimedia Appendix 12: Implications for Digital Health Equity

#### Overview

Participant attrition arises from mismatches between individual resource and force systems. When this phenomenon expands to population level, it essentially creates a form of digital health inequity due to disparities in access to digital health resources. Digital health equity strives for equitable access to and utilization of resources such as digital health technologies, training programs, digital healthcare systems, and community support structures, all designed to improve health outcomes universally [92,93]. In promoting digital health equity, reducing attrition rates is a key strategy [92]. This encourages us to broaden the goal of reducing attrition to encompass a larger population and to design solutions at more comprehensive levels based on the multilevel determinants in digital health [92,93].

#### Intervention-Level

This level emphasizes the usability and accessibility of digital tools to enhance the effectiveness and compliance of interventions. Therefore, in addition to the previous strategies of user-friendly design, behavior-factor activation, personalized adaptation, and dynamic follow-up, there should be a special focus on the characteristics of different populations. For instance, in countries with multicultural backgrounds, the design of digital systems should enhance cultural and language diversity to make them accessible to diverse populations.

#### Individual-Level

This level focuses on literacy training. We recommend adopting diverse educational formats tailored to differences in cognitive levels, learning styles, cultural backgrounds, and available resources to ensure accessibility across diverse populations. For instance, for busy young adults, online courses may be a more accessible format, while face-to-face teaching may be more suitable for older adults with lower digital literacy.

#### Community-/Social-Level

This level should encourage the utilization of resources such as media and social networks to increase publicity and guidance, promoting the normalization of social support as a common mode of interpersonal interaction and community operation. This includes fostering community-led health initiatives and integrating digital tools into existing community support structures [92]. Additionally, the advent of digital technology has significantly deepened and expanded social networks. Strengthening such health-focused social networks can stimulate motivations rooted in the needs for belongingness or esteem, thereby increasing engagement and reducing attrition rates. Furthermore, intervention design should thoroughly consider the social network of the recipients, as social networks play a crucial role in either promoting or constraining behaviors [94]. This is particularly evident in microsocial relationship systems [95], such as families, where individuals have interconnected action scripts [96]. Therefore, it is imperative to fully harness the positive influences of social networks to enhance the engagement and effectiveness of digital health interventions.

#### Systems-Level

This level emphasizes creating a supportive environment that allows digital health resources to be effectively implemented and accessed by all populations, especially underserved groups. This includes enhancing investment and fostering leadership collaboration in healthcare systems [92]. Additionally, the importance of data resource sharing should be prioritized. Governments and health departments could establish a national health data sharing platform that allows for the cross-institutional and regional sharing of patient health information, which would enhance the continuity of health interventions and reduce patient attrition due to information gaps. Simultaneously, expert services constitute a vital supportive resource within the healthcare system. They can provide targeted guidance based on the characteristics of different populations, ensuring that digital health resources reach various regions and demographic groups. For instance, appointing experts with relevant cultural backgrounds to serve minority ethnic groups and specialists in particular medical fields to cater to specific patient populations could enhance service delivery.

#### Policy-Level

This level advocates for comprehensive, cross-sectoral health policies to remove structural barriers to accessing digital health resources and to guide the rational allocation of these resources. To this end, we recommend industry guidance and performance evaluation. Industry guidance primarily targets companies and institutions that provide healthcare services. The government could support these efforts with targeted funding or tax incentives to encourage the provision of specialized services to areas or populations lacking health resources. For example, providing interest-free loans for internet access projects in remote areas and including digital health services for specific disease populations under the coverage of public health insurance. Performance evaluation involves establishing robust metrics to assess digital health equity, such as incorporating attrition rates as a key indicator for classification, and incorporating these into performance evaluations for local governments, especially public health departments. This aims to guide digital health resources towards underprivileged groups, ensuring they benefit from digital interventions
